# Non-Binary People’s Sexuality, Sexual Health, and Relationship Satisfaction: A Review of 12 Years of Quantitative Research (2012–2024)

**DOI:** 10.1007/s10508-025-03224-0

**Published:** 2025-09-02

**Authors:** Fraedan Mastrantonio, Hanna Kovshoff, Heather Armstrong

**Affiliations:** School of Psychology, University of Southampton, Southampton, SO17 1BJ UK

**Keywords:** Non-binary, Transgender, Sexuality, Gender identity, Sexual satisfaction

## Abstract

Non-binary is a term used by individuals to describe their gender identity, which is better conceptualized outside the man–woman binary. In recent years, research interest in the sexuality of gender minority individuals has grown, as shown by the increasing number of publications on this topic. Accordingly, a comprehensive systematic review which synthesizes the range of sex-related variables captured in research studies with non-binary people is needed. This analysis examined 12 years of quantitative sex research, aiming to (1) outline what is known about the sexuality, sexual health, and relationship satisfaction of non-binary people; and (2) appraise measures used in this literature for the inclusivity of non-binary identities (focusing on language and terminology). Searches were conducted on PsycINFO, Web of Science, and MEDLINE for papers in Italian or English between 2012 and 2024, and 44 articles were included for synthesis. Papers covered a wide range of topics, showing that non-binary people often report non-monosexual sexual identities (e.g., queer, pansexual), more attraction to and relationships with other non-binary individuals, and similar levels of sexual and relationship satisfaction as binary transgender people. However, the language and tools employed within the research were not always expansive or inclusive enough to adequately represent non-binary people’s experiences. For example, gender minority individuals were often grouped together for analysis, hiding likely within group differences. Future research should use gender-neutral language and measures and consider non-binary individuals separately, in order to better understand their specific sexual health, well-being, and relationship needs and outcomes.

**Supplementary Information:**

The online version contains supplementary material available at 10.1007/s10508-025-03224-0.

## Introduction

“Non-binary” is an umbrella term that describes individuals whose gender identity is outside the binary of man–woman (Richards et al., 2016). More specifically, “non-binary” refers to a group of gender identities that disturbs or questions the prevalent understanding of gender as a binary construct (Vijlbrief et al., 2020). Some of the experiences described by non-binary people include, but are not limited to, perceiving one’s gender as completely outside of man or woman, identifying as a man or a woman only some of the time, or not feeling they are any gender at all (American Psychological Association, 2015; Budge, 2017; Matsuno & Budge, 2017). Non-binary identities have been documented across various societies throughout history, although understandings, perceptions, levels of acceptance, and availability of expansive language vary widely in different cultures (Gonzalez‐Salzberg & Perisanidi, 2021; Oh et al., 2024). Non-binary people may use a range of identity labels to characterize their experience of gender, such as genderfluid, agender, bigender, pangender, and genderqueer among many others (Twist & de Graaf, 2019).

Often, non-binary people are conceptualized as part of the transgender community. Transgender (or trans) is a word used to describe the identity of someone who identifies with a gender that does not match the sex they were assigned at birth (American Psychological Association & National Association of School Psychologists, 2015). Transgender is often used as an umbrella term to describe individuals who experience their gender identity as either binary (i.e., trans men or women) or non-binary. Importantly, although non-binary people may use the term transgender to refer to themselves (e.g., Wilson & Meyer, 2021), this population is extremely diverse, and non-binary individuals’ gender expansiveness means that both people who identify as transgender and people who do not can exist within the non-binary gender spectrum. Among non-binary people, reasons for not identifying with the label trans might link to feelings of unworthiness toward claiming a trans identity (i.e., not feeling “trans enough”) or perceiving relevant differences between one’s own identity and experiences and those of binary transgender people (Darwin, 2020). These observations highlight the need to consider binary trans and non-binary people as different gender identity groups in sex and health studies.

Nonetheless, although others have noted the importance of recognizing that the intersection between one’s gender, self-identification, and sexuality can shape individual health needs (Zeeman et al., 2017), sex research has a long history of either ignoring the existence of non-binary people or grouping non-binary and binary transgender people. This approach masks potential differences between the groups and limits our understanding of the nuances in gender identity and health needs between these populations (Smalley et al., 2016). More recently, some authors have begun to recognize this gap in the literature and have started to compare non-binary and binary trans people on health-related variables such as gender transition (e.g., Kennis et al., 2022a), mental health (e.g., Jones et al., 2019), and sexual health and sexuality (e.g., Holt et al., 2023; Perez & Pepping, 2024), with some differences emerging on variables such as sexual fluidity (Katz-Wise et al., 2016), orientation, and attraction (Boskey & Ganor, 2022).

Although sexual health has long been recognized as a broad and multifaceted construct (e.g., Edwards & Coleman, 2004), sex research with gender-diverse populations has historically been characterized by a narrow and medicalized focus (Anzani & Prunas, 2023). It has been argued that inclusive sex research with transgender and non-binary people fails to properly represent the experiences of those whose identity challenges gender binarism, possibly due to a lack of adequate methodologies and measures, as well as underpowered sample sizes (Gil-Llario et al. 2021; Lindley et al., 2022; van Anders, 2015).

As this field is evolving rapidly, a clearer understanding of the existing literature on the sexuality and sexual health of non-binary individuals is needed to better comprehend and evaluate the state of the science, how non-binary people are being identified and included in sex research, and if and how their experiences are being captured.

### Current Systematic Review

The current systematic review synthesizes 12 years of quantitative research on the sexuality and sexual health of non-binary populations, including a wide range of sexual constructs (such as sexual orientation, fluidity, sexual well-being, and sexual satisfaction), to develop a broader understanding of the literature in this area and to acknowledge the multifaceted and complex nature of sexuality and sexual health. We chose to include articles published in the last 12 years to capture recent and current understandings and conceptualizations of non-binary identities. More specifically, we decided to focus on research published after 2012 for two main different reasons:

1. From a research perspective, 2012 marks the year the US Gallup poll collected data related to sexual orientation and transgender identity for the first time (Gallup Organization, 2012). At the same time, within the UK context, the Trans Mental Health Study (McNeil et al., 2012) was published by the Scottish Transgender Alliance, representing the first comprehensive study including a variety of health variables and healthcare-related experiences for trans and non-binary people within the UK setting (Ellis et al., 2015).
2. From a generational perspective, we wanted to include studies with millennials (individuals born between 1981 and 1996) as well as gen Z (born between 1997 and 2012) as participants. This is because younger individuals are more likely to identify as non-binary (Statistics Canada, 2022). Taking Canada as an example, in 2021, through the Canadian census, it was reported that 0.19% of participants over 15 were transgender and 0.14% were non-binary (Statistics Canada, 2022). 62% of these individuals reported being younger than 35, with non-binary people’s mean age being especially low compared to the general Canadian sample over 15 (30 years old vs. 48 years old; Statistics Canada, 2022). Moreover, the proportion of trans and non-binary people was between three and seven times higher between gen Z and millennials compared to older generations (Statistics Canada, 2022).

This review adds to existing systematic reviews (e.g., Marshall et al., 2020; Özer et al., 2023) on sexual health and sexuality of gender-diverse individuals by focusing on psychological literature and databases, summarizing results of the examined studies, and including quantitative studies on relationship satisfaction (to highlight the interconnectedness of sexual and relationship satisfaction and the benefits of studying them concurrently). Additionally, while a recent systematic review addressed general health differences between non-binary and binary people (Scandurra et al., 2019), to our knowledge this is the first systematic review that focuses more specifically on non-binary people’s sexuality. Accordingly, this review aims to enhance our understanding of what is known about sexual orientation, sexual fluidity, sexual satisfaction, sexual health and well-being, sexual function and dysfunction, and relationship satisfaction within the non-binary population, while also considering differences and similarities with binary (trans and cis) people. A secondary aim is to assess which scales have been used to measure sexuality and relationships among non-binary people, and what terminology has been used to group and classify their gender identities.

More specifically, the underlying research questions of this review are:

1. What does quantitative research tell us about sexuality, sexual health, well-being, and relationship satisfaction/quality among non-binary adults?
2. How inclusive of non-binary people is quantitative sex research with respect to measurement and terminology?

## Method

For this systematic review, we adhered to the Preferred Reporting Items for Systematic Reviews and Meta-Analyses (PRISMA) guidelines (Page et al., 2021). Before starting the systematic search of literature, preliminary searches were performed to refine keywords and to better specify the constructs of interest. The review was registered on Prospero (protocol number: CRD42021290226) on 12/11/2021. Endnote software (version 20.2) was used to manage deduplication and the screening process of the papers (The EndNote Team, 2013). Eligibility criteria are presented in Table 1. We also note that due to a larger than expected number of search results returned, inclusion criteria 8–10 were added after the initial searches had been performed to refine the focus of this paper and provide a more cohesive review.

**Table 1:** Inclusion/exclusion criteria

Inclusion criteria	Exclusion criteria
1. Includes variables relating to sexuality, sexual health, or relationship satisfaction/quality (including sexual orientation, sexual fluidity, sexual satisfaction, sexual well-being, sexual function/dysfunction, orgasm, sexual fantasy, sexual preferences, sexual distress, and sexual behavior)	1. Exclude if only data on HIV/STI prevalence, treatment, or preventive measures (e.g., PrEP) are presented, as this is the focus of previous systematic reviews (e.g., Dang et al., 2022; Van Schuylenbergh et al., 2018)
2. Published in peer review publications	2. Grey literature, conference articles, theses, and other non-peer reviewed publications
3. Empirical analysis (primary research and secondary data analysis were included)	3. No empirical analysis
4. Directly involves non-binary and/or transgender individuals as participants	4. Exclude if study only reports others’ perceptions or opinions, such as family members or partners
5. Presents quantitative analyses (more specifically, used a quantitative measure to assess sexuality, sexual health, or relationship related constructs). Mixed-method studies that included a quantitative approach to data relating to sexuality and relationships were also included	5. Qualitatively analyses
6. Mean sample age over 16 years	6. Mean sample age below 16 years, focus on children
7. Published in English or Italian	7. Other languages were not included in this review
8. Published between 2012 and 2022 (initially, later updated to 2024)	8. Articles published prior to 2012 were not examined
9. Has separate analyses/descriptives for individuals that identify outside the gender binary (accepted labels were all labels relating to non-binary identities, such as "non-binary," "genderqueer," "agender," or labels that group multiple identities outside of men and women together, such as "other")	9. Papers that did not differentiate non-binary and binary trans or cis people were not included
10. Includes samples from Europe, North America, and/or Australia and New Zealand	10. Due to cultural differences in the understanding and categorizations of non-binary genders, papers referring to non-western samples were not included

### Information Sources

Initial searches were conducted on the databases PsycINFO, MEDLINE, and Web of Science in September 2022. Searches were then updated in August 2024. Hand searches were also performed by manually searching the reference lists of included papers.

### Search Strategy

The keywords (in title or abstract) used to perform the search are reported in Supplementary Table 1. Some of the keywords referring to gender are now obsolete and were included with the aim of capturing older or more medicalized literature.

### Selection Process

**Fig. 1:**
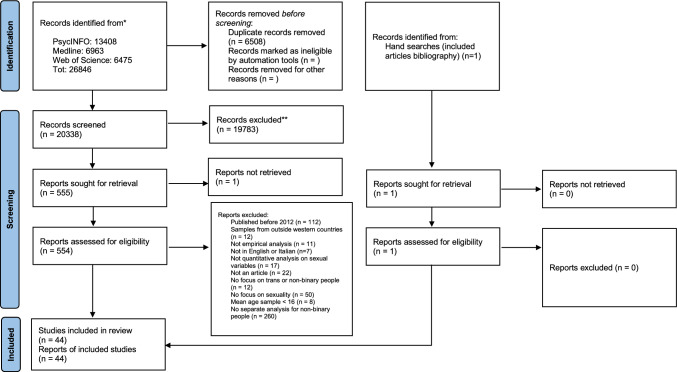
PRISMA chart for English searches

**Fig. 2:**
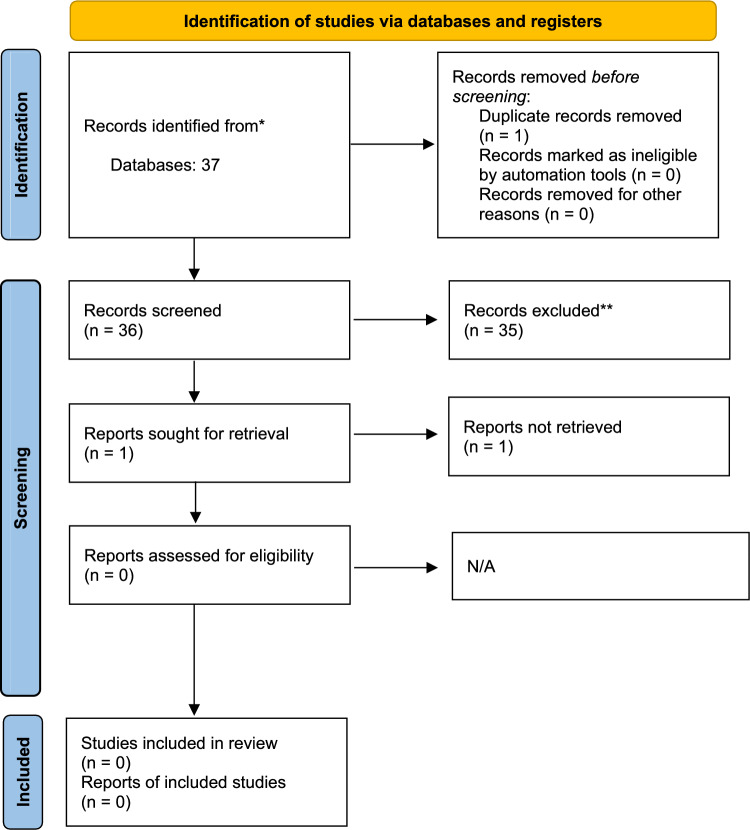
PRISMA chart for Italian searches

For the English papers, the initial number of retrieved articles in September 2022 was 22,864, of which 5535 were identified as duplicates. For the Italian articles, 36 were initially retrieved, of which one was a duplicate. The updated searches conducted in August 2024 returned a total of 3296 additional English papers and one Italian paper that had been published between October 2022 and August 2024. Duplicates were identified through a mix of manual screening and use of the Endnote software automatic screening tool. Overall, only one Italian language paper was included past the screening phase, but it was later excluded because the full-text could not be retrieved. (Figs. 1 and 2 show PRISMA charts for Italian and English searches combined for 2022 and 2024.)

After piloting inclusion and exclusion criteria, all titles, abstract and full texts were sourced and screened by the first author, while the last author screened 20% independently. Inter-rater reliability was calculated between 0.84 and 0.91 for each phase, and discrepancies were discussed until resolved. Any papers which were unclear as to their inclusion were also screened by both and and subsequently discussed.

After the initial search, 192 English language articles were identified. However, it is relevant to highlight that after screening for the inclusion of non-binary participants, 155 of these articles (80.7%) did not include any non-binary participants or did not report separate analyses for non-binary and binary transgender individuals and were therefore excluded. An additional 8 articles were excluded as they reported on samples from outside Europe, North America, Australia, and/or New Zealand. Lastly, one paper was identified through handsearching, resulting in 30 articles included after the initial search. During the search update, 97 papers were identified for possible inclusion; however, 52 of these (53.6%) were excluded as they did not separate between binary trans and non-binary participants. As such, an additional 14 papers were added following the search update, resulting in a total of 44 papers included in this review.

### Data Extraction

Data extraction was completed primarily by the first author in consultation with the other authors, following a standardized protocol based on a piloted and pre-agreed extraction table that included:

- Study date/location/authors
- Sample information and sampling method
- Demographics (e.g., gender identity of participants, age, ethnicity and race for whole sample and non-binary participants when available)
- Method of asking about gender identity and trans identity
- Sexual variables considered in the study (e.g., sexual orientation, sexual fluidity, etc.)
- Scales used to measure main sexuality constructs (completed by non-binary people)
- Main findings

After information was extracted, authors discussed any queries or uncertainties until consensus was reached. For extracted demographics, separate data were included for non-binary participants when available. If unavailable, only general sample characteristics were reported. For other variables, results were only reported if separate analysis for non-binary individuals were available.

### Study Risk of Bias Assessment

To appraise the quality of all included studies, the standard quality assessment criteria for evaluating primary research papers for quantitative studies was used (Kmet et al., 2004). This tool uses 14 criteria, and the reviewer assigns a score between 2 and 0 (yes, partially, no) for each, or selects “NA” if appropriate. Number of applicable responses (total possible sum) and scores obtained for each item (total sum) are then combined in a summary score (total sum/total possible sum). Papers with a summary score above 75% are classified as high quality, papers that score between 75 and 55% are classified as moderate quality, and studies that score below are 55% classified as low quality.

Two reviewers independently assessed a sample of papers (10% of total), and any disagreements were discussed until resolved. Inter-rater reliability was calculated at 0.74. After this, the first author completed the quality assessment in consultation with the second and third authors, with any doubts being discussed until consensus was reached.

### Data Analysis

For the primary research question, studies were grouped for synthesis based on the reported sexual and relationship related variables. Information relating to study design, study and participant characteristics, and gender identity assessment and non-binary labels was synthesized in a narrative manner.

For the secondary research question, we report which scales were used to assess sexuality, sexual health, and relationship satisfaction and whether these measures were inclusive of the non-binary population (e.g., whether they used gender-neutral language in terms of pronouns, body parts etc., or if they recognized the existence of genders outside of men and women—for instance when asking about sexual attraction).

## Results

### Quality Assessment

Most studies (39/44) were high quality, while five papers were classified as moderate quality. Total scores for each paper are shown in Table 2, together with study characteristics.

**Table 2:** Study characteristics and quality assessment summary scores

Citation	Location samples	Sampling method	Main independent variables	Main outcome variables considered	Scales or items used for main sexuality constructs (completed by non-binary people)	Main findings	Quality assessment
Almås et al. (2024)	Norway	Convenience sampling	Gender identity	Sexual behavior, Sexual arousal, Sexual satisfaction, Masturbation	Questionnaire adapted French, used in previous studies	**Sexual behavior:** Non-binary people reported a mean age of 12.8 (4.0) of first sex with oneself and of 17.8 (3.4) for first sex with others. Non-binary people were more likely to having had last sex with a trans or intersex person. **Sexual arousal:** non-binary people reported being more aroused by the idea of perceiving themselves as trans. **Sexual satisfaction:** male oriented people reported higher sexual satisfaction in terms of seducing and dominating their partners, but lower in relation to using clothing and other fetishes compared to non-binary oriented people. **Masturbation:** non-binary people reported higher rate in the past 12 months compared to female oriented people but not male oriented people. **Sexual practices:** majority of non-binary oriented people had engaged in kissing, masturbation and 42.4% had engaged in oral sex in their last encounter with a sexual partner	0.55
Anzani and Prunas (2020)	Not specified (possibly Italy as questionnaire in Italian)	Convenience sampling	Gender identity	Sexual fantasy	**Sexual fantasies:** Sexual Fantasy Questionnaire (SFQ)—Italian version. Confirmatory factor analysis performed, proposed structure did not fit the data (even when including cisgender participants). Principal axis analysis performed, 5 items were removed. Authors highlight this is a gendered scale, made for cisgender people and used due to lack of inclusive tools **Sexual attraction:** Kinsey scale (Erotic attraction from "Exclusively to men/masculinity Predominantly to men/masculinity, only incidentally to women/femininity" to "Predominantly to women/ femininity, only incidentally to men/masculinity Exclusively to women" + "Attracted to neither men/ masculinity nor women/femininity")	**Sexual fantasy:** no significant difference in scores for factors "Dominance and control" and "Forcing/humiliating the partner" across genders (cis men, cis women and non-binary people). Non-binary people reported less arousal for factors "Kink and non-normative sexual interests", "Attractive/irresistible partner", "Older, more experienced partners" then cisgender groups, and less arousal on factors "Group sex, multiple sexual partners, promiscuity" and "Passivity and submission" compared to cis men (no significant difference with cis women). Non-binary participants had less arousal for "Undressing, showing-of" and "Devotion/being devoted to partner" than cis women (no significant difference with cis men). **Sexual attraction:** 40% of non-binary people were "Equally attracted to men/masculinity and women/femininity" followed by 18.2% "Predominantly attracted to women/ femininity, only incidentally to men/masculinity"	0.91
Atkins et al. (2024)	USA	Convenience (Clients accessing Alabama-based AIDS Service)	Transactional sex	STIs diagnosis	**Transactional sex history:** “In the past five years, have you exchanged sex for money, drugs or something of need?”"	**Transactional sex:** of the 65 non-binary or other participants, 60 had no history of transactional sex	0.91
Bishop et al. (2023)	USA	Data from baseline of The Generations Study	Gender identity, race/ethnicity, gender non-conformity, cohort (age)	Sexual identity development milestones/profiles	**Sexual identity development milestones:** six questions assessing age at which milestones occurred (e.g., “At what age were you first sexually attracted to someone of the same sex as you?”). Participants responded with their age at each event or “never”/ “do not know/cannot recall” (missing). Participants were classified in one of four profiles: early adolescence (EA), middle adolescence (MA), late adolescence (LA) or adulthood (AH) profile. **Sexual identity:** options were gay or lesbian, bisexual, or other (i.e., same-gender-loving, pansexual, queer, asexual, and anti-label)	**Sexual identity development milestones:** Non-binary/genderqueer had the largest proportion of people in the middle adolescence (MA) profile (44%) and the fewest (9%) in the adulthood profile (AH) profile. Women and non-binary/genderqueer participants were more likely to be in the EA or MA profiles than the late adolescence (LA) profile compared to men	0.95
Boskey and Ganor (2022)	USA	Convenience sampling (patients of Center for Gender Surgery in USA)	Binary/ non-binary gender identity	Sexual orientation (identity, behavior, attraction)	Specific questions not reported in article	**Sexual orientation—attraction:** Non-binary participants were significantly more likely to have attraction toward non-binary genders (non-binary, genderqueer, and agender, *p* < .01 for all) than binary participants. No differences in attraction to binary individuals. **Sexual orientation—behavior:** non-binary people had more non-binary sexual partners ( *p* < .01). **Sexual orientation—identity:** non-binary people were more likely to identify as queer compared to binary trans men. Binary trans men were more likely to be heterosexual	0.86
Bosse and Chiodo (2017)	USA	Convenience sampling (community organizations and Internet groups)	Sex assigned at birth, “Congruent/incongruent” gender identity	Sexual orientation identity, “Congruent/incongruent” gender identity	Demographic questionnaire on gender identity, sex assigned at birth, sexual orientation (options asexual, bisexual, gay, lesbian, pansexual, queer, heterosexual and other)	No separate analysis for non-binary subsample aside from Sexual orientation (no inferential analysis on subgroups)	0.68
Burgwal et al. (2019)	Georgia, Poland, Serbia, Spain, and Sweden	Convenience sampling (snowball)	Gender identity and demographics, gender-affirming medical interventions	Minority status, self-reported health, general well-being	**Belonging to different minority groups (sexual minority status is the only relevant for this review):** Participants indicated whether they felt they belonged to minority groups (“No, I don’t belong to this group,” “Yes, but it is not important at all to me,” “Yes, but it’s only slightly important to me,” and “Yes, and it’s very important to me” – recoded into yes/no for each group). Minority groups listed were: ethnic minority, religious minority, sexual minority (gay, lesbian, bisexual, queer, asexual, etc.), disability)	**Sexual minority status:** Non-binary participants were more often identifying as a sexual minority (93.8% vs. 75.8%, *p* < .001)	0.91
Byrne et al. (2022)	New Zealand	Convenience sampling (online and community based organizations and networks)	Gender identity, Sexual attraction, Ability to negotiate barrier use	PrEP awareness	**Sexual attraction:** “Who are you sexually attracted to? Mark all that apply.” (trans men, cis men, trans women, cis women, genderqueer or non-binary people, none of the above, others.). **Ability to negotiate protective barrier use with sexual partners:** T-Barrier Scale (Dharma et al., 2019)	**Sexual attraction**: Non-binary AFAB attracted to men 164 (23.3); Non-binary AFAB not attracted to men 52 (7.5); Non-binary AMAB attracted to men 53 (7.6); Non-binary AMAB not attracted to men 19 (2.7). **Ability to negotiate barrier use:** In both bivariate and multivariate analysis, being non-binary AFAB and attracted to men linked to lower T-Barrier scale scores compared to trans women attracted to men. Significant gender differences are also reported in relation to proportions of participants reporting being “Somewhat certain” and “Completely certain” in response to specific T-Barrier items	0.95
Dahl et al., 2024	USA	Archival data	Demographics, belonging to The National Coalition for Sexual Freedom (NCSF), BDSM involvement	BDSM identity, fantasy, and behavior; coping and mental health	**BDSM fantasy and behavior:** BDSM-Fantasy (BDSM-F) Scale and BDSM-Activity (BDSM-A) Scale (created for this study, based on previous literature and consultation with NCSF)	**BDSM fantasy:** gender minority individuals (M = 44.58, SD = 16.39, Cohen’s d = .55) had statistically significantly higher Dominant Fantasy scores compared to cis women (M = 35.83, SD = 15.44), and similar scores to cis men. Submissive Fantasy scores were lower for cis men (M = 43.37, SD = 19.14) compared to other gender minority (M = 53.42, SD = 17.85, Cohen’s d = − .54), no differences reported between non-binary and cis women or trans binary people. **BDSM behavior:** Cis women (M = 30.40, SD = 13.63) reported significantly lower Dominant Behavior scores compared to other gender minority individuals (M = 38.28, SD = 15.84, Cohen’s d = − .53), no differences reported with trans binary and cis men. No significant differences reported between other gender minority people and other genders for Submissive Behavior	1.00
Dargie et al. (2014)	Not specified	Convenience sampling (various methods online and offline, snowball)	Gender identity	Social support, relationship quality, physical and mental health	**Sexual attraction and behavior:** Kinsey Scale (Kinsey et al., 1953, 2003). **Sexual identity:** Sexuality Questionnaire (SQ; Alderson, 2012) to rate level of identification (low–high) with identity labels. **Relationship satisfaction:** Hendrick’s Relationship Assessment Scale (RAS; Hendrick, 1988)	**Attraction/Behavior:** 0% of "other" gender identity group identified as "Exclusively Heterosexual" or "Homosexual", while all the other options were chosen by 25% of this subsample. Authors reported similar patterns between “other” gender and trans men, but no inferential analysis were performed. **Other sexual variables:** No separate analysis for non-binary subsample aside from Sexual orientation (no inferential analysis on this). **Relationship satisfaction:** no results reported for non-binary people	0.86
Dubin et al. (2021)	USA	De-identified EHR chart review study of patients (NYU Langone Health)	Gender identity	Sexual orientation identity	**Sexual orientation prompt:** “What is your current sexual orientation” (bisexual, choose not to disclose, don’t know, heterosexual/straight, homosexual/ gay, lesbian, and something else—text input not visible for this study)	No inferential findings	0.68
Eliason and Streed (2017)	USA	Convenience sample (professional social media networks and listservs)	Gender identity	Sexual orientation identity responses to NHIS question	**Sexual orientation identity:** National Health Interview Survey (NHIS) two-part question: ‘‘Which of the following best represents how you think of yourself?’’ ( lesbian or gay; straight; bisexual; something else; I don’t know the answer). Second part (if person responds “something else”) was “If you answered something else, what do you mean by something else?” (options: you are not straight, but identify with another label such as queer, trisexual, omnisexual, or pansexual; you are transgender, transsexual, or gender variant; you have not figured out or are in the process of figuring out your sexuality; you do not think of yourself as having sexuality; you do not use labels to identify yourself; you mean something else)	NA	0.91
Fisher et al. (2018)	USA	Convenience sampling (online advertisement)	Gender identity	Perceptions of patient/provider communications, gender and sexual minority (GSM) stigma, confidentiality concerns, GSM-sexual health information, sexual orientation (behavior and identity)	Ad-hoc questionnaire (questions developed after community consultation and piloting). **Sexual orientation—attraction and behavior:** items on gender of people participants are attracted to, number (lifetime and last 12 months) and gender of sexual partners (last 12 months). Response options were: cisgender male, cisgender female, transmasculine and transfeminine partners. **Sexual orientation—identity:** Both open-ended and multiple choice (could select more than one) item for label [full questions not reported]	**Sexual orientation identity:** non-binary youth was significantly more likely to identify as queer**. Sexual Behavior—Number sexual partner in lifetime/last 12 months:** no significant differences by gender identity. Non-binary people had a mean of 2.72 partners in the last 12 months and 4.94 partners in lifetime. **Sexual behavior—Gender of sexual partners:** no significant differences by gender identity are reported, the majority of non-binary participants had cisgender male partners, followed by transmasculine partners	0.86
Fuller and Riggs (2021)	USA	Convenience sampling (community organizations and online)	Gender identity	Partners, partners gender, relate to perceiving support, satisfaction, and hopefulness about future relationships, as well as psychological distress	**Relationship satisfaction:** Relationship satisfaction subscale of the gay and lesbian relationship satisfaction scale (GLRSS; Belous & Wampler, 2016)	**Relationship satisfaction:** Non-significant findings for gender differences for relationship satisfaction	0.77
Galupo et al. (2018)	USA	Convenience sampling (Online announcements, snowball)	Gender identity and sexual orientation (monosexual vs. plurisexual)	Scale ratings (face validity for Kinsey scale (Kinsey et al., 1953, 2003), Klein Sexual Orientation Grid (KSOG; Klein et al., 1985), The Sexual-Romantic Scale (Galupo et al., 2014) and The Gender Inclusive Scale (designed for current study)	**Sexual orientation identity:** question not explicitly stated. **Face validity for sexual orientation scales:** ‘‘This scale accurately reflects my sexuality’’ using a five-point Likert scale ranging from 1 (‘‘Strongly Disagree’’) to 5 (‘‘Strongly Agree’’)	In terms of gender, face validity analysis only compared cis and trans group, as trans subgroups were too small	0.95
Goldbach et al. (2023)	Most participants from North America and Western Europe	Convenience sampling (online)	Demographic variables (i.e., age, sexual orientation, education level, gender identity), contextual variables (i.e., body satisfaction, social dysphoria, fetishization), medical transition (i.e., hormone therapy)	Sexual experiences outcomes (i.e., receptive/insertive penetration, importance of sex, sexual pleasure, sexual intimacy)	**Sexual orientation/ Gender of current partner:** question not specified. **Sexual Activity Importance, Engagement, and Pleasure:** questions were part of survey created for the larger project. **Insertive/Receptive sex:** “Did you have receptive (either anal or vaginal) sexual intercourse?”/ “Did you have insertive (either with a penis or a strap-on) sexual intercourse (either anal or vaginal)?”(yes /no). **Global sexual experiences:** “Overall, I do perceive sexual activity as pleasurable”/ “Overall, I do perceive sexual intimacy as pleasurable”; and “Overall, sexual activity is an important part of my life.” (5-point Likert)	**Importance of sexual activity:** hierarchical linear regression performed, in first step of model, identifying as agender (*p* < .05) was one of the significant predictors, but it was not significant in final model. **Engagement in Receptive/Insertive Sexual Activity:** gender was not a significant predictor. **Pleasure from Sexual Activity and Intimacy:** gender identity was not a significant predictor. No other separate analysis	0.91
Goldberg et al. (2020)	USA	Data from The Generations study	Sexual identity, gender identity	Sexual attraction, sexual partnering, romantic relationships	**Sexual identity:** Phase 2 Generations baseline survey self-report with options: straight/heterosexual, lesbian, gay, bisexual, queer, same gender loving, or other (write-in). Results categorized in queer, bisexual, lesbian/gay and other (included same-gender loving, queer, asexual, pansexual, and anti-label). **Sexual attraction:** “How sexually attracted are you to the following types of people: Women, non-transgender; men, non-transgender; transgender women/male-to-female; transgender men/female-to male?” (5-point Likert) with creation of four variables based on different levels of attraction. **Sexual partnering:** “In the last 5 years, who did you have sex with? By sex, we mean any activity you personally define as sexual activity” (could select multiple answers and categories were created similarly to attraction). **Current partner gender:** options cisgender woman (CW), cisgender man (CM), and transgender, genderqueer/non-binary (GQNB)	**Sexual Identity:** 25% of genderqueer/non-binary participants were queer (significantly more than cis men and cis women) and 33% of queer participants were genderqueer/ non-binary. **Sexual Attraction:** Most non-binary people reported some attraction. 78% of non-binary people was attracted to both women and men. **Sexual partners**: no differences in the percentage of GQNB people who were sexually active in the last year. Among those who were sexually active, 50.6% of queer respondents had had both women and men as partners. **Romantic relationship:** No significant differences based on sexual identity in terms of being in relationship or gender of partner	1.00
Hibbert et al. (2020)	UK	Convenience sample (online advertisement)	Gender, demographic variables, psychosocial factors, condomless intercourse, drug use, loneliness, self-stigma, body dissatisfaction, life satisfaction, sexual contact without consent etc	Sexual health clinic attendance, reporting ever having an HIV test	**Sexual health clinic attendance:** question on attendance of a sexual health/genitourinary medicine clinic in the past 12 months. **Sexual behavior:** gender identity of sexual partners	**Sexual health clinic attendance**: 29% of non-binary people had attended a clinic in last 12 months (descriptive result). Being non-binary was not associated with sexual health clinic attendance in the past year. **Sexual behavior:** 56% of non-binary people had sex with men, 77% with women and 67% with non-binary people. Inferential analysis were done grouping trans people and comparing with cis people	0.91
Holt et al. (2023)	Australia	Convenience sampling (online advertisement and through trans organization)	Gender identity	Sexual satisfaction, romantic satisfaction	**Sexual satisfaction:** “I am satisfied with the sexual aspects of my life” (5-point Likert)—derived from the Multi-Dimensional Sexual Self-Concept Questionnaire (Snell, 2010). **Romantic satisfaction: “**I am satisfied with the romantic aspects of my life” (5-point Likert)—also from Snell (2010). **Sexual experience/Sexual behaviors:** exact items not reported. For behaviors, questions covered condomless sex and drug use in relation to sex	**Sexual orientation:** non-binary people were more likely to identify as fluid, queer or asexual compared to men and women. **Sexual experience:** Non-binary participants were more likely than binary participants to have been recently sexually active. Binary participants reported fear about one’s sex life more than non-binary participants. Non-binary participants were the most likely to have had non-binary partners. **Sexual and romantic satisfaction:** neither was independently related to gender. **Sexual Behavior:** non-binary people and trans binary people did not differ in terms of condomless intercourse in the last year. Non-binary participants were more likely to report drug use in relation to sexual activity	0.86
Jacobson and Joel (2019)	Majority: USA (70.2%), United Kingdom (14.1%), Canada (10.7%)	Convenience sampling (Online recruitment, LGBT organizations/ groups)	Gender identity, sex assigned at birth	Attraction to men/women, sexual fantasies, sexual behavior, relationships	**Sexual orientation (excluding identity):** Eight questions on sexual fantasies, sexual attraction, sexual behavior, and romantic relationships (each question repeated twice, once toward women, once toward men (e.g., “How would you rate the level of your sexual attraction to men?” and “How would you rate the level of your sexual attraction to women?”) **Sexual Identity**: options were Exclusively heterosexual, Mostly heterosexual, Bisexual, Mostly homosexual, Exclusively homosexual, Pansexual, Asexual or Other). This was not used due to some people referring to their SAAB (sex assigned at birth) and other to their current GI (gender identity) while responding	**Attraction to men/women:** significantly negatively correlated in whole sample, AFAB gender-diverse group had lowest correlation levels (*r* = − .06, *p* = .18). In AMAB gender-diverse individuals, the correlations between attraction to men and to women was not significantly different	0.95
Jann et al. (2022)	USA	Data from database from primary care health center	Gender identity (cisgender vs. TGNC), age, race/ethnicity, homelessness	Hepatitis A vaccinations, hepatitis B vaccinations, cancer screenings, HPV vaccination	Exact prompts not specified	**Sexual Health variables:** no inferential analysis separating binary/non-binary people (trans/non-binary people grouped together and compared to cis people)	0.91
Kattari et al. (2021)	USA	Healthy Kids Colorado Survey (HKCS) representative high school sample	Sexual orientation, gender identity, bullying and mental health	Sexual behavior	**Sexual orientation**: “Which of the following best describes you?” heterosexual (straight), gay or lesbian, bisexual, and not sure). Sexual orientation was combined with GI (nine categories). **Sexual behavior:** “Have you ever had sexual intercourse? (yes/no)”; “During your life, with how many people have you had sexual intercourse? (I have never had sexual intercourse, 1 person, 2 people, 3 people, 4 people, 5 people, 6 or more people)” and “How old were you when you had sexual intercourse for the first time? (I have never had sexual intercourse, 11 years old or younger, 12 years old, 13 years old, 14 years old, 15 years old, 16 years old, 17 years old or older).”	**Sex ever:** 0.63% of those who had sex identified as "transgender other". Of non-binary people 51 out of 81 had had sex. Non-binary people were grouped together with other trans people for inferential analysis	0.91
Kattari et al. (2019)	USA	Healthy Kids Colorado Survey (HKCS) representative high school sample	Gender identity and sexual orientation (controlling for bullying and mental health)	Sexual risk (drugs or condom use during sex)	**Sexual Orientation:** "Which of the following best describes you?" (heterosexual (straight), gay or lesbian, bisexual, and not sure)—responses for sexual orientation and gender identity were (nine categories). **Sexual risk—alcohol or drugs use:** “Did you drink alcohol or use drugs before you had sexual intercourse the last time? (yes/no)”. **Sexual risk – condom use:** “The last time you had sexual intercourse did you or your partner use a condom? (yes/no)”	**Sexual risk:** 42.11% of "Transgender Other" participants used drugs and alcohol before last intercourse and 61.40% did not use a condom. Only descriptive statistics available, as inferential analysis were conducted using GI*SO groups (transgender identity collapsed and divided in subgroups based on sexual orientation)	0.95
Katz-Wise et al. (2023)	USA	Sampling through Prodege panel	Demographics	Sexual Fluidity	**Sexual fluidity:** two items: 1) Sexual orientation identity change: “Have you ever experienced a change in your sexual orientation identity? Yes/No; 2) Attraction change: “Have you ever experienced a change in your attractions to others over time? Yes/No. **Sexual orientation identity:** “‘Sexual orientation’ describes who you are attracted to and how you identify yourself based on those attractions. Sexual orientation may change over the course of people’s lives. Which of the following best describes your current sexual orientation?” (straight/heterosexual, bisexual, gay or lesbian, pansexual, queer, asexual, not sure, another identity/identities—write in). Participants could pick one. **Sexual orientation attraction**: “‘Attraction’ describes sexual or romantic feelings toward another person. Attractions may change over the course of people’s lives. Who are you currently attracted to?”, with the following response options: girls/women, boys/men, non-binary people (e.g., genderqueer, gender non-conforming, another non-binary identity), people of another gender identity: (write-in). Participants could choose all that apply	**Sexual fluidity:** Non-binary individuals (73.1%) were more likely to report identity change compared to cisgender individuals. Gender identity was associated with changes in attraction (*p* < .01), with non-binary people or another identity (71.3%) reporting highest attraction change compared to other genders	0.77
Katz-Wise et al. (2016)	USA	Convenience sampling (online and in-person)	Gender identity, Social gender transition	Sexual fluidity in attraction	**Sexual orientation identity—**“How do you currently identify your sexual orientation?” (straight/heterosexual, gay/ lesbian/same-gender attracted, bisexual, queer, questioning, I do not label my sexual orientation, unsure, asexual, other, recoded as straight, gay/lesbian, bisexual, queer, and other/non-binary). **Sexual fluidity in attractions—**two items: “Have you ever experienced a change in attractions to others? (For example, feeling only attracted to women, then feeling attracted to both women and men)” (yes/no). If yes to first item, “Did you experience a change in attractions to others after recognizing you were transgender and/or gender nonconforming? (For example, feeling only attracted to women before transition, then feeling attracted to both women and men after transition)” (yes/no)	**Sexual Fluidity:** 42% of non-binary participants experienced a change in attraction in their lifetime. Individuals who experienced change in attraction were more likely to be non-binary. When only considering those who socially transitioned (Adjusted model on 205 participants), 26.1% of those that changed their attractions post-transition were non-binary (*p* = .30). Those who did experience a change were less likely to be non-binary	0.77
Kennis et al. (2022b)	Most participants Netherlands (54.4%), USA (18.8%), Belgium (12.9%)	Convenience sample (mainly online social media for trans people and broader university and sex research community for cis people, snowball)	Gender identity	Attraction to men/women, sexual well-being, sexual body worries specific to trans people, sexual self-concept discrepancies	**Sexual well-being—**multiple scales were used: Sexual Esteem measure taken from larger questionnaire on sexual self-concept (Buzwell & Rosenthal, 1996; adapted by Deutsch et al., 2014). Four subscales included (Behavior, Body Perception, Attractiveness). **Trans-specific body image worries during sex:** T-WORRY (Dharma et al., 2019). **Sexual satisfaction:** Global Measure of Sexual Satisfaction (GMSEX; Lawrance & Byers, 1995). **Sexual self-concept discrepancies:** sliding scale 0 to 100, higher scores indicating a higher SSC discrepancy. **Sexual orientation:** two sliding scales (one for men, one for women) indicating levels of attraction to these genders (0 to 100, lower scores indicating lower attraction)	**Attraction to men/women:** cisgender group scored lower than binary trans and NBGQ group for attraction to women (both *p* < .001), but no significant differences for attraction to men. **Sexual well-being:** comparisons on the four sexual self-esteem components, T-WORRY, and sexual satisfaction. The only significant difference between non-binary (NBGQ) and trans binary (TB) participants was for T-WORRY (TB higher score), while only a "trend towards difference" was found for sexual satisfaction (*p* = .066, non-binary had higher score). Non-binary people had lower score on all variables compared to cisgender people, but only significant difference was for Sexual esteem body perception. **Sexual self-concept discrepancies:** NBGQ scored significantly higher than cisgender group (*p* < .001), but not different from the binary transgender group	0.86
Kennis et al. (2022a)	mainly The Netherlands, Belgium, USA	Convenience sampling (mainly online, snowball)	Gender identity (binary vs. non-binary), desire for treatment (Fulfilled/Unfulfilled)	Undergone GAMT, GAMT desire/motives, gender dysphoria, satisfaction with life, anxiety and depression, sexual satisfaction, transgender-specific body image worries, and sexual self-concept discrepancies	**Sexual orientation:** 2 sliding scales (one for men, one for women) to indicate level of attraction (0 to 100, with lower scores indicating lower attraction). **Sexual satisfaction:** Global Measure of Sexual Satisfaction (GMSEX; Lawrance & Byers, 1995). **Sexual Self-Concept Discrepancies:** two items—“Think about your actual sexual self-concept, and your ideal (item 1)/ought (item 2) sexual self-concept. Your actual self-concept entails all the ideas and feelings you have about who you currently are as a sexual person. Your ideal sexual self-concept entails all the ideas and feelings you have about who you ideally would want to be (item 1)/ who you should be (item 2) as a sexual person. How far away is your actual sexual self-concept from your ideal (item 1)/ ought (item2) sexual self-concept?” (0 = ‘Entirely overlapping’ to 100 = ‘Very far away,’ with higher scores indicating a higher SSC discrepancy). **Transgender-specific body image worries (in relation to sex):** T-WORRY	**Sexual Orientation:** Binary and non-binary transgender groups did not differ in terms of attraction to men/women. **Other sexual constructs:** inferential analysis done by separating in Unfulfilled desire/Fulfilled desire for GAMT (gender-affirming medical treatment) and not based on binary/non-binary identity	0.91
Koós et al. (2024)	42 countries	Convenience sampling (online, through local research networks)	Demographics	Pornography use/motivation (PUMS scale validation)	**Pornography use:** Pornography Use Motivations Scale (PUMS; Bőthe et al., 2021). Pornography and sexuality questions: past-year frequency of their Porn use and masturbation (0 – 10) & the average duration of their Porn use	**Pornography use motivations:** non-binary people had lower latent means than men for most motivations. Non-binary people scored higher than women on all motivations. For self-exploration, latent mean of non-binary people was higher than both men and women. All differences were significant (*p* < .01)	1.00
Lin et al. (2024)	42 countries	Convenience sampling (online, through local research networks)	Demographics	Sexual Distress	**Sexual Distress:** Short Sexual Distress Scale (SDS-3; Pâquet et al., 2018)	**Sexual Distress:** Study focuses on scale validation. On a descriptive level, observed mean reported for gender-diverse individuals (M = 3.76; SD = 2.87)) is higher than the observer mean for men (M = 3.41; SD = 2.75) and women (M = 3.15; SD = 2.66)	1.00
Littman et al. (2024)	USA	Convenience (out of 1462 veterans identified as trans/ non-binary, 14,000 were randomly selected to contact)	Demographic variables	Gender-affirming medical procedures	**Sexual orientation:** not specified in paper	**Sexual orientation:** trans men were more likely than non-binary people to identify as heterosexual/straight	0.91
Mark et al. (2018)	Not specified, possibly North America	Convenience sampling (online and print advertisements)	Attachment style	Relationship satisfaction, sexual satisfaction, and sexual desire	**Sexual satisfaction:** General Measure of Sexual Satisfaction Scale (GMSEX; Lawrance & Byers, 1995). **Relationship satisfaction:** General Measure of Relationship Satisfaction (GMREL; Lawrance & Byers, 1992). **Sexual desire:** Dyadic Subscale of the Sexual Desire Inventory (SDI-D; Spector et al., 1996)	**Sexual and relationship satisfaction**: in bivariate analysis no significant differences between gender categories (men, women, genderqueer, *p* > .05). **Sexual desire**: significant main effect of gender in sexual desire, but difference in post hoc was only between men and women and not genderqueer participants. Other analysis were not conducted separating gender and sexual orientation categories, as preliminary analysis did not support the examinations of each category separately (no significant effect of gender, sexual orientation or gender x sexual orientation)	1.00
McKenna et al. (2021)	USA	Convenience (online and offline, e.g., LGBTQ + organizations	Non-binary status, traditional gender self-concept, sexual assertiveness	Sexual consent attitudes	**Sexual Assertiveness:** Sexual Assertiveness Questionnaire (SAQ; Loshek & Terrell, 2015). Gender-neutral pronouns were added to make more inclusive. **Sexual consent attitudes, beliefs and behaviors:** The Sexual Consent Scale-Revised (SCS-R; Humphreys & Brousseau, 2010). Confirmation factor performed, original structure not adequate fit (single factor with Principal component analysis, four items removed), internal consistency calculated for sample	**Sexual assertiveness:** no significant differences between cisgender and non-binary participants. In non-binary participants, sexual assertiveness was significantly correlated with number of partners (*r* = .29, *p* < .05). **Sexual consent attitudes:** non-binary participants had significantly less maladaptive consent attitudes, beliefs and behaviors than cis people (*p* < .001). Identifying as non-binary was also significantly negatively correlated with age (*p* < .05) and with sexual consent (*p* < .01). For non-binary participants, being older and having had more partners was associated with more adaptive consent attitudes, beliefs and behaviors. Being non-binary predicted less maladaptive consent attitudes, beliefs and behaviors. **Number of sexual partners:** non-binary people had penetrative sex with a mean of 9.90 partners in their life (no significant differences with cis participants)	0.88
Nadarzynski et al. (2023)	UK	Convenience sampling/ snowball (online)	Demographics	Casual sex	**Casual sex:** questions around engagement in casual sex with one or more people after lockdown started; how long they were able to abstain from casual sex, if they re-engaged with sexual partners in lockdown, reasons for meeting sexual partners during lockdown	**Casual sex in lockdown:** 28% of non-binary people engaged in casual sex. Gender was not associated with casual sex engagement during lockdown	0.91
Nimbi et al. (2024)	Italy	Convenience (online and through local asexual organizations)	Demographics, sexual orientation – asexual spectrum	Sexual desire	**Sexual desire:** The Sexual Desire Inventory-2 (SDI-2; Spector et al., 1996); The Sexual Desire and Erotic Fantasies questionnaire (SDEF; Nimbi et al., 2023, 2025)	No further results to report	0.86
Perez and Pepping (2024)	Europe and North America	Convenience sampling	Gender identity	Sexual fetishization, victimization, sexual and relationship satisfaction	**Sexual Fetishization:** “In your experience have you ever felt fetishized?” yes/no (from Anzani et al., 2021) with follow up questions about where fetishization was experienced (e.g., social media, dating apps) and following four questions: (1) “I am seen as a sexual object because I am TNB”; (2) “Dating partners are only interested in me because I am TNB”; (3) “Sexual partners are only interested in me because I am TNB”; (4) “I have felt objectified or fetishized because I am TNB” with response on 1–5 Likert. **Relationship Satisfaction:** four-item version of the Couples Satisfaction Index (CSI-4; Funk & Rogge, 2007). **Sexual Satisfaction:** modified Sexual Satisfaction Scale (Fisher et al., 2015; Mark et al., 2014) removing references to number of partners	**Sexual fetishization:** Women were significantly more likely to have experienced fetishization on social media, having been seen as a sexual object, having felt objectified and fetishized, and having partners interested in them only due to being trans compared to non-binary people. No differences between non-binary people and men. **Sexual and relationship satisfaction:** No differences in relation to gender identity	0.95
Pletta et al. (2022)	USA	Convenience sampling (patients of Community Health Center in Boston, Massachusetts.)	Demographic characteristics and reported relationships characteristics (age, racial identity, gender identity, number of partners, GI of partners etc.)	Sexual behavior with a partner of unknown STI/ HIV status in the past 12 months	**Sexual behavior—other:** number of sexual partners in the past year/engagement in casual sex. **Sexual behavior—partner HIV status:** engagement in sexual behavior with a partner of unknown STI/HIV status in the past 12 months (yes/no). **Sexual partners’ gender identities**: coded as: cisgender female; cisgender male; transfeminine (transgender female/male-to-female/MtF, or non-binary/genderqueer and assigned-male-at-birth/AMAB); or transmasculine (transgender male/ female-to-male/FtM, or non-binary/genderqueer and assigned-female-at-birth/AFAB)	**Sexual behavior—partner HIV status:** being non-binary was not significantly associated with probability of sexual behavior with a partner of unknown STI/HIV status in the past year. **Other information:** not presented divided by gender identity	1.00
Reisner et al., 2023	USA	Data from TransPop (U.S. national probability sample of transgender adults)	Gender identity	Sexual Orientation	**Sexual identity:** "Which of the following best describes your current sexual orientation?" (straight/ heterosexual, lesbian, gay, bisexual, queer, same-gender loving, and other). **Sexual behavior:** Sexual partners in last 5 years (women, non-transgender; men, non-transgender; transgender women, transgender men, and “I have not had sex with anyone in the last 5 years.” Multiple response options were allowed). **Sexual attraction:** How sexually attracted participants were to the following types of people (women, non-transgender; men, non-transgender; transgender women; transgender men; females at birth, genderqueer (AFAB non-binary); males at birth, genderqueer (AMAB non-binary). Multiple response options were allowed. Response options were on a Likert from "not at all" to "very attracted" and included "not sure")	**Sexual identity:** 99.4% of non-binary people identified as a sexual minority, difference with trans men and women was significant (23.3% of trans women, 28.3% of trans men and only 0.6% of non-binary people identifies as straight/heterosexual). **Sexual behavior**: In the past 5 years, 30% did not have sex; 56.0% had sex with cisgender women, 39.1% with cisgender men, 21.8% with trans women, 28.9% with trans men. 37.0% of non-binary people had one sexual partner gender and 33.2% reported more than 3 sexual partner genders. **Sexual attraction:** 78% of non-binary people was sexually attracted to cis women, 52.4% to cis men, 72.3% to trans women, 69.4% to trans men, 76.1% to AFAB non-binary and 63.6% to AMAB non-binary. 77.8% of non-binary participants reported attraction to more than 3 genders	0.82
Reisner et al. (2020)	USA	Convenience sampling (patients accessing clinic cervical cancer screening)	Access to gender-affirming medical care, participant characteristics (including gender identity, and sexual orientation), sexual partner characteristics (currently have a sexual partner, number and gender of sexual partners in the past 12 months), protective barrier use during high-risk sexual acts in the past 12 months, psychosocial context (psychological conditions, lifetime history of sexual abuse/assault, and substance use, tobacco use)	Sexual functioning	**Sexual functioning:** Transmasculine Sexual Functioning Index (TM-SFI)—adaptation Female Sexual Function Index (FSFI), which was piloted and for which exploratory factor analysis, reliability analysis were performed	**Sexual functioning:** Gender identity was not a significant predictor	0.86
Rosenberg et al. (2021)	Australia	Sample was part of Australian Trans & Gender Diverse Sexual Health Survey study (recruitment through online and offline approaches)	Experiences in sexual health	HIV/STI testing	**Sexual behavior in past 12 months:** questions on number of sexual partners, participation in group sex, engaging in sex work or exchange sex, inconsistent condom use with casual partners for vaginal/front hole or anal sex. **Sexual health access:** general practitioner, clinics, community-based services, hospital-based. **Gender insensitivity of Sexual health care settings:** Within each health setting, responses to each item (no previous experience or some previous experience) were summed, accounting for reverse-coded items	**Sexual health care access:** AFAB non-binary people were more likely to have accessed sexual health care through a GP (91.0%; *p* < .001). AMAB non-binary participants were more likely to have used community-based services (28.9%; *p* = .003). No differences in access to hospital-based sexual health care was reported (*p* = .585). AFAB non-binary participants reported highest scores of gender insensitivity in sexual health care (M = 2.28, SD = 1.20), both AMAB and AFAB non-binary people found hospitals to be most gender insensitive, followed by GPs. **Sexual behavior:** not divided by gender identity	0.91
Rothblum et al. (2020)	USA	Participants of larger longitudinal study (Generations study)	Asexual identity	Gender identity, sexual attraction and behavior, outness to others, felt stigma, and everyday discrimination, experience of LGBT community	**Sexual identity:** “Do you consider yourself to be…” (lesbian, gay, bisexual, queer, same-gender loving). **Sexual Orientation:** Which of the following best describes your current sexual orientation” with choices of straight/heterosexual, lesbian, gay, bisexual, queer, same-gender loving, or other (write-in). **Sexual behavior:** “In the last 5 years, who did you have sex with? By sex, we mean any activity you personally define as sexual activity. Please mark all that apply.” (cisgender women, cisgender men, transgender women, and/or transgender men, or not at all—yes/no for each). **Sexual attraction:** question on sexual attraction to non-trans women, non-trans men, trans women, and/or trans men (somewhat or very, not at all, not very, or not sure)	**Asexual sexual orientation and gender identity (GI)**: Nearly three quarters (72.26%) of asexual participants identified as non-binary compared to 6.35% of non-asexual participants. **Other sexual variables:** No separated analysis for gender identity, as focus was asexual identity	0.95
Rutherford et al. (2021)	Canada	Data from Sex Now 2018 survey—Convenience sample (community organizations led in-person recruitment at Pride festivals and related events across 15 Canadian cities, online promotion prior to events)	Gender identity	Mental health and substance use, body image, health resources/ have gone to health professional, sexual Health (STI prevalence, testing, HIV testing, PrEP, vaccination), Healthcare access	**Sexual Orientation identity:** able to select more than one sexual identity (i.e., selections were not mutually exclusive). **Sexual health:** not specified, but link to full questionnaire is available in article	**Sexual orientation identity:** Non-binary people were less likely to identify as gay (32.7% vs. 85.2%), but significantly more likely to identify as queer (49.3% vs. 6.6%), pansexual (32.0% vs. 2.8%), bisexual (17.3% vs. 10.4%), asexual (4.7% vs. 0.6%), and other (2.7% vs. 0.4%) compared to cis people and as likely to identify as straight (0.7% and 0.6%). **Sexual Health related variables:** Being denied access – Even after controlling for confounders, trans and non-binary people were more likely to have been denies health services compared to cisgender participants (68.0% of non-binary participants and 84.2% of cisgender participants)	0.95
Smalley et al. (2016)	USA	Convenience sampling (online, snowball)	Gender identity, Sexual orientation	Health risk behaviors—the only relevant one for this review is sexual risk (Have unprotected sex, Have sex under the influence)	Health risks (including sexual health risks—Have unprotected sex, Have sex under the influence)—Health Risk Questionnaire (HRQ)	**Have unprotected sex**: 41.4% of non-binary people reported having had unprotected sex. There was no significant difference between non-binary people and other genders. **Having sex under the influence**: 8.0.% of non-binary participants reported sex under the influence. No significant difference with other genders	0.73
Walters et al. (2023)	USA	Convenience (sampling through universities)	Changes in sexual behavior due to covid, social support from partner, cohabitation, demographics, depression	Relationship satisfaction	Relationship Assessment Scale (RAS; Hendrick, 1988)	**Relationship satisfaction:** Gender identity was not significantly associated with relationship satisfaction	0.91
Winer et al. (2024)	USA, Canada, UK	Convenience/snowball sampling (quantitative data from the online 2018 Asexual Community Survey – ACS)	Demographics	Patterns of identity in the asexual community	Not specified in paper	**Patterns of identity among asexual people:** asexual participants that had a history of identifying as bisexual or pansexual were more likely to be non-binary	0.73

### Design and Characteristics of Included Studies

All 44 included papers used a cross-sectional design. Most (34/44) used convenience and/or snowball sampling and recruited their participants online (e.g., social media ads, listservs) and via word of mouth and community LGBT organizations (e.g., Holt et al., 2023; Katz-Wise et al., 2016; Smalley et al., 2016). A large proportion (21/44) reported using data from, or subsamples of participants recruited for, larger studies (e.g., Anzani & Prunas, 2020; Dargie et al., 2014). Two papers (Kattari et al., 2019, 2021) used data from the Healthy Kids Colorado Survey (HKCS; Colorado Department of Public Health and Environment, n.d.), which includes a representative sample of US high school students, and three studies (Bishop et al., 2023; Goldberg et al., 2020; Rothblum et al., 2020) analyzed data from The Generations Study (Krueger et al., 2020), a longitudinal cohort study of LGBT adults in the United States. Lastly, two studies (Koós et al., 2024; Lin et al., 2024) used data from the International Sex Survey (Bőthe et al., 2021).

### Participant Characteristics

The number of non-binary participants included in each study varied widely, ranging from 4 (Dargie et al., 2014) to 2783 (Lin et al., 2024). Most studies (27/44) included fewer than 100 non-binary individuals (of which 10 included fewer than 50). In most, demographics such as age, race/ethnicity or education were not reported separately for non-binary participants. Where this information was available (14/44 for age, 9/44 for race/ethnicity and 11/44 for education), non-binary people were mostly from white backgrounds (e.g., Hibbert et al., 2020; Littman et al., 2024), below the age of 35 (e.g., Kennis et al., 2022a), and university or college educated (e.g., Almås et al., 2024; Kennis et al., 2022b; Mark et al., 2018). Table 3 provides full sample characteristics in relation to these variables.

**Table 3:** Participant/sample characteristics

Citation	N total sample (N non-binary participants)	Mean age sample (Mean age non-binary participants	Race/ ethnicity total sample ( race/ ethnicity non-binary participants)	Non-binary people Socioeconomic status	Non-binary people education	Gender identity labels	Trans identity of the non-binary sample	Sexual orientation identity (descriptive statistics for non-binary participants)
Almås et al. (2024)	334 (93)	62.7% was under 30 (62.5% was below 40)	Not reported (Not reported)	Not reported	60.3% completed higher education	“Female gender identity orientation” (FGIO) 93; “male gender identity orientation” (MGIO) 148; “nonbinary gender identity orientation” (NBGIO) 93	Not specified	NA
Anzani and Prunas (2020)	296 (55)	26.88 (Not reported, but not significantly different from cis participants)	Not reported (Not reported)	Not reported	Not reported	Cisgender men 85; Cisgender women 156; "Viewed gender as a nonbinary construct" (most common labels reported were non-binary and genderqueer) 55	Participants whose gender assigned at birth was not aligned with their experienced or expressed gender and who adhered to a non-binary view of gender were assigned to the non-binary group. No trans binary people in the study	NA
Atkins et al. (2024)	14,687 (65)	No history of transactional sex: 31.5;Transactional sex in last 5 years: 32.6 (Not reported)	52% Black; 47% White (Not reported)	Not reported	Not reported	Cisgender woman 6726; Cisgender man 7690; Transgender woman 41; Transgender man 72; Gender nonconforming or other 65	Not specified	NA
Bishop et al. (2023)	1492 (94)	No mean reported. 62% between 18 and 25 (not reported)	62% White, 21% Latino, 16% Black (Not reported)	Not reported	Not reported	Woman 737; Man 661; Non-binary/ genderqueer 94	Screening question "Do you, personally, identify as lesbian, gay, bisexual, or transgender?” (if yes to transgender, signposted to participate to sibling study. Non-binary people were still included)	No separate data
Boskey and Ganor (2022)	167 (21)	No mean reported. Majority of participants were 15–18, followed by 18–21 at the time of first consultation (Majority between 18 and 21, followed by 21–24)	Not reported, no significant difference between binary and non-binary participants (81% White non-binary sample/ 95% non-Hispanic)	Not reported	Not reported	Binary trans men 146; Non-binary transmasculine 21 (Non-binary 76%, Genderqueer 14%, Other 10%)	Participants are "transmasculine" group of patients (treatments seeking)	Heterosexual 0%; Homosexual 24%; Bisexual 5%; Pansexual 19%; Queer 29%; Asexual 10%; Demisexual 10%; Other 0%; Choose not to answer 5%
Bosse and Chiodo (2017)	175 (34)	21.1 (Not reported)	Caucasian 81.1% (Not reported)	Not reported	Not reported	(cis) men 21.7%; (cis) woman 41,1%; Transgender 1.3%; FTM/ Transmasculine 11.4%; MTF/ Transfeminine; 1.1% Genderqueer 13.7%; Non-binary gender 1.7%; Agender/Do not Use Label 4.0%; Questioning 0.6%; Other 2.3%	No specific question on trans identity for non-binary people	AFAB individuals that identified as Genderqueer (*n* = 21) were asexual (9.5%), bisexual (19%), lesbian (9.5%), pansexual (23.8%) and queer (38.1%). Agender (*n* = 5) were asexual (40%), gay (20%), Pansexual (40%). Non-binary (*n* = 3) were asexual (33.3%), bisexual (33.3%), queer (33.3%). AMAB individuals that identified as genderqueer (*n* = 3) were asexual (33.3%), bisexual (33.3%), pansexual (33.3%)
Burgwal et al. (2019)	853 (230)	Age binary trans people 27.1 (24.7, significant difference with binary participants, *p* = .004)	Not reported, no significant difference between binary and non-binary participants (11.2% of GQNB were ethnic minority)	Non-binary people reported significantly lower economic stress compared to trans binary people	Most people had a "middle" level of education	Trans women 254 (29.8%); Trans men 369 (43.2%), Non-binary people (Non-binary/ Genderqueer/ Gender nonconforming or other with non-binary label) 230 (26%)	No specific question	93.8% GQNB reported being a sexual minority
Byrne et al. (2022)	704 (290)	32.5 (Not reported)	78.0% Pakeha/New Zealand European (White) (Not reported)	Not reported	Not reported	Trans men 185; Trans women 227, non-binary AMAB 72, non-binary AFAB 218	No specific questions reported. Non-binary and trans people are both included	NA
Dahl et al., 2024	1036 (69)	38.51 (Not reported)	87.9% White (Not reported)	Not reported	Not reported	Cisgender Man 404 (39.0%); Cisgender Woman 560 (54.1%); Transgender Woman 16 (1.5%); Transgender Man 20 (1.9%); Agender 4 (0.4%); Genderfluid 10 (1%); Genderqueer 9 (0.9%); Multiple 6 (0.6%); Genderqueer Woman 2 (0.2%); Other (did not specify) 5 (0.5%) Non-binary identities were combined and labelled “Other gender minority (Agender; Genderfluid; Genderqueer; Multiple; Genderqueer Woman; Other)”	No question specified	No separate data
Dargie et al. (2014)	Descriptive analysis: 64 (4); Inferential analysis: 36 trans people and 689 cisgender people as control	32.77 (Not reported) for descriptive analysis sample	Not reported (Not reported)	Not reported	Not reported	Trans women 27; Trans men 32; Other (genderqueer or cross-dresser) 4; Declined response 1	No question specified	No one in the "other" group identified with heterosexual, half had low identification (did not identify) with using no label (the other half did not respond) and around 80% highly identified with the label queer
Dubin et al. (2021)	821 (62)	No mean reported. 55.73% of sample between 18 and 34 (Not reported)	58.20% White, 16.40% Other race (Not reported)	Not reported	Not reported	Male identified (trans male and male) 292; Female identified (trans female and female) 212; Non-binary and other (referred to it as “non-binary”) 62; Choose not to disclose 249	No specific question on trans identity	**Non-binary and other**: 62.33% input a response for SO. **AFAB**—Most common response was Something Else 41.67%, followed by Lesbian (input) 19.44%, Don’t know 13.89%, Homosexual/gay 13.89%, Bisexual 11.11% while Choose not to disclose and Heterosexual/straight were 0%. **AMAB**—Homosexual/gay 100%
Eliason and Streed (2017)	277 (15)	37.8 (Not reported)	White 80% (Not reported)	Not reported	Not reported	(cis) Female 103; (cis) Male 85; Genderqueer/gender variant 15; Transgender 8	No specific question	47% of genderqueer/gender variant participants indicated that their sexual identity was ‘‘something else’’ on the first part of the NHIS question. 7% of genderqueer/gender variant identified as Bisexual and 46% as gay/lesbian. For part 2 of the question Genderqueer/gender variant that responded "something else" in the first part responded "Use other labels" (43%) or "Something else" (57%). Transgender and genderqueer/gender variant individuals accounted for the majority of those who chose to report their sexual identity as ‘‘something else’’ (and they often reported ‘‘something else’’ on both parts of the questions)
Fisher et al. (2018)	228 (32)	17.86 (17.25)	White 87.7% (Not reported)	No differences reported between trans binary and non-binary youth	No differences reported between trans binary and non-binary youth	Transmasculine 103; Transfeminine 93; and Gender non-binary 32 All non-binary labels come from open-ended question. Examples given are: gender non-binary, agender, bigender, and gender fluid	All non-binary people were also trans as they identified as "transgender in another way"	Pansexual 21 (65.6%); Queer 23 (71.9%); Bisexual 8 (25.0%); Gay 10 (31.3%); Asexual 6 (18.8%); Questioning/unsure 3 (9.4%); Lesbian 6 (18.8%); Heterosexual 1 (3.1%); Do not wish to answer 0 (0.0%)
Fuller and Riggs (2021)	345 (87)	27 (Not reported)	White, not of Hispanic origin 75.7% (Not reported)	Not reported	Not reported	Male109; Female 85; non-binary 87; another gender (non-cis) 45; agender 19	Inclusion criteria was to be transgender	No separate data
Galupo et al. (2018)	363 (51)	26.5 (Not reported)	White/Caucasian 77.7% (Not reported)	Not reported	Not reported	Cisgender people 278; Trans people 85 (of which 51 non-binary people)	Non-binary labels/people appear to be coming out of transgender group	Lesbian/gay 13 (61% of total transgender people identifying as lesbian); Bisexual 5 (62.5% of total transgender people identifying as bisexual); Pansexual/queer 33 (58.9% of total transgender people identifying as pansexual/queer)
Goldbach et al. (2023)	169 (66)	Mean not reported. Majority under 34 (Not reported)	79.3% White (Not reported)	Not reported	Not reported	Transfeminine identity 60.9% (103), Non-binary (some examples: genderqueer, two spirits) 31.4% (*n* = 53); Agender 7.7% (*n* = 13)	Not directly assessed	No separate data
Goldberg et al. (2020)	1507 (94)	No total reported, divided by sexual identity. Queer participants 26.1 (0.8) were significantly younger rest of sample (Lesbian/gay: 35.4 (0.6); Bisexual 26.8 (0.5); Other 27.8 (1.2))	Majority from all sexual identity sample was white, e.g., between 55.3% for queer and 65.1% for bisexual (Not reported)	Not reported	Not reported	Cisgender men 672; Genderqueer/ non-binary 94; Cisgender women 741	Screening question on LGBT identity, with binary trans people then reassigned to sister study Trans pop. No direct question on trans identity reported	Queer 24.9%; Gay 14.9%; Bisexual 19.5%; Other 39.8%
Hibbert et al. (2020)	3507 (244)	27.1 (No mean reported, most common age band was 18–24 (50%) or 24–34 (34%))	95% White for total trans sample (93% White non-binary sample)	Majority were in full-time employment (33%) or a student (29%)	50% had a university level education or higher	Trans men 147; Trans women 88; Non-binary 244; In another way 21; Cisgender 3007	Follow-up question on trans identification, used to separate binary people in trans and cis. No specific information reported on trans identification for non-binary subgroup	Gay/lesbian/homosexual 23%; Bisexual 16%; Straight/heterosexual 2%; Queer 33%; Asexual 8%; In another way 18% (inferential analysis were done grouping trans people and comparing with cis)
Holt et al. (2023)	1613 (863)	No mean reported. Median age 27 (No mean reported. Median age 26)	Aboriginal or Torres Strait Islander 4.4% of total sample (Aboriginal or Torres Strait Islander 3.8% of non-binary people)	68.8% annual income below 40 k	51.8% university educated	Trans men 353 (21.9%); Trans women 397 (24.6%); Non-binary (e.g., agender, genderqueer) 863 (53.5%)	Inclusion criteria was to have gender identity different than assigned at birth, non-binary identity was given priority if both binary and non-binary identities expressed. No further questions on trans identity for non-binary people	Asexual 87 (10.1%); Bisexual 179 (20.7%); Fluid 197 (22.8%); Heterosexual 12 (1.4%); Homosexual 117 (13.6%); Queer 264 (30.6%)
Jacobson and Joel (2019)	6104 (744)	Not reported (Not reported)	Not reported (Not reported)	Not reported	Not reported	Transgender (people that identified as transgender, transman, transwoman) 406, gender-diverse (people that identified as genderqueer or other) 744 and cisgender 4954	Separation between genderqueer/non-binary and trans people	NA
Jann et al. (2022)	14,372 (232)	Mean not reported. More than half over 30, but 64.4% of TGNC under 30 (Mean not reported. Majority 29 or below)	39.5% white, 30.7% Hispanic/Latinx (Not reported)	8.6% reported being homeless, which was similar to trans men, less than trans women (around 19%) and more than cisgender people (around 2–3%) [not statistically tested, only trans vs cis]	NA	Cisgender gay/homosexual men 57.5%; Straight/heterosexual men 8.9%; Straight/heterosexual women 5.2%; Bisexual women 3.5%; TGNC people 3.2% (Genderqueer/ non-binary 50.7%; Transgender women/transfeminine 38.2%; Transgender men/transmasculine 11.1%)	Specific information not reported	Gay/homosexual 47 (20.3%); Lesbian 3 (1.3%); Bisexual 22 (9.5%); Heterosexual 2 (0.9%); Other 29 (12.5%); Pansexual 30 (12.9%); Queer 69 (29.7%); Questioning 4 (1.7%); Unknown/unreported/declined 26 (11.2%)
Kattari et al. (2021)	4729 (81)	Students were 14 + , mean not reported (middle and high school students), mean not calculated but above 16 (Not reported)	57.31% White (Not reported)	Not reported	Not reported	Not transgender 12,467; Don't Know transgender 175; Transgender girl/woman 57; Transgender boy/man 110; Transgender other 81	All non-binary people were also trans as they identified as "transgender in another way"	No separate data
Kattari et al. (2019)	4834 (57)	16.2 (Not reported)	53.8% White; Latino/Hispanic 26.75% (Not reported)	Not reported	Not reported	Don’t Know 69; Not Transgender 4611; Transgender Female 42; Transgender Male 56; Transgender Other 57	All non-binary people were also trans as they identified as "transgender in another way"	No separate data
Katz-Wise et al. (2023)	4087 (167)	89.7% of sample was between 18 and 25 (Not reported)	47.8% White; 20.1% Other ethnicities; 16.5% Black (Not reported)	Not reported	Not reported	Cisgender girl/woman 2370 (58%); Cisgender boy/man 1497 (36.6%); Transgender girl/women 6 (0.2%); Transgender boy/man 47 (2.2%); Non-binary person 167 (4.1%)	No specific question reported	No separate data
Katz-Wise et al. (2016)	452 (185)	32.6 (Not reported)	White Non-Hispanic 79.4% (Not reported)	Not reported	Not reported	Transfeminine 36.9%; Transmasculine 63.1% (of whom non-binary 40.9%) Some of the non-binary label used were: genderqueer, gender variant, gender nonconforming, other	No explicit question to check if non-binary people are also trans. Study was open to trans and gender-diverse individuals	No separate data
Kennis et al. (2022b)	480 (78)	30.21 (30.55)	Not reported (Not reported)	Majority was employed (43.59%) or a student (35.9%), nonetheless non-binary people were more likely to be unemployed when compare to cisgender people (20.51% vs. 9.8%), with more similar scores to binary trans people (18.78%)	Majority had college/university education (74.36%). No significant difference with binary trans and cis participants	Transgender men 125; Transgender women 72; Non-Binary individuals 78; Cisgender men 98; Cisgender women 107 Non-Binary selected this label or used the text-box response option that indicating a non-binary identity (e.g., "non-binary transmen")	Specific separate question on trans identity (79.5% of non-binary participants indicated having a transgender identity)	NA
Kennis et al. (2022a)	259 (62)	30.4 (28.32)	Not reported (Not reported)	no difference between binary and non-binary trans people in relation to housing	no difference between binary and non-binary trans people in relation to education (majority were highly educated)	Transgender men 125; Transgender women 72; Non-binary transgender 62	Specific separate question on trans identity (of the non-binary and/or other identity group, 18 participants did not identify as transgender, and they were excluded from sample.)	NA
Koós et al. (2024)	75,117 (2612)	32.07 (Not reported)	Not reported (Not reported)	Not reported	Not reported	Men 31,454 (41.9%); Women 41,016 (54.6%); Genderdiverse 2612 (3.5%)	Unclear. From supplemental table S1 looks like non-binary people might be both trans and not trans	No separate data
Lin et al. (2024)	82,243 (2783)	32.39 (Not reported)	Not reported (Not reported)	Not reported	Not reported	Men 32,549 (39.6%); Women 46,874 (57.0%); Non-binary individuals 2315 (2.8%); Individuals with other gender identities 468 (0.6%)	No specific information about trans identification	No separate data
Littman et al. (2024)	6653 (1013)	Trans men: majority below 39; Trans women: Majority below 49 (Majority was below 39)	Trans men: 59.2% White, 18.4% Black; Trans women: 77.6% White, 7.5% Black (66.5% White; 10.1% Black)	Majority ≤ 50,000	Majority had completed “Some college or technical school” or below	Trans men 25%; Trans women 59%; Non-binary 16%	Unclear, no specific question reported, but participants were screened for participation based to the following questions: 1) “Are you transgender, trans, non-binary or gender diverse?” (yes/no) 2) “Is your current gender identity different than the sex on your original birth certificate?” (yes/no)	No separate data
Mark et al. (2018)	955 (57)	33 (33)	White 87.4% (White 87.7%)		Majority had completed college/university (28.1%) or had completed some college (22.8%)	Cisgender women 605 (63.4%); cisgender men293 (30.7%); genderqueer 57 (6.0%)	No specific information about trans identification	Straight 7 (12.3%); Gay 9 (15.8%); Lesbian 0 (0%); Bisexual 20 (35.1%)
McKenna et al. (2021)	251 (61)	24.0 (23.33)	70.5% White (68.9% White)	Not reported	Not reported	Cisgender women 39.4% (99); Cisgender men 36.3% (91); Non-binary 24.3% (61)	Non-binary people are included in this study, but binary transgender people are not, which could mean non-binary people in this study were not trans, but could also mean that binary trans people were excluded while non-binary people's trans identity was not assessed	NA
Nadarzynski et al. (2023)	1429 (28)	36.6 (Not reported)	85% White (Not reported)	Not reported	Not reported	Male (including trans male) 1386 (97%); Female (including trans female) 6 (< 1%); Non-binary 28 (2%); Other 9 (< 1%)	No specific question reported	NA
Nimbi et al. (2024)	1041 (153)	25.25 (Not reported)	Not reported (Not reported)	Not reported	Not reported	Female 718 (68.97%); Male 81 (7.78%); Questioning 89 (8.54%); Non-binary 153 (14.69%)	No specific question reported	Authors highlight high percentage of non-binary people in the sample. Non-binary people in the sample mainly identified as asexual (22.89%) or gray-asexual (21.77%)
Pletta et al. (2022)	141 (27)	27.4 (Not reported)	75.2% White/ 88.7% non-Hispanic (Not reported)	Not reported	Not reported	Transmasculine spectrum: Non-binary gender (e.g., non-binary bigender, genderqueer) identity (no 114, yes 27)	Paper is on "transmasculine" group of patients (treatments seeking)	NA
Perez and Pepping (2024)	316 (239)	26.84 (Not reported)	80% White (Not reported)	Not reported	Not reported	Men 39 (12.3%); Women 38 (12%); Non-binary and/or gender diverse 239 (75.6%)	No specific question reported	No separate data
Reisner et al., 2023	274 trans binary and 1162 cis participants (76)	34.2 trans binary sample; 48.5 cis sample (30.4)	Trans binary sample: 56.5% White and 43.5% People of color; Cis sample: 72.3% White and 27.7% People of color (54.2% White; 21.7 Latinx)	Not reported	58.7% had completed at least some college	274 Trans people (Trans man 30.9%, trans woman 30.8%, trans non-binary 31.3%) and 1162 Cisgender people	All non-binary participants were also trans (follow up question on gender identity—see method of asking about gender identity)	Queer 35%; Pansexual 15%; Lesbian 13.2%; Bisexual 12.9%; Asexual 11.9%
Reisner et al. (2020)	150 (30)	27.5 (Not reported)	74.7% White (Not reported)	Not reported	Not reported	Transgender men (FtM) 50.4%; Man/male 28.7%; Genderqueer/ non-binary 20.0%; Another gender 3.3%	Paper is on "transmasculine" group of patients of cervical screening	No separate data
Rosenberg et al. (2021)	1613 (1067)	30.7 (Not reported)	4.3% Aboriginal and/or Torres Strait Islander (Not reported)	Not reported	Not reported	Man/trans man 258 (16.0%); Woman/trans women 288 (17.9%); Non-binary, presumed male at birth 340 (21.1%); Non-binary, presumed female at birth 727 (45.0%) Participants reported 58 different gender labels, which were recoded as trans men/men, trans women/women, gender non-binary (AFAB/AMAB) Those who reported both binary and non-binary gender labels were categorized as non-binary	Participants could be trans and/or gender-diverse (no other item to assess trans identity)	NA
Rothblum et al. (2020)	19 asexual people compared with 1504 non-asexual LGB people (72.26% of asexual subsample)	No mean reported. Of asexual respondents (91.19%, SE = 5.47) were 18–27 (Not reported)	80.46% White for asexual subsample (Not reported)	Not reported	Not reported	Asexual people were: Women 27.74%; men 0%; Genderqueer and non-binary (GQNB) 72.6% Non-asexual (LGB sample) were: Women 54.92%; Men38.74%; Genderqueer and non-binary (GQNB) 6.35%	Stated in discussion that this study is about asexual individuals who are LGB or GQNB sexual minorities but do not identify as trans or straight	No separate data
Rutherford et al. (2021)	3423 (150)	No mean reported. Majority below 29 (No mean reported. 40.7% were below 25 (66.0% of non-binary responders were below 30). Both trans and non-binary groups were younger than the cisgender group.)	73.6% White (70.0% White)	Majority reported having enough to make ends meet with no extra (35%) or with extra money to spend (20.7%), however 20.7% reports being unable to make ends meet. Non-binary people were overall twice as likely to reporting having to cut back and four times as likely to report not making ends meet than cisgender people	Non-binary participants were less likely to have a Bachelor’s degree (24.0% vs. 32.2%) or above a Bachelor’s degree (8.0% vs. 22.1%), completing post-secondary school (28.7% vs. 25.1%) compared to cis people, but this was not significant after adjusting for confounders	Cisgender participants 3083; Trans participants 296, Non-binary participants 150 (106 people in both trans and non-binary subsample as they identified as both) People that gave alternative label selecting option “neither. I prefer to self-describe as: _____________.” to gender question (any non-binary label included., e.g., "enby" or "genderqueer")	Specific separate question on trans identity/experiences Trans and non-binary group are not mutually exclusive (non-binary group only has non-binary participants, with or without trans identity, while the trans group is mix of trans non-binary and binary people 106 participants identified both as non-binary and as trans)	Gay 32.7%; Asexual 4.7%; Straight 0.7%; Bisexual 17.3%; Pansexual 32.0%; Queer 49.3%; Heteroflexible 1.3%; Other 2.7%
Smalley et al. (2016)	3279 (117)	29.8 (Not reported)	Caucasian 68.9% (Not reported)	Not reported	Not reported	Female 2038; Male 916; Transgender female 82; Transgender male 126; Genderqueer, non-binary, or other 117	No specific question	Most genderqueer or non-binary participants identified as queer or pansexual – omnisexual (50.0% and 24.1%). 10.7% was Bisexual
Walters et al. (2023)	175 (18%)	20.5 (Not reported)	63% White; 21% Hispanic/Latinx (Not reported)	Not reported	Not reported	Women 53%; Men 14%; Non-binary 18%; Transgender men 5%; Transgender women 4%; Genderqueer 1%; Other 5%	No specific question	No separate data
Winer et al. (2024)	7568 (1926)	24.5 (Not reported)	78.9% White (Not reported)	Not reported	Not reported	Women 4687 (62%); Men 955 (13%); Neither men nor women 1926 (26%)	Transgender identity for whole sample was assessed (transgender identifying (18%); Unsure (8%)), however no specific data reported for non-binary people	No separate data

### Gender Identity Assessment

Of the papers that specified the method or items used to assess gender (30/44), 18/30 included a question assessing sex assigned at birth (SAAB) and then asked participants to report their gender identity label in different formats, often through selecting one from a range of provided options (e.g., Katz-Wise et al., 2016); 20 papers explicitly stated offering an “other” option and/or a way for participants to add in their own label if it was not already listed (e.g., Atkins et al., 2024; Jacobson & Joel, 2019). Two papers (Hibbert et al., 2020; Rutherford et al., 2021) asked participants a specific question about whether they identified as trans, without enquiring about participants’ SAAB. One paper used an open response format, and answers were subsequently coded and classified by experts (identified as researchers, clinicians, and members of the trans community) in “female gender identity orientation,” “male gender identity orientation,” and “nonbinary gender identity orientation” (Almås et al., 2024). It should be noted that papers which did not specify their methodology still reported data on the gender identity of participants.

### Non-binary Labels in Sexual Research

Overall, many studies (21/44) included one or more non-binary gender labels as options in questions on gender identity, with common options being “genderqueer” and “non-binary” (e.g., Bosse & Chiodo, 2017; Burgwal et al., 2019). As noted above, others relied on participants to select “other” and then specify their preferred label in a text box (e.g., Fisher et al., 2018). One paper presented a specific question on the perception of participants’ gender as a binary or non-binary construct to differentiate between binary and non-binary participants (Anzani & Prunas, 2020). In another study (Holt et al., 2023), it was noted that if participants endorsed both a binary and non-binary gender identity, the non-binary label was given priority when classifying and grouping participants.

Even when separate non-binary labels were provided or reported, they were generally combined to form a single category/subgroup for analysis (e.g., Holt et al., 2023). In some cases, more typical non-binary labels were combined with other gender labels such as “cross dresser” (Anzani & Prunas, 2020; Dargie et al., 2014), “woman but only genetically-wise” (Anzani & Prunas, 2020), or “other” (Dubin et al., 2021). Multiple papers used an ‘other’ category to capture any participants who did not neatly fit into a binary categorization of gender (e.g., Dargie et al., 2014; Kattari et al., 2021).

### Trans Identification

In the majority of the studies (26/44), whether non-binary participants also identified as transgender was not clear, mostly due to a lack of reporting of prompts or items used to assess gender identity (e.g., Jann et al., 2022). Of the remaining articles, twelve only included trans non-binary people (with being transgender often being one of the inclusion criteria to participate in the study; e.g., Fuller & Riggs, 2021; Kattari et al., 2019), three only included non-binary people that did not identify as trans (e.g., Rothblum et al., 2020), and three included non-binary people regardless of their trans identification (e.g., Rutherford et al., 2021). Overall, trans identity was mostly not assessed (e.g., Dubin et al., 2021; Katz-Wise et al., 2016), and this information had to be inferred by examining the inclusion criteria for participation and the gender identity items reported within the papers.

### Scales Used to Assess Sexual Variables

#### Sexual Attraction and Behavior

Twelve studies included a measure of sexual attraction. Most measured attraction using a modified Kinsey scale (Kinsey et al., 1953, 2003), for instance measuring attraction from “men/masculinity” to “women/femininity” (Anzani & Prunas, 2020). Other papers assessed attraction toward different genders, often focusing only on attraction toward men and women (Jacobson & Joel, 2019; Kennis et al., 2022a, 2022b). Two studies differentiated between attraction to cisgender and transgender binary individuals using 5-point Likert scales (Goldberg et al., 2020; Rothblum et al., 2020). In relation to attraction toward non-binary genders, one paper measured attraction toward non-binary people separately (although items to assess this were not reported in full; Boskey & Ganor, 2022), one paper gave the option to express distinct levels of attraction toward AFAB and AMAB non-binary people (Reisner et al., 2023), and one paper gave participants the option to select from a list the genders to which they were attracted, with response options: trans men, cis men, trans women, cis women, genderqueer or nonbinary people, none of the above, others (Byrne et al., 2022).

Eighteen papers included measures of sexual behavior. Two papers mentioned specific sexual acts including vaginal/ “front hole” or anal intercourse (Rosenberg et al., 2021) and “receptive (either anal or vaginal)” or “insertive (either with a penis or a strap-on)” intercourse (Goldbach et al., 2023).

Seven papers asked about gender of sexual partners. Two studies did not give an option for reporting non-binary sexual partners (Fisher et al., 2018; Reisner et al., 2023), two studies gave a non-binary partner option but the exact prompt was not reported (Boskey & Ganor, 2022; Pletta et al., 2022), and one paper gave options for non-binary or transgender partners, but for analysis, the two categories were paired together into a “transgender/genderqueer or non-binary partners” category (Goldberg et al., 2020).

#### Sexual Orientation Identity and Sexual Fluidity

Thirty-three papers reported on sexual orientation identity, although 16 did not report separate results for non-binary subgroups (e.g., Katz-Wise et al., 2016). While two papers provided participants with only a few alternatives in relation to labeling their sexuality, such as heterosexual (straight), gay or lesbian, bisexual, and not sure (Kattari et al., 2019, 2021), others were more inclusive, either by allowing participants to select an “other” option and enabling participants to provide their own label (e.g., Rutherford et al., 2021), or by expanding the range of options provided (i.e., pansexual, queer, asexual, and other labels), or both (e.g., Reisner et al., 2023). Some papers also allowed participants to select multiple labels (e.g., Fisher et al., 2018). One paper used the Sexuality Questionnaire (SQ; Alderson, 2012) and prompted participants to indicate how much they associated with multiple identity labels, including heterosexual, bisexual, gay or lesbian, queer, transexual, no label, or other (Dargie et al., 2014). Only two studies looked at sexual fluidity. One study (Katz-Wise et al., 2016) used a single and gender-neutral item, while a more recent study (Katz-Wise et al., 2023) used multiple gender-neutral questions that looked at attraction and identity changes.

#### Sexual Satisfaction

Six papers included measures of sexual satisfaction. Three papers (Kennis et al., 2022a, 2022b; Mark et al., 2018) used the Global Measure of Sexual Satisfaction (GMSEX; Lawrance & Byers, 1995). One paper (Holt et al., 2023) used a single Likert scale item from the Multi-Dimensional Sexual Self-Concept Questionnaire (Snell, 2010). One paper used a modified version of the Sexual Satisfaction Scale (Fisher et al., 2015; Mark et al., 2014) removing indications on the number of partners (Perez & Pepping, 2024) and one did not report the measure used in detail (Almås et al., 2024).

#### Relationship Satisfaction

Six studies considered relationship or romantic satisfaction and most measures were gender neutral. Specifically, Holt et al. (2023) used a single item (“I am satisfied with the romantic aspects of my life”, 5-point Likert scale), while Mark et al. (2018) used the General Measure of Relationship Satisfaction (GMREL; Lawrance & Byers, 1992). The relationship satisfaction subscale of the Gay and Lesbian Relationship Satisfaction Scale (GLRSS; Belous & Wampler, 2016) was used by Fuller and Riggs (2021). Although this measure was mostly gender neutral, one of the items referenced partners using gendered pronouns (“I often tell my partner that I love him/her”). Dargie et al. (2014) and Walters et al. (2023) used Hendrick’s Relationship Assessment Scale (RAS; Hendrick, 1988). For Dargie et al. (2014), it is unclear whether the measure was completed by non-binary participants. Lastly, Perez and Pepping (2024) used the 4-items version of the Couples Satisfaction Index (CSI-4; Funk & Rogge, 2007).

#### Other Sexual Variables

Other sexual variables were investigated by a very limited number of papers. One study measured sexual assertiveness. McKenna et al. (2021) modified the Sexual Assertiveness Questionnaire (SAQ; Loshek & Terrell, 2015) by changing the language to be gender-neutral to make it inclusive of non-binary participants. The same study also used the Sexual Consent Scale-Revised (SCS-R; Humphreys & Brousseau, 2010) to measure sexual consent, attitudes, beliefs, and behaviors, and performed a confirmation factor analysis which found that the original structure did not fit a cisgender and non-binary sexual minority sample (McKenna et al., 2021).

One study measured sexual fantasies. Anzani and Prunas (2020) used the Italian version of the Sexual Fantasy Questionnaire (SFQ; Bogaert et al., 2015) and performed a confirmatory factor analysis which found that the proposed structure did not fit the data with a sample of non-binary and cisgender adults (and remained inappropriate, even when only cisgender participants were included). One paper looked at BDSM practices and fantasies using two measures that were created for the study (Dahl et al., 2024).

To measure sexual desire, Mark et al. (2018) used the Dyadic Subscale of the Sexual Desire Inventory (SDI-D; Spector et al., 1996). Although this tool uses gender-neutral language overall, one item references biological sex (“Compared to other people of your age and sex, how would you rate your desire to behave sexually with a partner?”; Spector et al., 1996).

Koós et al. (2024) used the gender-neutral Pornography Use Motivations Scale (PUMS; Bőthe et al., 2021) and questions on frequency and duration of porn use. Fetishization experiences were assessed through four gender-neutral items in Perez and Pepping (2024).

One study assessed sexual function. Reisner et al. (2020) developed and piloted the Transmasculine Sexual Functioning Index (TM-SFI) and conducted psychometric assessments with a sample of transmasculine patients accessing gender care in the USA. The TM-SFI was adapted from the 6-item version of the Female Sexual Function Index (FSFI-6; Isidori et al., 2010) to better assess sexual function in transmasculine people who were eligible for cervical cancer screening and had not had genital surgery.

Sexual distress was measured using the Short Sexual Distress Scale (SDS-3; Pâquet et al., 2018) in Lin et al. (2024)’s study. One paper measured masturbation; however, the specific item was not reported in the article (Almås et al., 2024).

The two papers that investigated sexual self-concept discrepancies (defined as discrepancies between the actual/ideal and actual/ought sexual self; Kennis et al., 2022a), used two gender-neutral items created by the researchers, as well as the Transgender-Specific Body Image Worries Scale (T-Worry; Dharma et al., 2019), a validated measure to assess trans-specific body worries in relation to sex (Kennis et al., 2022a, 2022b).

Lastly, one study examined sexual self-esteem. Kennis et al. (2022a) used four subscales of a sexual self-concept questionnaire created by Buzwell and Rosenthal (1996).

### Review of Study Findings

#### Gender Transition

A minority of papers in our sample (8/44) examined gender transition among non-binary participants. Burgwal et al. (2019) found that 75.5% of non-binary people in their general population sample of 853 European trans and gender-diverse individuals, 16 and older, reported desiring gender-affirming medical care. Kennis et al. (2022b) found that compared with binary trans participants, fewer non-binary adults in their sample (mainly recruited through social media) were seeking hormone treatment (27% vs. 81%) and gender-affirming surgeries (20% vs. 51%). In a different paper, it was found that 45% of non-binary people in a sample of trans/non-binary US veterans were receiving gender-affirming care compared to 82% of binary trans people. The same authors found that a minority of non-binary participants had had gender-affirming surgery compared to trans men (27% vs. 64%), while 54.4% of non-binary veterans were not taking hormones compared to 16.6% of trans women and 17.6% of trans men veterans (Littman et al., 2024).

Six papers included inferential analysis and comparison between non-binary and binary individuals in relation to gender-affirming procedures. Overall, non-binary people were less likely than binary trans people to have accessed gender-affirming care or to have received gender-affirming treatments such as surgeries or hormones (Holt et al., 2023; Kennis et al., 2022a; Littman et al., 2024) and were more likely to report no treatment desire (Kennis et al., 2022a).

In their US sample of treatment seeking transmasculine individuals (21 = non-binary, 146 = trans men), Boskey and Ganor (2022) reported that 95% of non-binary participants were currently seeking chest surgery while only one participant (5%) was pursuing genital surgery; this was significantly different than trans men, of whom 73% and 27% were seeking chest and genital surgery, respectively. Rutherford et al. (2021) compared non-binary individuals and cisgender non-heterosexual men having sex with men and found that non-binary participants were more likely to have been denied access to hormone therapy (13.3% vs. 0.3%) and gender-affirming surgeries (9.3% vs. 0.2%).

#### Sexual Orientation Identity and Labels

Most papers reported that non-binary participants primarily identified as queer—between 24.9 and 71.9% (e.g., Bosse & Chiodo, 2017; Dargie et al., 2014; Fisher et al., 2018; Hibbert et al., 2020; Holt et al., 2023; Jann et al., 2022; Rutherford et al., 2021; Smalley et al., 2016). The label ‘bisexual’ was also commonly chosen by non-binary participants in some studies; however, it is unclear if this accurately reflects their orientation or rather if it is due to lack of available alternatives, as when provided, non-binary people also frequently selected “something else” or “another label”, as reported by Dubin et al. (2021) and Eliason and Streed (2017). Although it should also be noted that in the National Health Interview Survey (NHIS) used by Eliason and Streed (2017), gender identity and sexual orientation were conflated in their measure of sexual orientation.

In an US nationally-representative sample of 1507 sexual minority individuals, 25% of non-binary participants identified as queer (significantly more than cis men and cis women) and one third of all queer participants endorsed a non-binary gender identity (Goldberg et al., 2020). Likewise, Fisher et al. (2018) found that nonbinary youth in their sample (aged 14–21) were significantly more likely to identify as queer compared with binary trans young people and Boskey and Ganor (2022) reported that assigned-female-at-birth (AFAB) non-binary people were significantly more likely to identify as queer compared to binary trans men.

Considering sexual minority identity status, Burgwal et al. (2019) found that non-binary participants were significantly more likely to identify as a sexual minority than binary trans participants (93.8% vs. 75.8%, *p* < .001). Similarly, Reisner et al. (2023) found that 99.4% of non-binary people in their US probability sample identified as a sexual minority, compared with 23.3% of trans women and 28.3% of trans men, a statistically significant difference.

Regarding heterosexual identity, multiple studies (e.g., Boskey & Ganor, 2022; Littman et al., 2024) reported that trans binary participants were significantly more likely to identify as heterosexual than non-binary people.

Holt et al. (2023) found that non-binary participants in their Australian study on the sexual and relationship satisfaction of trans people were more likely to identify as queer, fluid, or asexual compared to trans men and trans women, while Rutherford et al. (2021) highlighted that non-binary participants in their study of gay, bisexual, and other men who have sex with men and non-binary individuals over 15 living in Canada were significantly more likely than cisgender men to identify as queer (49.3% vs. 6.6%), pansexual (32.0% vs. 2.8%), bisexual (17.3% vs. 10.4%), asexual (4.7% vs. 0.6%), and other (2.7% vs. 0.4%). Rothblum et al. (2020) also found that US asexual participants from the Generations Study were significantly more likely to identify as non-binary than allosexual LGB individuals. Similarly, Nimbi et al. (2024) highlighted a high percentage of non-binary people in their adult Italian asexual sample, with non-binary participants mainly identifying with the labels asexual (22.9%) and gray-asexual (21.8%).

Lastly, in terms of developmental milestones of sexual identity (e.g., realization of LGB identity, first time having sex with someone of same sex, coming out with friends and family), non-binary people appeared to generally meet the majority of these milestones before adulthood (Bishop et al., 2023).

#### Sexual Attraction

Attraction was mainly asked about in relation to men and women, comparing non-binary people to individuals of different binary genders. Non-binary individuals reported attraction toward both men and women (Anzani & Prunas, 2020; Goldberg et al., 2020; Reisner et al., 2023). It is unclear whether this might be a significant difference in relation to binary individuals, with some evidence pointing out that AFAB non-binary people might be more likely to report attraction to both men and women than binary cis and trans people (Jacobson & Joel, 2019) and that non-binary individuals might present higher levels of attraction to women compared to cisgender males and females (Kennis et al., 2022b). However, other studies have found no significant differences in levels of attraction toward binary genders when comparing non-binary and binary trans individuals (Boskey & Ganor, 2022; Kennis et al., 2022a). Analysis on attraction toward non-binary genders was reported by one paper (Boskey & Ganor, 2022), finding that non-binary AFAB people were significantly more likely to report attraction to other non-binary genders compared with trans men. This is supported by descriptive findings in Reisner et al. (2023) showing that the majority of the non-binary people in their US probability sample were attracted to other non-binary people (76.1% to AFAB non-binary and 63.6% to AMAB non-binary individuals), and around 78% reported attraction to more than 3 genders.

#### Gender of Sexual Partners

Regarding the gender of sexual partners, both Holt et al. (2023) and Boskey and Ganor (2022) found that non-binary participants were significantly more likely than binary transgender individuals to have had non-binary partners. And, although no separated inferential analyses were conducted, Hibbert et al. (2020) found that among a sample of UK LGBT adults, 56% of non-binary participants reported having had sex with men, 77% reported sex with women, and 67% reported sex with non-binary people. Similarly, Reisner et al. (2023) found that 56.0% of non-binary participants in their US representative sample had had sex with cisgender women, 39.1% with cisgender men, 21.8% with trans women, 28.9% with trans men, and overall, 33.2% had had sex with partners of more than 3 different genders in the past 5 years. Additionally, Almås et al. (2024) found that non-binary people in their trans and gender-diverse Norwegian sample were more likely to report having had their last sex with an intersex or trans partner. Finally, in a sample of trans youth (aged 14–21), including trans non-binary youth, participants were asked about the gender of their sexual partners in the past 12 months (Fisher et al., 2018). Among 32 non-binary participants, 56.3% reported cisgender male partners, 43.8% reported transmasculine partners, 37.5% reported cis female partners, and 15.6% reported transfeminine partners; no significant differences by gender identity were found (Fisher et al., 2018).

#### Sexual Fantasy

Three papers reported exploring non-binary people’s sexual fantasies. Although there were no significant differences in fantasies about dominance/control and partner humiliation between non-binary and cisgender participants, non-binary people reported significantly less arousal than cisgender adults in relation to some fantasies (such as kink or sexual engagement with attractive and older/more experienced partners; Anzani & Prunas, 2020). The same authors identified that non-binary adults reported to be as excited by group sex/promiscuity and submission related fantasies as cisgender women (but significantly less than cisgender men). Finally, non-binary participants appeared to be significantly less aroused by fantasizing on undressing and showing devotion toward a partner than cis women, but at a comparable level to cis men (Anzani & Prunas, 2020). In relation to BDSM fantasies, one paper found that non-binary people reported more domination fantasies than cis women (*M* = 44.58, SD = 16.39 vs. *M* = 35.83, SD = 15.44) and more submissive fantasies than cis men (*M* = 43.37, SD = 19.14 vs. *M* = 53.42, SD = 17.85; Dahl et al., 2024). The same paper also explored BDSM behavior and found that cis women had significantly less dominant behavior than non-binary people (*M* = 30.40, SD = 13.63 vs. *M* = 38.28, SD = 15.84; Dahl et al., 2024). Additionally, Almås et al. (2024) reported that participants with a “nonbinary gender identity orientation” reported being more aroused by the idea of perceiving themselves as trans compared to binary trans participants (“female/male gender identity orientation”).

#### Sexual Behavior

##### Higher Risk Behaviors

In terms of higher risk sexual behavior, Kattari et al. (2019) reported that 42.1% of non-binary high school students in their sample used “drugs and alcohol” before last intercourse and 61.4% did not use a condom. Similarly, Smalley et al. (2016) found that 41.4% of non-binary participants in their sample reported having unprotected sex (defined as not using condoms or dental dams) at least most of the time and 8% reported having had sex “under the influence”, which was similar as compared to participants of different genders. A study including trans people attracted to men living in New Zealand found that being non-binary AFAB and attracted to men was linked to lower perceived ability to negotiate protective barrier use compared to trans women attracted to men (Byrne et al., 2022).

Conversely, one study including Australian transgender individuals found that non-binary people were as likely as trans binary people to have had condomless intercourse in the last year, but more likely to report ever having used drugs for sex (Holt et al., 2023).

One paper looked at casual sex during Covid-19 lockdown, finding that this was not associated with gender identity (Nadarzynski et al., 2023).

Lastly, one paper found that being non-binary was not significantly associated with higher likelihood of having had sex with someone of unknown STI/HIV status in the past 12 months (Pletta et al., 2022), while a different paper focusing on transactional sex highlighted that among 65 non-binary clients accessing Alabama-based AIDS service, 60 had no history of selling sex (Atkins et al., 2024).

##### Number of Sexual Partners

Number of sexual partners in the last year and across the lifetime was similar between non-binary and binary transgender aged 14 to 21 (2.7 partners in the last 12 months and around 5 lifetime partners; Fisher et al., 2018) and between non-binary and cisgender people over 16 (with around 9.9 lifetime sexual partners; McKenna et al., 2021).

##### Being Sexually Active and Other Sexual Behaviors

Holt et al. (2023) found that non-binary participants were more likely than binary individuals to report sex in the last year. Additionally, non-binary people of different sexual identities (queer lesbian/gay, bisexual, or “other”) were equally as likely to report being sexually active in the past 5 years (Goldberg et al., 2020). Furthermore, gender was not associated with reporting receptive or insertive sex in a sample of transfeminine and non-binary adults (Goldbach et al., 2023). In a Norwegian study of non-binary adults, the majority reported engaging in kissing and masturbation, while 42.4% reported having had oral sex in their last partnered sexual encounter (Almås et al., 2024). Lastly, the same paper discussed masturbation, finding that non-binary participants reported higher rates of masturbation compared to “female gender identity oriented” people (i.e., trans people with a feminine gender identity) but not those who were “male gender identity oriented” (i.e., trans people with a masculine gender identity; Almås et al., 2024).

#### Sexual Fluidity

The two papers that examined sexual fluidity both focused on the US context. The first paper (Katz-Wise et al., 2016) measured sexual fluidity in an adult American LGBT sample and found that being non-binary was associated with experiencing shifts in sexual attraction. Overall, 42% of non-binary individuals reported a change in their attractions across their lifetime, and being non-binary was significantly associated with ever experiencing a change in one’s sexual attraction for the whole sample; nonetheless, when restricting the analysis to a subset of individuals that had socially transitioned (*n* = 205), individuals that experienced a change in their attraction post-transition were less likely to be non-binary (Katz-Wise et al., 2016). The second article (Katz-Wise et al., 2023) found that among a sample of youth aged 14–25 years, recruited through the survey panel provider Prodege, non-binary participants were significantly more likely to report sexual identity changes compared to cisgender individuals (73.1% vs. 17.2% cis women and 7.8% cis men). Gender identity was also linked to changes in sexual attraction, with non-binary or other identity participants reporting the highest level of change (71.3% non-binary people, 39.2% cis women, 17.7% cis men, 50% trans women, 66% trans men; Katz-Wise et al., 2023).

#### Sexual Satisfaction and Relationship Satisfaction

Three papers looked at both sexual and relationship satisfaction (Holt et al., 2023; Mark et al., 2018; Perez & Pepping, 2024) and found no significant differences by gender. Similar null results have been reported elsewhere for relationship satisfaction (Fuller & Riggs, 2021; Walters et al., 2023) and sexual satisfaction (Kennis et al., 2022b) among binary trans people and non-binary people.

#### Relationship Structure

Three papers discussed monogamous and non-monogamous relationship structures in relation to non-binary individuals. Non-binary people were more likely than cisgender men (16.7% vs. 1.8%) to be in polyamorous relationships (Rutherford et al., 2021) and to have an open, “other type” of non-monogamous relationship or multiple relationships compared to trans men and women (non-binary people 29.7% vs. 16.2% trans men and 17.1% trans women; Holt et al., 2023). Lastly, on a descriptive level, non-binary adults currently living in the UK reported relationships with multiple partners more often than binary trans and cisgender individuals (9% non-binary participants vs. 6% trans women, 4% trans men, and 2% cis participants; Hibbert et al., 2020).

#### Sexual Health Attendance and Negative Experiences in Health Care

Four studies looked at sexual health care attendance and discrimination experiences. In a sample of LGBT UK adults, identifying as non-binary was not associated with sexual health clinic attendance compared to other trans people (Hibbert et al., 2020). In another study with Australian trans and gender-diverse participants over the age of 16, non-binary individuals presumed AFAB were significantly more likely to access sexual health care from a general practitioner, while non-binary participants presumed AMAB were more likely to access community-based sexual health services; there was no difference for accessing hospital-based sexual health care (Rosenberg et al., 2021).

In terms of negative experiences and discrimination within sexual health care, one paper found that AFAB non-binary individuals reported the most “gender insensitivity” (i.e., experiences of transphobia and cisgenderism), and non-binary participants in general found hospitals to be the most gender insensitive providers while community-based services were the least insensitive (Rosenberg et al., 2021). Additionally, in a Canadian sample including 3083 cis men who have sex with men and 150 non-binary participants, non-binary people were significantly less likely to report never having been denied sexual health care treatment compared to cisgender participants (68% vs. 84.2%; Rutherford et al., 2021).

#### Other Sexual Variables: Sexual Function, Well-Being, Self-Concept, Consent Attitudes, Porn Use, Assertiveness, Pleasure, and Desire

Other sexual variables were considered in single studies only and tended to focus on whether significant differences occurred between non-binary and binary (both trans and cis) individuals. Regarding sexual desire (Mark et al., 2018) and sexual assertiveness (McKenna et al., 2021), there were no significant differences between cisgender and non-binary participants; however, non-binary people reported fewer maladaptive attitudes, beliefs, and behaviors toward sexual consent (such as believing that consent could be assumed or that there is less need for consent as relationship length increases; McKenna et al., 2021).

Non-binary people reported greater discrepancies than cisgender people (but not trans binary people) in relation to “actual/ought” sexual self-concept (their ideas of who they currently are and their self-imposed expectation on who they should be in relation to their sexuality) and “actual/ideal” sexual self-concept (their ideas of who they are compare to who they would like to be as a sexual being; Kennis et al., 2022a). The same authors looked at sexual well-being as a function of sexual self-esteem, satisfaction, and body image worries, and found that trans non-binary people differed from trans binary people only on body image worries (with binary transgender individuals being more concerned about their bodies in a sexual setting; Kennis et al., 2022a). Non-binary people differed from cisgender people only on body perception, a sub-dimension of sexual self-esteem which looks at feelings of satisfaction toward one’s body and its current development level (Kennis et al., 2022a). On a descriptive level, non-binary participants also reported more sexual distress (*M* = 3.76; SD = 2.87) as compared with cis men (*M* = 3.41; SD = 2.75) and women (*M* = 3.15; SD = 2.66; Lin et al., 2024). Gender identity was not significantly associated with sexual functioning in a transmasculine sample including both binary and non-binary individuals (Reisner et al., 2020). It was also not associated with importance of sexual activity in a sample of binary transfeminine and non-binary AMAB adults (Goldbach et al., 2023). In terms of motivations for pornography use, results from an international convenience sample (42 countries) found that self-exploration was the only motivation which was more strongly endorsed by non-binary people compared to men and women (Koós et al., 2024). One paper (Almås et al., 2024) found that, as compared with non-binary people, people with a male gender identity orientation (i.e., trans men) reported higher sexual satisfaction when seducing and dominating their partners, but lower sexual satisfaction from using clothing sexually and engaging in other fetish behavior.

Lastly, Perez and Pepping (2024) looked at sexual fetishization in a sample of North American and European trans and gender-diverse adults. They found that trans women were significantly more likely than non-binary people to have experienced fetishization on social media, to have been sexually objectified, and to have had sexual partners who were only interested in them because of their trans identity. No differences between non-binary people and trans men were found (Perez & Pepping, 2024).

## Discussion

The current systematic review aimed to analyze the existing quantitative research on the sexuality and sexual health of non-binary people to synthesize and evaluate the literature results, appraise language inclusivity, and discuss potential gaps in knowledge. Most papers that investigated sexual orientation reported that non-binary people typically endorsed non-monosexual identities and identities that do not reference gender (e.g., queer, pansexual). This might reflect a tendency of non-binary individuals to move away from sexual identity labels that are not reflective of their gender expansiveness (Kuper et al., 2012). Moreover, non-binary people might be more sexually fluid than binary people (Katz-Wise, 2016), and might be more likely to be attracted to and have sexual relationships with other non-binary people compared to trans binary individuals (Boskey & Ganor, 2022). Qualitative findings about the experiences of sexual fluidity in gender minority individuals have linked shifts in sexual orientation to changes in gender identity (Galupo et al., 2014), but more specific studies with non-binary individuals that recognize their unique experiences are lacking and more research is needed.

In relation to sexual health, studies found that non-binary people often experienced cisgenderism and transphobia from their care providers, which has been linked with negative health effects, such as less frequent STI testing in gender-diverse individuals (Rosenberg et al., 2021). Negative attitudes and a lack of understanding of gender minority needs might deter non-binary individuals from engaging with professional support for sexual health (as highlighted by Rutherford et al., 2021). Furthermore, even when non-binary people decide to engage with health professionals, it is important to consider how gaps in knowledge and disregard for non-binary people’s identities might affect the quality of care provided (Rutherford et al., 2021). Although more research on these topics is necessary, this review found no reported significant differences between non-binary and binary people in terms of sexual and relationship satisfaction (e.g., Fuller et al., 2021; Perez & Pepping, 2024). Overall, research on other sexual variables is lacking, which greatly impacts the ability to generalize findings.

Another topic of interest for this review was the use of inclusive language in quantitative sex research with non-binary participants. Generally speaking, studies that measured sexual attraction and behavior did so in a way that ignored genders outside the binary, focusing on attraction toward men and woman only. Consistently, although most studies included an option to articulate one’s sexual identity label using a text box, some of the analyzed papers included only a small range of default options, which might not be appropriate for non-binary people. Importantly, an understanding of sexual orientation that is rooted in a binary conceptualization of gender might be inadequate and reductive for individuals who endorse a trans and/or non-binary identity (Galupo et al., 2014).

In relation to other sexual variables, although not all items used were reported, overall language appeared to be gender neutral, with authors sometimes changing the original scale’s verbiage to improve inclusivity. In some cases, “binary” pronouns, a gendered prospective or references to biological sex (Anzani & Prunas, 2020; Fuller & Riggs, 2021; Mark et al., 2018) were maintained, which could be problematic and may alienate non-binary respondents. Additionally, questions on sexual behavior that describe sexual acts might benefit from a more expansive definition of sexual activity that focuses less on penetration and genitals during sex (Goldbach et al., 2023).

Reviewed papers had some limitations. In relation to study characteristics, all papers were cross-sectional (or only performed cross-sectional analysis), which limits our understanding of how sexual and relationship related variables might evolve over time. Additionally, authors often did not, or were unable to, include non-binary people as a standalone group for their inferential analysis. This resulted in all individuals who did not neatly fit the gender binary being relegated to an “other-gender” category or in non-binary and binary transgender people being grouped together. Consequently, much of the information reported in this review comes from descriptive statistics, limiting our ability to generalize results to the wider non-binary population. Moreover, trans identification within the non-binary population was mostly disregarded and not assessed which could impact our understanding of how non-binary people who identify as trans and those who do not, differ in relation to their sexuality. Gender categorization in quantitative research should explore cisgender/transgender and binary/non-binary self-classifications and account for uncommon labels as chosen by participants (Beischel et al., 2023).

The assimilation of different gender identities in a single subgroup appeared to be due to the inability of most studies to recruit a significant number of non-binary individuals, to the point where meaningful statistics that focused on a non-binary subgroup could not be performed. Issues with recruitment may be partially explained by the fact that non-binary people are a relatively small and marginalized population (e.g., at the 2021 UK census, non-binary people represent 0.06% of people over 16 living in England and Wales; Office for National Statistics, 2023). Moreover, the recruitment of non-binary people (or even inclusion of non-binary people in some cases) was often not a specific focus of the studies, which tended to be more interested in reaching broader identity groups (e.g., transgender, LGBTQ + , or “gender minority” individuals; e.g., Rutherford et al., 2021).

We also found that measures used to assess sexual variables by the included studies were mostly created ad hoc and/or were not validated for use with non-binary or transgender populations. This reflects a more pervasive issue with research on gender-diverse individuals: a lack of validated psychometric tools for gender minority populations (Keo-Meier & Fitzgerald, 2017). To address these limitations, some of the included papers (10/44) performed psychometric testing, such as confirmatory or exploratory analysis or reliability testing (e.g., Anzani & Prunas, 2020).

From our analysis, it is apparent that this field is expanding and that literature on non-binary people is becoming more prevalent (as supported by the fact that 33/44 papers included in this review were published between 2020 and 2024). Nonetheless, research in this area is in its infancy. Notably, during the screening phase of this review, most of the research identified by the search strategy was not inclusive of non-binary people and either did not differentiate between trans and non-binary samples or did not mention non-binary people at all. Accordingly, the majority of papers retrieved via searching were not included in this review (see Supplementary Table 2). Even when the papers met inclusion criteria, non-binary people often seemed to be an afterthought, with measures lacking appropriate validation and language representing experiences outside of binarism. This is particularly true of older research included in this review. More empirically sound research, specifically focusing on the non-binary population as the main group of interest, is especially needed.

Finally, the wider societal impact of non-binary inclusive research warrants significant attention, with data showing a growing trend among younger generations to identify outside the gender binary (e.g., Gallup Organization, 2024). The prevalence of non-binary individuals is likely to continue to increase (e.g., Statistics Canada, 2022), along with increased societal recognition of those who exist beyond traditional Western gender norms. However, legal recognition of non-binary identities is progressing at a slower pace, as is healthcare providers’ ability to offer evidence-based support to non-binary people concerning their health in general, and their sexual health in particular (e.g., Kinney & Cosgrove, 2022). Consequently, non-binary individuals often bear the burden of advocating for their own care and relying on social support to escape transphobia within medical settings (Seelman & Poteat, 2020). As researchers in the field of sexuality, we have the ability and the responsibility to produce knowledge that reflects societal diversity, recognizing and fostering social change.

### Recommendations for Future Research

Based on the described findings and current limitations of the field, some recommendations can be made. In general, more research of non-binary experiences on sexual topics, such as sexual well-being, satisfaction in sexuality and relationships, sexual fluidity, fantasy, and assertiveness is needed. Such research needs to have appropriately powered samples, and where possible, a longitudinal design to assess changes over time. Studies that explore differences between binary transgender and non-binary individuals are especially lacking and this limits our understanding of possible differences between them. It is likely that binary transgender and non-binary individuals have different sexual needs and experiences which should be accounted for. In terms of measurement, there is a need to produce, adapt and/or validate scales with non-binary people to increase confidence in the legitimacy of results. When assessing sexual identity, presenting a variety of label options, particularly plurisexual and gender-neutral options like pansexual and queer, should be the norm. Similarly, non-binary identities should be included as response options in relation to sexual attraction and behavior related items (e.g., when assessing the gender identity of past, current, or potential partners), to allow non-binary people (and their partners) to more easily express their full range of sexual feelings and experiences. When conducting research with this population, authors also need to ensure that language is neutral and that any ambiguity (e.g., confusion between sex/gender) or unnecessary focus on sex assigned at birth is avoided. Finally, researchers need to ensure that gender diversity is kept at the forefront of research by involving non-binary people in studies about non-binary people from the very beginning.

### Limitations of This Systematic Review

This systematic review has several limitations. Firstly, the focus on western samples and definitions of what it means to be non-binary might have created bias. However, this was an intentional choice because of variations in cultural understandings about non-binary identities. Additionally, we recognize that western categorizations of non-binary identities are also subjected to cultural influences, which can further limit our analysis. Secondly, this review focused only on quantitative research published in English and Italian, and we are therefore unable to discuss how qualitative studies might have addressed sexual variables in non-binary populations and in other languages. Selection bias might have impacted the researchers’ impartiality; however, this was addressed by means of detailed inclusion/exclusion criteria and two independent coders being involved in the screening process.

### Conclusion

Although studies that include non-binary people are increasing, literature with a specific focus on non-binary individuals is still severely lacking. In order to fill current gaps in knowledge in a meaningful way, researchers need to establish a clearer focus on individuals that challenge and/or disrupt gender binarism by validating tools for non-binary people, using inclusive language, and enhancing community consultation practices.

Amplifying non-binary voices within academia has the potential to generate credible and impactful research that provides a more nuanced understanding of non-binary people’s sexuality. This will directly benefit the sexual health of gender-diverse populations who have been previously marginalized both within and outside of the research literature focusing on sexual experience.

## Supplementary Information

Below is the link to the electronic supplementary material.

Supplementary file1 (DOCX 286 KB)

## Data Availability

Not applicable. No new data were created during this study.
