# Supplementary material for: Non-Binary People’s Sexuality, Sexual Health, and Relationship Satisfaction: A Review of 12 Years of Quantitative Research (2012–2024)
Source: Arch Sex Behav. 2025 Sep 2;54(8):3127–88. doi: 10.1007/s10508-025-03224-0 (PMC12484307; doi:10.1007/s10508-025-03224-0)
Supplement: Supplementary file 1 — Supplementary file1 (DOCX 286 KB) [file 10508_2025_3224_MOESM1_ESM.docx]

Supplementary Table 1: Search strategy/keywords and subject/mesh headings

| Sexuality, sexual health, relationships | English |
| --- | --- |
|  | Sexuality OR “Sexual Orientation*” OR “Sexual Fluidity” OR “Sexual satisfaction” OR “Sexual pleasure*” OR “Sexual Well*” OR “Sexual Health” OR “Sexual Function*” OR “Sexual Dysfunction*” OR “Sexual* dissatisf*” OR “Relationship Satisfaction” OR “Relationship quality” OR Orgasm OR “Sexual Fantas*” OR “Sexual Preference*” OR “Sexual* Distress*” Or “Sexual experience” OR “Romantic Relationship” |
|  | Italian |
|  | Sessual* OR “Orientamento sessuale” OR “Fluidità sessuale” OR “Salute sessuale” OR “Piacere sessuale” OR “Benessere sessuale” OR “Funzion* sessuale” OR “Disfunzion* sessuale” OR “Soddisfazione relazion*” OR “Benessere di coppia” OR “Relazione di coppia” OR Orgasmo |
| Gender identity | English |
|  | “Non-binary” OR nonbinary OR genderqueer OR “gender queer” OR “gender creative” OR “gender minority” OR “gender diverse” OR “gender non-conforming” OR “gender nonconforming” OR “LGBT*”OR Transgender OR Trans OR Transmasc* OR Transfem* OR Transwom* OR Transm* OR FTM OR MTF OR AFAB OR AMAB OR “Female to male” OR “Male to Female” OR Transsexual |
|  | Italian |
|  | Non-binari* OR “Identità di genere” OR “LGBT*”OR Transgender OR Trans OR Transmasc* OR Transfem* OR “donn* transgender” OR “uom* transgender” OR FTM OR MTF OR Gender OR Genere OR Transessual* |
| Subject headings and Mesh headings | PsycINFO |
|  | •DE "Gender Reassignment" OR DE "Gender Identity" OR DE "Gender Nonbinary" OR DE "Gender Nonconforming" OR DE "LGBTQ" OR DE "Transsexualism" OR DE "Transgender" OR DE "Transvestitism"  •DE "Sexuality" OR DE "Sexual Arousal" OR DE "Female Sexual Dysfunction" OR DE "Sexual Minority Groups" OR DE "Sexual Health" OR DE "Sexual Satisfaction" OR DE "Sexual Fantasy" OR DE "Sexual Attraction" OR DE "Orgasm" OR DE "Sexual Orientation" OR DE "Same sex intercourse" OR DE "Sexual function disturbances" OR DE "Sexual disorders" OR DE "Relationship Quality" OR DE "Relationship Satisfaction" OR DE "Same Sex Couples" |
|  | MEDLINE |
|  | •(MH "Sexuality") OR (MH "Sexual Dysfunction, Physiological") OR (MH "Sexual Health") OR (MH "Sexual Dysfunctions, Psychological") OR (MH "Orgasm") OR (MH "Erectile Dysfunction") OR (MH "Marriage")  •(MH "Gender Identity") OR (MH "Transgender Persons") OR (MH "Transsexualism") |

Supplementary Table 2: Excluded papers

| **Reasons for exclusion** | **Study** |
| --- | --- |
| **Samples from outside western countries** | Barrientos Delgado, J., Saiz, J. L., Guzmán-González, M., Bahamondes, J., Gómez, F., Castro, M. C., Espinoza-Tapia, R., Saavedra, L. L., & Giami, A. J. (2021). Sociodemographic characteristics, gender identification, and gender affirmation pathways in transgender people: A survey study in Chile. *Archives of Sexual Behavior*, *50*(8), 3505-3516. https://doi.org/10.1007/s10508-021-01939-4  Chakrapani, V., Newman, P. A., Shunmugam, M., Logie, C. H., & Samuel, M. (2017). Syndemics of depression, alcohol use, and victimisation, and their association with HIV-related sexual risk among men who have sex with men and transgender women in India. *Global Public Health: An International Journal for Research, Policy and Practice*, *12*(2), 250-265. https://doi.org/10.1080/17441692.2015.1091024  Chakrapani, V., Shunmugam, M., Newman, P. A., Kershaw, T., & Dubrow, R. (2015). HIV status disclosure and condom use among HIV-positive men who have sex with men and Hijras (male-to-female transgender people) in India: Implications for prevention. *Journal of HIV/AIDS & Social Services*, *14*(1), 26-44. https://doi.org/10.1080/15381501.2013.859113  Champutiz-Quintana, K. A., Olmos, J. C. C., Lucas-Matheu, M., & Bastidas-Champutiz, S. B. (2024). Assessment of sexual satisfaction in the LGBTIQ population of Ecuador. *UNIVERSITAS-REVISTA DE CIENCIAS SOCIALES Y HUMANAS*(40), 187-208. https://doi.org/10.17163/uni.n40.2024.08  Haase, S., Müller, A., & Zweigenthal, V. (2022). Sexual health behavior, health status, and knowledge among queer womxn and trans men in Kenya: An online cross-sectional study. *PloS one*, *17*(6). https://doi.org/10.1371/journal.pone.0268298  Lee, Y. G., Zhakupova, G., Vinogradov, V., Paine, E. A., Laughney, C. I., Reeder, K., Davis, A., Hunt, T., Mergenova, G., Primbetova, S., Terlikbayeva, A., & Wu, E. (2022). Polydrug use, sexual risk, and HIV testing among cisgender gay, bisexual, and other men and transgender and nonbinary individuals who have sex with men in Kazakhstan. *AIDS Education and Prevention*, *34*(5), 413-426. https://doi.org/10.1521/aeap.2022.34.5.413  Nugroho, A., Erasmus, V., Coulter, R. W. S., Koirala, S., Nampaisan, O., Pamungkas, W., & Richardus, J. H. (2018). Driving factors of retention in care among HIV-positive MSM and transwomen in Indonesia: A cross-sectional study. *PloS one*, *13*(1). https://doi.org/10.1371/journal.pone.0191255  Petterson, L. J., Dixson, B. J., Little, A. C., & Vasey, P. L. (2018). Viewing time and self-report measures of sexual attraction in Samoan cisgender and transgender androphilic males. *Archives of Sexual Behavior*, *47*(8), 2427-2434. https://doi.org/10.1007/s10508-018-1267-7  Smith, A. D., Kimani, J., Kabuti, R., Weatherburn, P., Fearon, E., & Bourne, A. (2021). HIV burden and correlates of infection among transfeminine people and cisgender men who have sex with men in Nairobi, Kenya: an observational study. *The lancet. HIV*, *8*(5), e274-e283. https://doi.org/10.1016/S2352-3018(20)30310-6  Stief, M. (2017). The sexual orientation and gender presentation of hijra, kothi, and panthi in Mumbai, India. *Archives of Sexual Behavior*, *46*(1), 73-85. https://doi.org/10.1007/s10508-016-0886-0  Su, Y., & Zheng, L. (2023). Stability and Change in Asexuality: Relationship Between Sexual/Romantic Attraction and Sexual Desire. *Journal of Sex Research*, *60*(2), 231-241. https://doi.org/10.1080/00224499.2022.2045889  Wainipitapong, S., Oon-arom, A., Wiwattarangkul, T., Vadhanavikkit, P., Wiwattanaworaset, P., Srifuengfung, M., & Chiddaycha, M. (2023). Sexual Behaviors Among Lesbian, Gay, Bisexual, Transgender, and Other Sexual and Gender Diverse Medical Students: A National Study of Thai Medical Schools. *International Journal of Sexual Health*, *35*(3), 352-362. https://doi.org/10.1080/19317611.2023.2214801 |
| **Not empirical analysis** | Agwu, A. (2020). Sexuality, Sexual Health, and Sexually Transmitted Infections in Adolescents and Young Adults. *Topics in antiviral medicine*, *28*(2), 459-462. https://search.ebscohost.com/login.aspx?direct=true&db=cmedm&AN=32886466&site=ehost-live  https://www.ncbi.nlm.nih.gov/pmc/articles/PMC7482983/pdf/tam-28-459.pdf  Bauer, G. R., & Hammond, R. (2015). Toward a broader conceptualization of trans women's sexual health. *Canadian Journal of Human Sexuality*, *24*(1), 1-11. https://doi.org/10.3138/cjhs.24.1-CO1  Berenbaum, S. A., & Meyer-Bahlburg, H. F. L. (2015). Gender development and sexuality in disorders of sex development. *Hormone and metabolic research = Hormon- und Stoffwechselforschung = Hormones et metabolisme*, *47*(5), 361-366. https://doi.org/10.1055/s-0035-1548792  Carr, N., Serisier, T., & McAlister, S. (2020). Sexual deviance in prison: Queering identity and intimacy in prison research. *Criminology & Criminal Justice: An International Journal*, *20*(5), 551-563. https://doi.org/10.1177/1748895820937401  da Silva, R. U. M., Abreu, F. J. d. S., da Silva, G. M. V., Dos Santos, J. V. Q. V., Batezini, N. S. d. S., Silva, B. N., & Rosito, T. E. (2018). Step by step male to female transsexual surgery. *International braz j urol : official journal of the Brazilian Society of Urology*, *44*(2), 407-408. https://doi.org/10.1590/S1677-5538.IBJU.2017.0044  Dowshen, N., Matone, M., Luan, X., Lee, S., Belzer, M., Fernandez, M. I., & Rubin, D. (2016). Behavioral and health outcomes for HIV+ young transgender women (YTW) linked to and engaged in medical care. *LGBT Health*, *3*(2), 162-167. https://doi.org/10.1089/lgbt.2014.0062  Elyaguov, J., Schardein, J. N., Sterling, J., & Nikolavsky, D. (2022). Gender Affirmation Surgery, Transfeminine. *The Urologic clinics of North America*, *49*(3), 437-451. https://doi.org/10.1016/j.ucl.2022.05.001  Jaspal, R., Nambiar, K. Z., Delpech, V., & Tariq, S. (2018). HIV and trans and non-binary people in the UK. *Sexually transmitted infections*, *94*(5), 318-319. https://doi.org/10.1136/sextrans-2018-053570  Salvador, J., Massuda, R., Andreazza, T., Koff, W. J., Silveira, E., Kreische, F., de Souza, L., de Oliveira, M. H., Rosito, T., Fernandes, B. S., & Lobato, M. I. R. (2012). Minimum 2‐year follow up of sex reassignment surgery in Brazilian male‐to‐female transsexuals. *Psychiatry and Clinical Neurosciences*, *66*(4), 371-372. https://doi.org/10.1111/j.1440-1819.2012.02342.x  Wright, J., & Greenberg, E. (2024). Non-binary youth and binary sexual consent education: unintelligibility, disruption and possibility. *SEX EDUCATION-SEXUALITY SOCIETY AND LEARNING*, *24*(4), 445-459. https://doi.org/10.1080/14681811.2023.2217748  Yeung, H., Luk, K. M., Chen, S. C., Ginsberg, B. A., & Katz, K. A. (2019). Dermatologic care for lesbian, gay, bisexual, and transgender persons: Epidemiology, screening, and disease prevention. *Journal of the American Academy of Dermatology*, *80*(3), 591-602. https://doi.org/10.1016/j.jaad.2018.02.045 |
| **No full text** | Ansermet, F. (2014). Scegliere il proprio sesso: Usi contemporanei della differenza sessuale = Choose your gender: Current suggestions on gender differences. *Rivista Sperimentale di Freniatria: La Rivista della Salute Mentale*, *138*(2), 11-22. https://doi.org/10.3280/RSF2014-002002  Romano, G., Bouaoud, J., Schmidt, M., Rausky, J., Stivala, A., Atlan, M., & Cristofari, S. (2023). Improvements in Transgender Masculinizing Chest Surgery: A Pilot Study of a Tailored Approach with a Life Satisfaction Assessment. *Transgender Health*. https://doi.org/10.1089/trgh.2021.0212 |
| **Not in English or Italian** | Alavi, K., Nodushan, A. H. J., & Eftekhar, M. (2014). Sexual orientation in patients with gender identity disorder. *Iranian Journal of Psychiatry and Clinical Psychology*, *20*(1), 43-49. https://search.ebscohost.com/login.aspx?direct=true&db=psyh&AN=2014-36513-005&site=ehost-livemehrdad.eftekhar@gmail.com  Bergero, T., Ballester, R., Gornemann, I., Cano, G., & Asiain, S. (2012). Desarrollo y validación de un instrumento para la evaluación del comportamiento sexual de los transexuales: EL CSTM = Development and validation of an instrument for evaluating transgender sexual behavior: The CSTM. *Revista de Psicopatología y Psicología Clínica*, *17*(1), 11-30. https://doi.org/10.5944/rppc.vol.17.num.1.2012.10366  Cerwenka, S., Nieder, T. O., & Richter-Appelt, H. (2012). [Sexual orientation and partner-choice of transsexual women and men before gender-confirming interventions]. *Psychotherapie, Psychosomatik, medizinische Psychologie*, *62*(6), 214-222. https://doi.org/10.1055/s-0032-1309030  Cerwenka, S., Nieder, T. O., & Richter-Appelt, H. (2012). Sexuelle Orientierung und Partnerwahl transsexueller Frauen und Männer vor körpermedizinischen geschlechtsanpassenden Maßnahmen = Sexual orientation and partner-choice of transsexual women and men before gender-confirming interventions. *PPmP: Psychotherapie Psychosomatik Medizinische Psychologie*, *62*(6), 214-222. https://doi.org/10.1055/s-0032-1309030  Heß, J., Sohn, M., Küntscher, M., & Bohr, J. (2020). [Gender reassignment surgery from male to female]. *Der Urologe. Ausg. A*, *59*(11), 1348-1355. https://doi.org/10.1007/s00120-020-01337-z  Isaev, D. D. (2016). Deconstruction of Heteronormative Matrix. *Psychology-Journal of the Higher School of Economics*, *13*(1), 9-26. <Go to ISI>://WOS:000382813100002  Karpel, L., Gardel, B., Revol, M., Brémont-Weil, C., Ayoubi, J.-M., & Cordier, B. (2015). Bien-être psychosocial postopératoire de 207 transsexuels = Psychological and sexual well being of 207 transsexuals after sex reassignment in France. *Annales Médico-Psychologiques*, *173*(6), 511-519. https://doi.org/10.1016/j.amp.2012.01.021 |
| **Not quantitative analysis of sexual variables** | Anzani, A., Ruscio, E., Peverato, I., & Prunas, A. (2024). The use of sex toys in trans and nonbinary individuals' sexuality. *Sexual and Relationship Therapy*. https://doi.org/10.1080/14681994.2024.2306311  Chadwick, S. B., Francisco, M., & van Anders, S. M. (2019). When orgasms do not equal pleasure: Accounts of 'bad' orgasm experiences during consensual sexual encounters. *Archives of Sexual Behavior*, *48*(8), 2435-2459. https://doi.org/10.1007/s10508-019-01527-7  Davis, S. A., & Meier, S. C. (2014). Effects of testosterone treatment and chest reconstruction surgery on mental health and sexuality in female-to-male transgender people. *International Journal of Sexual Health*, *26*(2), 113-128. https://doi.org/10.1080/19317611.2013.833152  Galupo, M. P., Lomash, E., & Mitchell, R. C. (2017). 'All of my lovers fit into this scale': Sexual minority individuals’ responses to two novel measures of sexual orientation. *Journal of Homosexuality*, *64*(2), 145-165. https://doi.org/10.1080/00918369.2016.1174027  Galupo, M. P., Pulice-Farrow, L., Clements, Z. A., & Morris, E. R. (2019). 'I love you as both and I love you as neither': Romantic partners’ affirmations of nonbinary trans individuals. *International Journal of Transgenderism*, *20*(2-3), 315-327. https://doi.org/10.1080/15532739.2018.1496867  Hoskin, R. A., Blair, K. L., & Jenson, K. E. (2016). Dignity versus diagnosis: Sexual orientation and gender identity differences in reports of one’s greatest concern about receiving a sexual health exam. *Psychology & Sexuality*, *7*(4), 279-293. https://doi.org/10.1080/19419899.2016.1236745  Hwahng, S. J., & Nuttbrock, L. (2014). Adolescent gender-related abuse, androphilia, and HIV risk among transfeminine people of color in New York City. *Journal of Homosexuality*, *61*(5), 691-713. https://doi.org/10.1080/00918369.2014.870439  Rossiter, H. (2016). She's always a woman: Butch lesbian trans women in the lesbian community. *Journal of Lesbian Studies*, *20*(1), 87-96. https://doi.org/10.1080/10894160.2015.1076236  Scott, S. B., Pulice-Farrow, L., Do, Q. A., Garibay, B., & Balsam, K. F. (2023). 'The sense of falling in love again': Transgender and nonbinary individuals’ positive experiences in romantic relationships during gender transitions. *the Behavior Therapist*, *46*(3), 113-122. https://soton.idm.oclc.org/login?url=https://search.ebscohost.com/login.aspx?direct=true&db=psyh&AN=2024-33131-003&site=ehost-live  Thomann, M., Grosso, A., Wilson, P. A., & Chiasson, M. A. (2020). 'The only safe way to find a partner': rethinking sex and risk online in Abidjan, Cote d'Ivoire. *Critical Public Health*, *30*(1), 53-67. https://doi.org/10.1080/09581596.2018.1527017  Tompkins, A. B. (2014). 'There's no chasing involved': Cis/trans relationships, 'tranny chasers,' and the future of a sex-positive trans politics. *Journal of Homosexuality*, *61*(5), 766-780. https://doi.org/10.1080/00918369.2014.870448  Vu, B. N., Mulvey, K. P., Baldwin, S., & Nguyen, S. T. (2012). HIV risk among drug-using men who have sex with men, men selling sex, and transgender individuals in Vietnam. *Culture, Health & Sexuality*, *14*(2), 167-180. https://doi.org/10.1080/13691058.2011.630756  Williams, C. J., Weinberg, M. S., & Rosenberger, J. G. (2013). Trans men: Embodiments, identities, and sexualities. *Sociological Forum*, *28*(4), 719-741. https://doi.org/10.1111/socf.12056  Williams, C. J., Weinberg, M. S., & Rosenberger, J. G. (2016). Trans women doing sex in San Francisco. *Archives of Sexual Behavior*, *45*(7), 1665-1678. https://doi.org/10.1007/s10508-016-0730-6  Wood, S., Dowshen, N., Bauermeister, J. A., Lalley-Chareczko, L., Franklin, J., Petsis, D., Swyryn, M., Barnett, K., Weissman, G. E., Koenig, H. C., & Gross, R. (2020). Social support networks among young men and transgender women of color receiving HIV pre-exposure prophylaxis. *Journal of Adolescent Health*, *66*(3), 268-274. https://doi.org/10.1016/j.jadohealth.2019.08.014  Wood, S., Gross, R., Shea, J. A., Bauermeister, J. A., Franklin, J., Petsis, D., Swyryn, M., Lalley-Chareczko, L., Koenig, H. C., & Dowshen, N. (2019). Barriers and facilitators of PrEP adherence for young men and transgender women of color. *AIDS and Behavior*, *23*(10), 2719-2729. https://doi.org/10.1007/s10461-019-02502-y  Zaliznyak, M., Lauzon, M., Stelmar, J., Yuan, N., Smith, S. M., & Garcia, M. M. (2022). A Proposed Inventory to Assess Changes in Orgasm Function of Transgender Patients Following Gender Affirming Treatments: Pilot Study. *Sexual medicine*, *10*(3), 100510. https://doi.org/10.1016/j.esxm.2022.100510 |
| **Not an article** | Arayasirikul, S., & Wilson, E. (2015). THE INTERSECTION OF GENDER AND SEXUALITY: DIFFERENCES IN MENTAL HEALTH, ALCOHOL USE, AND SUBSTANCE USE AMONG YOUNG SEXUAL MINORITY TRANSGENDER WOMEN. *Alcoholism-Clinical and Experimental Research*, *39*, 304A-304A. <Go to ISI>://WOS:000361637102102  Boskey, E., & Ganor, O. (2021). SELF-REPORTED SEXUAL ORIENTATION AND ATTRACTION IN A COHORT OF BINARY- AND NON-BINARY TRANSMASCULINE PATIENTS SEEKING GENDER AFFIRMING SURGERY. *Journal of Sexual Medicine*, *18*(3), S74-S75. <Go to ISI>://WOS:000631178500139  Byrne, R., Chislett, L., & Patel, S. (2015). SEXUAL HEALTH IN TRANS* INDIVIDUALS: HIGH RISK AND UNDER REPRESENTED. *Sexually transmitted infections*, *91*, A91-A91. https://doi.org/10.1136/sextrans-2015-052126.271  Callander, D., Cook, T., Cornelisse, V., Pony, M., Duncan, D., Wiggins, J., Vlahakis, E., Duck-Chong, L., Rosenberg, S., & Holt, M. (2019). TRANS AND GENDER DIVERSE PEOPLE'S EXPERIENCES OF SEXUAL HEALTH CARE ARE ASSOCIATED WITH SEXUAL HEALTH SCREENING UPTAKE. *Sexually transmitted infections*, *95*, A64-A64. https://doi.org/10.1136/sextrans-2019-sti.167  Conner, C. K., Lamb, K. M., & Dermody, S. S. (2023). Access and barriers to health services among sexual and gender minority college students. *Psychology of Sexual Orientation and Gender Diversity*, *10*(3), 498-508. https://doi.org/10.1037/sgd0000559  Dave, T., Al-Hashimi, S., Curbera, N. M., Ross, M., & Brady, M. (2022). EXPERIENCES OF A TRANS-INCLUSIVE SEXUAL HEALTH AND WELL-BEING SERVICE. *Sexually transmitted infections*, *98*, A1-+. https://doi.org/10.1136/sextrans-BASHH-2022.3  Ellis, M. S. (2022). *Associations of state policy environments and state advocacy organizations on pathways to transgender health outcomes* ProQuest Information & Learning]. psyh. https://search.ebscohost.com/login.aspx?direct=true&db=psyh&AN=2021-94599-211&site=ehost-live  Fein, L. A., Estes, C. M., & Salgado, C. J. (2015). SEXUAL PREFERENCES AND PARTNERSHIPS OF TRANSGENDER WOMEN POST TRANSITION. *Journal of Sexual Medicine*, *12*, 282-282. <Go to ISI>://WOS:000356071800026  Garcia, M., Christopher, N., DeLuca, F., Spilotros, M., Garaffa, G., & Ralph, D. (2014). LIGHT-TOUCH, EROGENOUS SENSATION, AND SEXUAL FUNCTION AMONG TRANSGENDER MEN UNDERGOING SUPRAPUBIC PEDICLE AND RADIAL ARTERY FOREARM FREE-FLAP PHALLOPLASTY. *Journal of Urology*, *191*(4), E20-E21. https://doi.org/10.1016/j.juro.2014.02.148  Garcia, M., & Zaliznyak, M. (2020). EFFECTS OF FEMINIZING HORMONE THERAPY ON SEXUAL FUNCTION OF TRANSGENDER WOMEN. *Journal of Urology*, *203*, E672-E672. <Go to ISI>://WOS:000527010302703  Genc, M. A., Direk, N., & Aysevener, B. E. O. (2021). Attachment style, dyadic adjustment and gender roles attitudes of trans men and their partners in Turkey. *European Neuropsychopharmacology*, *53*, S587-S587. https://doi.org/10.1016/j.euroneuro.2021.10.663  Isernia, V., Phung, B., Lepretre, A. M., Azadi, B., Rincon, G., Zelie, J., Le Gac, S., Deprez, A., Michard, F., Yazdanpanah, Y., & Ghosn, J. (2021). Pre-exposure HIV prophylaxis (PrEP) among transgender women: 3 years of follow-up in a university hospital in Paris. *Sexually transmitted infections*, *97*(6), 465-466. https://doi.org/10.1136/sextrans-2020-054618  Janamnuaysook, R., Samitpol, K., Getwongsa, P., Chancham, A., Kongkapan, J., Amatsombat, T., Rueannak, J., Srimanus, P., Markhlur, N., Mingkwanrungruang, P., Meksena, R., Ramautarsing, R., Avery, M., Mills, S., Vannakit, R., Phanuphak, P., & Phanuphak, N. (2019). Integrating gender-affirming hormone treatment into HIV services facilitates access to HIV testing, syphilis testing, PrEP, and other sexual health services among transgender women in Thailand. *Journal of the International AIDS Society*, *22*, 61-61. <Go to ISI>://WOS:000476890500127  Kerckhof, M. E., Kreukels, B. P. C., Nieder, T. O., Becker-Hébly, I., van de Grift, T. C., Staphorsius, A. S., Köhler, A., Heylens, G., & Elaut, E. (2019). Prevalence of sexual dysfunctions in transgender persons: Results from the ENIGI follow-up study. *Journal of Sexual Medicine*, *16*(12), 2018-2029. https://doi.org/10.1016/j.jsxm.2019.09.003  Macapagal, K., Kraus, A., Moskowitz, D. A., & Birnholtz, J. (2020). 'Geosocial networking application use, characteristics of app-met sexual partners, and sexual behavior among sexual and gender minority adolescents assigned male at birth': Correction. *Journal of Sex Research*, *57*(8), i-i. https://doi.org/10.1080/00224499.2020.1716554  Panis, N. K., Spadaccini, L., Cabrera, N., Perez, C. F., Iannantuono, M. V., Sandoval, M. M., Doudtchitzky, N., Figueroa, M. I., Cesar, C., Fink, V., Frola, C., Sued, O., Aristegui, I., & Trans, C. S. G. (2022). Addressing sexual health in trans masculinities: lessons learned from TransCITAR transgender cohort study in Argentina. *Journal of the International AIDS Society*, *25*, 173-173. <Go to ISI>://WOS:000834867400330  Pigot, G., Bouman, M. B., Horvat, S., Buncamper, M., Mullender, M., Kreukels, B., & Meuleman, E. (2016). SEX REASSIGNMENT SURGERY (SRS) WITHOUT URETHRAL LENGTHENING IN FEMALE TO MALE (FtM) TRANSGENDERS. FUNCTIONAL OUTCOMES, PATIENT SATISFACTION AND SEXUAL FUNCTION. *Journal of Sexual Medicine*, *13*(5), S127-S127. https://doi.org/10.1016/j.jsxm.2016.03.125  Porter, K., & Ronneberg, C. (2014). SAMPLE CHARACTERISTIC DIFFERENCES BY SEXUAL ORIENTATION FROM THE TRANS MET LIFE SURVEY. *Gerontologist*, *54*, 155-155. <Go to ISI>://WOS:000346337501445  Skaletz-Rorowski, A., Nambiar, S., Basilowski, M., Wach, J., Kayser, A., Kasper, A., Brockmeyer, N., & Potthoff, A. (2022). Perspectives on trans-specific sexual health needs based on a free anonymous Online HIV/STI Risk Test (ORT) survey in Germany. *Sexually transmitted infections*. https://doi.org/10.1136/sextrans-2021-055356  Vedovo, F., Di Blas, L., Perin, C., Pavan, N., Zatta, M., Bucci, S., Morelli, G., Cocci, A., Delle Rose, A., Grisanti, S. C., Gentile, G., Colombo, F., Rolle, L., Timpano, M., Verze, P., Spirito, L., Schiralli, F., Bettocchi, C., Palmieri, A., Mirone, V., & Trombetta, C. (2019). OMTFSFI: OPERATED MALE TO FEMALE SEXUAL FUNCTION INDEX. DEVELOPMENT AND VALIDATION OF THE FIRST QUESTIONNAIRE TO ASSESS SEXUAL FUNCTION AFTER MALE TO FEMALE GENDER REASSIGNMENT SURGERY. *Journal of Urology*, *201*(4), E587-E587. <Go to ISI>://WOS:000473345202003  Vedovo, F., Pavan, N., Chiapparrone, G., Liguori, G., Barbone, F., & Trombetta, C. (2017). COMPARISON OF SEXUAL FUNCTION IN TRANSSEXUAL WOMEN WHO UNDERWENT SEX REASSIGNMENT SURGERY BY TWO DIFFERENT TECHNIQUES. *Journal of Sexual Medicine*, *14*(1), S122-S122. https://doi.org/10.1016/j.jsxm.2016.11.282  Vedovo, F., Pavan, N., Liguori, G., Bucci, S., Bertolotto, M., & Trombetta, C. (2017). NEOCLITORIS SIZE AND LOCATION: CAN THEY AFFECT TRANSSEXUAL WOMEN SEXUAL FUNCTION? A PRELIMINARY PELVIC MRI STUDY. *Journal of Urology*, *197*(4), E1225-E1225. https://doi.org/10.1016/j.juro.2017.02.2859 |
| **No focus on trans or non-binary people** | Burri, A. (2017). Sexual sensation seeking, sexual compulsivity, and gender identity and its relationship with sexual functioning in a population sample of men and women. *Journal of Sexual Medicine*, *14*(1), 69-77. https://doi.org/10.1016/j.jsxm.2016.10.013  Chung, P. H., Morey, A. F., Tausch, T. J., Simhan, J., & Scott, J. F. (2014). High submuscular placement of urologic prosthetic balloons and reservoirs: 2-year experience and patient-reported outcomes. *Urology*, *84*(6), 1535-1540. https://doi.org/10.1016/j.urology.2014.08.029  Currin, J. M., Hubach, R. D., & Crethar, H. C. (2015). Multidimensional assessment of sexual orientation and childhood gender nonconformity: Implications for defining and classifying sexual/affectional orientations. *Journal of LGBT Issues in Counseling*, *9*(4), 240-255. https://doi.org/10.1080/15538605.2015.1103676  Ediati, A., Juniarto, A. Z., Birnie, E., Drop, S. L. S., Faradz, S. M. H., & Dessens, A. B. (2015). Gender development in Indonesian children, adolescents, and adults with disorders of sex development. *Archives of Sexual Behavior*, *44*(5), 1339-1361. https://doi.org/10.1007/s10508-015-0493-5  Greaves, L. M., Barlow, F. K., Huang, Y., Stronge, S., Fraser, G., & Sibley, C. G. (2017). Asexual identity in a New Zealand national sample: Demographics, well-being, and health. *Archives of Sexual Behavior*, *46*(8), 2417-2427. https://doi.org/10.1007/s10508-017-0977-6  Greaves, L. M., Sibley, C. G., Fraser, G., & Barlow, F. K. (2019). Comparing pansexual- and bisexual-identified participants on demographics, psychological well-being, and political ideology in a New Zealand national sample. *Journal of Sex Research*, *56*(9), 1083-1090. https://doi.org/10.1080/00224499.2019.1568376  Greaves, L. M., Stronge, S., Sibley, C. G., & Barlow, F. K. (2021). Asexual identity, personality, and social motivations in a New Zealand national sample. *Archives of Sexual Behavior*, *50*(8), 3843-3852. https://doi.org/10.1007/s10508-021-02038-0  Hsu, K. J., Rosenthal, A. M., Miller, D. I., & Bailey, J. M. (2017). Sexual arousal patterns of autogynephilic male cross-dressers. *Archives of Sexual Behavior*, *46*(1), 247-253. https://doi.org/10.1007/s10508-016-0826-z  Li, G., Pollitt, A. M., & Russell, S. T. (2016). Depression and sexual orientation during young adulthood: Diversity among sexual minority subgroups and the role of gender nonconformity. *Archives of Sexual Behavior*, *45*(3), 697-711. https://doi.org/10.1007/s10508-015-0515-3  McInroy, L. B., Beaujolais, B., Craig, S. L., & Eaton, A. D. (2021). The Self-Identification, LGBTQ+ Identity Development, and Attraction and Behavior of Asexual Youth: Potential Implications for Sexual Health and Internet-Based Service Provision. *Archives of Sexual Behavior*, *50*(8), 3853-3863. https://doi.org/10.1007/s10508-021-02064-y  Pecora, L. A., Hancock, G. I., Hooley, M., Demmer, D. H., Attwood, T., Mesibov, G. B., & Stokes, M. A. (2020). Gender identity, sexual orientation and adverse sexual experiences in autistic females. *Molecular autism*, *11*(1), 57. https://doi.org/10.1186/s13229-020-00363-0  Xu, Y., Norton, S., & Rahman, Q. (2021). Childhood gender nonconformity and the stability of self-reported sexual orientation from adolescence to young adulthood in a birth cohort. *Developmental Psychology*, *57*(4), 557-569. https://doi.org/10.1037/dev0001164  10.1037/dev0001164.supp (Supplemental) |
| **No separate analysis for non-binary people** | Abraham, E., Chow, E. P. F., Fairley, C. K., Lee, D., Kong, F. Y. S., Mao, L. M., Goller, J. L., Medland, N., Bavinton, B. R., Sudarto, B., Joksic, S., Wong, J., Phillips, T. R., & Ong, J. J. (2023). eSexualHealth: Preferences to use technology to promote sexual health among men who have sex with men and trans and gender diverse people. *FRONTIERS IN PUBLIC HEALTH*, *10*, Article 1064408. https://doi.org/10.3389/fpubh.2022.1064408  Adan Sanchez, A. Y., McMillan, E., Bhaduri, A., Pehlivan, N., Monson, K., Badcock, P., Thompson, K., Killackey, E., Chanen, A., & O'Donoghue, B. (2019). High-risk sexual behaviour in young people with mental health disorders. *Early Intervention in Psychiatry*, *13*(4), 867-873. https://doi.org/10.1111/eip.12688  Agarwal, C. A., Scheefer, M. F., Wright, L. N., Walzer, N. K., & Rivera, A. (2018). Quality of life improvement after chest wall masculinization in female-to-male transgender patients: A prospective study using the BREAST-Q and Body Uneasiness Test. *Journal of plastic, reconstructive & aesthetic surgery : JPRAS*, *71*(5), 651-657. https://doi.org/10.1016/j.bjps.2018.01.003  Agénor, M., Lett, E., Ramanayake, N., Zubizarreta, D., Murchison, G. R., Eiduson, R., & Gordon, A. R. (2023). Racial/Ethnic Differences in Sexually Transmitted Infection Testing Among Transgender Men and Nonbinary Assigned Female at Birth Young Adults in the United States: a National Study. *JOURNAL OF RACIAL AND ETHNIC HEALTH DISPARITIES*, *10*(6), 2900-2910. https://doi.org/10.1007/s40615-022-01467-4  Allan-Blitz, L.-T., Herrera, M. C., Calvo, G. M., Vargas, S. K., Caceres, C. F., Klausner, J. D., & Konda, K. A. (2019). Venue-based HIV-testing: An effective screening strategy for high-risk populations in Lima, Peru. *AIDS and Behavior*, *23*(4), 813-819. https://doi.org/10.1007/s10461-018-2342-8  Allen-Leigh, B., Rivera-Rivera, L., Yunes-Díaz, E., Portillo-Romero, A. J., Brown, B., León-Maldonado, L., Vargas-Guadarrama, G., Salmerón, J., & Lazcano-Ponce, E. C. (2020). Uptake of the HPV vaccine among people with and without HIV, cisgender and transgender women and men who have sex with men and with women at two sexual health clinics in Mexico City. *Human vaccines & immunotherapeutics*, *16*(4), 981-990. https://doi.org/10.1080/21645515.2019.1675456  Andrinopoulos, K., Hembling, J., Guardado, M. E., de Maria Hernández, F., Nieto, A. I., & Melendez, G. (2015). Evidence of the negative effect of sexual minority stigma on HIV testing among MSM and transgender women in San Salvador, El Salvador. *AIDS and Behavior*, *19*(1), 60-71. https://doi.org/10.1007/s10461-014-0813-0  Andrzejewski, J., Pampati, S., Johns, M. M., Sheremenko, G., Lesesne, C., & Rasberry, C. N. (2020). Sexual Behaviors, Referral to Sexual Health Services, and Use of Sexual Health Services Among Transgender High School Students. *The Journal of school health*, *90*(5), 349-357. https://doi.org/10.1111/josh.12880  Appenroth, M. N., Koppe, U., Hickson, F., Schink, S., Hahne, A., Schmidt, A. J., Weatherburn, P., & Marcus, U. (2022). Sexual happiness and satisfaction with sexual safety among German trans men who have sex with men: results from EMIS-2017. *Journal of the International AIDS Society*, *25*, Article e25992. https://doi.org/10.1002/jia2.25992  Arayasirikul, S., Turner, C. M., Hernandez, C. J., Trujillo, D., Fisher, M. R., & Wilson, E. C. (2021). Transphobic Adverse Childhood Experiences as a Determinant of Mental and Sexual Health for Young Trans Women in the San Francisco Bay Area. *Transgender Health*. https://doi.org/10.1089/trgh.2021.0062  Arora, T., Wadhwa, N., Pandhi, D., Diwaker, P., & Arora, V. K. (2021). Transgenders are the most vulnerable amongst individuals engaging in receptive anal intercourse: A cross-sectional study from North India. *Journal of family medicine and primary care*, *10*(12), 4463-4470. https://doi.org/10.4103/jfmpc.jfmpc_634_21  Arrington-Sanders, R., Alvarenga, A., Galai, N., Arscott, J., Wirtz, A., Carr, R., Lopez, A., Beyrer, C., Nessen, R., & Celentano, D. (2022). Social determinants of transactional sex in a sample of young Black and Latinx sexual minority cisgender men and transgender women. *Journal of Adolescent Health*, *70*(2), 275-281. https://doi.org/10.1016/j.jadohealth.2021.08.002  Arseneault, L., Brassard, A., Lefebvre, A. A., Lafontaine, M. F., Godbout, N., Daspe, M. E., Savard, C., & Péloquin, K. (2023). Romantic Attachment and Intimate Partner Violence Perpetrated by Individuals Seeking Help: The Roles of Dysfunctional Communication Patterns and Relationship Satisfaction. *JOURNAL OF FAMILY VIOLENCE*. https://doi.org/10.1007/s10896-023-00600-z  Asadpour, E., Behzadipuor, S., & Zarenejad, M. (2019). Comparing sexual satisfaction and function in operated vs non-operated patients of gender identity disorder. *Journal of Practice in Clinical Psychology*, *7*(1), 71-78. https://doi.org/10.32598/jpcp.7.1.71  Atteberry-Ash, B., Kattari, S. K., Harner, V., Prince, D. M., Verdino, A. P., Kattari, L., & Park, I. Y. (2021). Differential Experiences of Mental Health among Transgender and Gender-Diverse Youth in Colorado. *Behavioral sciences (Basel, Switzerland)*, *11*(4). https://doi.org/10.3390/bs11040048  Auer, M. K., Fuss, J., Höhne, N., Stalla, G. K., & Sievers, C. (2014). Transgender transitioning and change of self-reported sexual orientation. *PloS one*, *9*(10), e110016. https://doi.org/10.1371/journal.pone.0110016  Ballard, Z. B., & Oswald, D. L. (2022). Sexual minority behavioral health outcomes: The role of identity authenticity and self-regulation. *Psychology of Sexual Orientation and Gender Diversity*. https://doi.org/10.1037/sgd0000578  Barrington, C., Wejnert, C., Guardado, M. E., Nieto, A. I., & Bailey, G. P. (2012). Social network characteristics and HIV vulnerability among transgender persons in San Salvador: Identifying opportunities for HIV prevention strategies. *AIDS and Behavior*, *16*(1), 214-224. https://doi.org/10.1007/s10461-011-9959-1  Bartolucci, C., Gómez-Gil, E., Salamero, M., Esteva, I., Guillamón, A., Zubiaurre, L., Molero, F., & Montejo, A. L. (2015). Sexual quality of life in gender-dysphoric adults before genital sex reassignment surgery. *Journal of Sexual Medicine*, *12*(1), 180-188. https://doi.org/10.1111/jsm.12758  Bauer, G. R., Redman, N., Bradley, K., & Scheim, A. I. (2013). Sexual health of trans men who are gay, bisexual, or who have sex with men: Results from Ontario, Canada. *International Journal of Transgenderism*, *14*(2), 66-74. https://doi.org/10.1080/15532739.2013.791650  Bauer, G. R., Travers, R., Scanlon, K., & Coleman, T. A. (2012). High heterogeneity of HIV-related sexual risk among transgender people in Ontario, Canada: a province-wide respondent-driven sampling survey. *BMC public health*, *12*, 292. https://doi.org/10.1186/1471-2458-12-292  Becker, I., Nieder, T. O., Cerwenka, S., Briken, P., Kreukels, B. P. C., Cohen-Kettenis, P. T., Cuypere, G., Haraldsen, I. R. H., & Richter-Appelt, H. (2016). Body image in young gender dysphoric adults: A European multi-center study. *Archives of Sexual Behavior*, *45*(3), 559-574. https://doi.org/10.1007/s10508-015-0527-z  Beckmeyer, J. J., Herbenick, D., & Eastman-Mueller, H. (2021). Sexual pleasure during college students' most recent partnered sexual experiences. *Journal of American college health : J of ACH*, 1-12. https://doi.org/10.1080/07448481.2021.1978461  Bellhouse, C., Walker, S., Fairley, C. K., Vodstrcil, L. A., Bradshaw, C. S., Chen, M. Y., & Chow, E. P. F. (2018). Patterns of sexual behaviour and sexual healthcare needs among transgender individuals in Melbourne, Australia, 2011-2014. *Sexually transmitted infections*, *94*(3), 212-215. https://doi.org/10.1136/sextrans-2016-052710  Bertrand, A. A., DeLong, M. R., McCleary, S. P., Nahabet, E. H., Slack, G. C., DaLio, A. L., Weimer, A. K., Kwan, L., Bernacki, J., Rudkin, G. H., & Plastic Surg Res, G. (2024). Gender-Affirming Mastectomy: Psychosocial and Surgical Outcomes in Transgender Adults. *JOURNAL OF THE AMERICAN COLLEGE OF SURGEONS*, *238*(5), 890-899. https://doi.org/10.1097/XCS.0000000000000940  Blair, C. S., Segura, E. R., Perez-Brumer, A. G., Sanchez, J., Lama, J. R., & Clark, J. L. (2016). Sexual orientation, gender identity and perceived source of infection among men who have sex with men (MSM) and transgender women (TW) recently diagnosed with HIV and/or STI in Lima, Peru. *AIDS and Behavior*, *20*(10), 2178-2185. https://doi.org/10.1007/s10461-015-1276-7  Blais, M., Aghedu, F. C., Ashley, F., Samoilenko, M., Chamberland, L., & Côté, I. (2022). Sexual orientation and gender identity and expression conversion exposure and their correlates among LGBTQI2+persons in Quebec, Canada. *PloS one*, *17*(4), Article e0265580. https://doi.org/10.1371/journal.pone.0265580  Blasdel, G., Kloer, C., Parker, A., Castle, E., Bluebond-Langner, R., & Zhao, L. C. (2022). Coming soon: Ability to orgasm after gender affirming vaginoplasty. *Journal of Sexual Medicine*, *19*(5), 781-788. https://doi.org/10.1016/j.jsxm.2022.02.015  Blosnich, J. R., Gordon, A. J., & Fine, M. J. (2015). Associations of sexual and gender minority status with health indicators, health risk factors, and social stressors in a national sample of young adults with military experience. *Annals of epidemiology*, *25*(9), 661-667. https://doi.org/10.1016/j.annepidem.2015.06.001  Boskey, E. R., Jolly, D., Mehra, G., & Ganor, O. (2022). Feasibility of an External Erectile Prosthesis for Transgender Men Who have Undergone Phalloplasty. *Sexual medicine*, *10*(5), 100560. https://doi.org/10.1016/j.esxm.2022.100560  Bothwell, S. J., Lawlace, M., Newcomb, M. E., & Whitton, S. W. (2024). Relationship quality, COVID stress, and mental health in sexual and gender minority young adults. *Journal of Social and Personal Relationships*, *41*(4), 912-930. https://doi.org/10.1177/02654075231217390  Bouman, M.-B., van der Sluis, W. B., van Woudenberg Hamstra, L. E., Buncamper, M. E., Kreukels, B. P. C., Meijerink, W. J. H. J., & Mullender, M. G. (2016). Patient-reported esthetic and functional outcomes of primary total laparoscopic intestinal vaginoplasty in transgender women with penoscrotal hypoplasia. *Journal of Sexual Medicine*, *13*(9), 1438-1444. https://doi.org/10.1016/j.jsxm.2016.06.009  Bourdon, J. L., Saunders, T. R., & Hancock, L. C. (2018). Acknowledgement and support matter: A brief report on gender identity and sexual orientation at a large, urban university. *Journal of American College Health*, *66*(8), 809-812. https://doi.org/10.1080/07448481.2018.1432627  Bowers, J. R., Branson, C. M., Fletcher, J. B., & Reback, C. J. (2012). Predictors of HIV sexual risk behavior among men who have sex with men, men who have sex with men and women, and transgender women. *International Journal of Sexual Health*, *24*(4), 290-302. https://doi.org/10.1080/19317611.2012.715120  Boyacioglu, N. E., Dinc, H., Ozcan, N. K., & Sahin, A. B. (2020). LGBT+ Individuals' Sexual and Mental Health: A Comparison with Hetereosexual Group. *Cyprus Journal of Medical Sciences*, *5*(3), 189-195. https://doi.org/10.5152/cjms.2020.864  Boyer, S. J., & Lorenz, T. K. (2020). The impact of heteronormative ideals imposition on sexual orientation questioning distress. *Psychology of Sexual Orientation and Gender Diversity*, *7*(1), 91-100. https://doi.org/10.1037/sgd0000352  Brooks, T. R., Bennett, T. N., Myhre, A., Plante, C. N., Reysen, S., Roberts, S. E., & Gerbasi, K. C. (2024). 'Chasing Tail': Testing the relative strength of sexual interest and social interaction as predictors of furry identity. *Journal of Sex Research*, *61*(2), 324-335. https://doi.org/10.1080/00224499.2022.2068180  Brown, C., Eisenberg, M. E., McMorris, B. J., & Sieving, R. E. (2020). Parents Matter: Associations Between Parent Connectedness and Sexual Health Indicators Among Transgender and Gender-Diverse Adolescents. *Perspectives on sexual and reproductive health*, *52*(4), 265-273. https://doi.org/10.1363/psrh.12168  Brown, J. R., Reid, D., Howarth, A. R., Mohammed, H., Saunders, J., Pulford, C. V., Ogaz, D., Hughes, G., & Mercer, C. H. (2023). Difficulty accessing condoms because of the COVID-19 pandemic reported by gay, bisexual and other men who have sex with men in the UK: findings from a large, cross-sectional, online survey. *International journal of STD & AIDS*, *34*(8), 541-547. https://doi.org/10.1177/09564624231160804  Budhwani, H., Hearld, K. R., Hasbun, J., Charow, R., Rosario, S., Tillotson, L., McGlaughlin, E., & Waters, J. (2017). Transgender female sex workers' HIV knowledge, experienced stigma, and condom use in the Dominican Republic. *PloS one*, *12*(11), e0186457. https://doi.org/10.1371/journal.pone.0186457  Buncamper, M. E., Honselaar, J. S., Bouman, M. B., Özer, M., Kreukels, B. P. C., & Mullender, M. G. (2015). Aesthetic and functional outcomes of neovaginoplasty using penile skin in male‐to‐female transsexuals. *Journal of Sexual Medicine*, *12*(7), 1626-1634. https://doi.org/10.1111/jsm.12914  Buncamper, M. E., van der Sluis, W. B., de Vries, M., Witte, B. I., Bouman, M.-B., & Mullender, M. G. (2017). Penile Inversion Vaginoplasty with or without Additional Full-Thickness Skin Graft: To Graft or Not to Graft? *Plastic and reconstructive surgery*, *139*(3), 649e-656e. https://doi.org/10.1097/PRS.0000000000003108  Bungener, S. L., de Vries, A. L. C., Popma, A., & Steensma, T. D. (2020). Sexual experiences of young transgender persons during and after gender-affirmative treatment. *Pediatrics*, *146*(6). https://doi.org/10.1542/peds.2019-1411  Butcher, R. L., Kinney, L. M., Blasdel, G. P., Elwyn, G., Myers, J. B., Boh, B., Luck, K. M., & Moses, R. A. (2023). Decision making in metoidioplasty and phalloplasty gender-affirming surgery: a mixed methods study. *Journal of Sexual Medicine*, *20*(7), 1032-1043. https://doi.org/10.1093/jsxmed/qdad063  Cai, X., Fisher, C. B., Alohan, D., Tellone, S., Grov, C., Cohall, A., & Meunier, É. (2023). Sexual and Gender Minority Individuals' Interest in Sexual Health Services at Collective Sex Venues in New York City. *AIDS and Behavior*, *27*(3), 761-771. https://doi.org/10.1007/s10461-022-03808-0  Canale, D., Molinaro, A., Marcocci, C., Morelli, G., Matteucci, V., Mollaioli, D., Jannini, E. A., & Sartucci, F. (2022). Genital Sensitivity and Perceived Orgasmic Intensity in Transgender Women With Gender Dysphoria After Gender-Affirming Surgery: A Pilot Study Comparing Pelvic Floor Evoked Somatosensory Potentials and Patient Subjective Experience. *The journal of sexual medicine*, *19*(9), 1479-1487. https://doi.org/10.1016/j.jsxm.2022.06.002  Carvalho, S. A., Lapa, T., & Pascoal, P. M. (2024). The Need to Look at Transgender and Gender Diverse People's Health: A Preliminary Descriptive Report on Pain, Sexual Distress, and Health Profile of Five Transmasculine People and One Non-Binary Person with Endometriosis. *HEALTHCARE*, *12*(12), Article 1229. https://doi.org/10.3390/healthcare12121229  Catelan, R. F., Saadeh, A., Lobato, M. I. R., Gagliotti, D. A. M., Nardi, H. C., & Costa, A. B. (2022). Depression, self-esteem, and resilience and its relationship with psychological features of sexuality among transgender men and women from brazil. *Archives of Sexual Behavior*. https://doi.org/10.1007/s10508-021-02189-0  Cerwenka, S., Nieder, T. O., Briken, P., Cohen-Kettenis, P. T., De Cuypere, G., Haraldsen, I. R. H., Kreukels, B. P. C., & Richter-Appelt, H. (2014). Intimate partnerships and sexual health in gender-dysphoric individuals before the start of medical treatment. *International Journal of Sexual Health*, *26*(1), 52-65. https://doi.org/10.1080/19317611.2013.829153  Cerwenka, S., Nieder, T. O., Cohen-Kettenis, P., De Cuypere, G., Haraldsen, I. R. H., Kreukels, B. P. C., & Richter-Appelt, H. (2014). Sexual behavior of gender-dysphoric individuals before gender-confirming interventions: A European multicenter study. *Journal of Sex & Marital Therapy*, *40*(5), 457-471. https://doi.org/10.1080/0092623X.2013.772550  Chadwick, S. B., & van Anders, S. M. (2022). Orgasm Coercion and Negative Relationship and Psychological Outcomes: The Role of Gender, Sexual Identity, Perpetration Tactics, and Perceptions of the Perpetrator's Intentions. *Archives of Sexual Behavior*, *51*(1), 653-671. https://doi.org/10.1007/s10508-021-02162-x  Chadwick, S. B., & van Anders, S. M. (2022). Orgasm Coercion: Overlaps Between Pressuring Someone to Orgasm and Sexual Coercion. *Archives of Sexual Behavior*, *51*(1), 633-651. https://doi.org/10.1007/s10508-021-02156-9  Charest, M., & Kleinplatz, P. J. (2022). What Do Young, Canadian, Straight and LGBTQ Men and Women Learn About Sex and from Whom? *Sexuality Research and Social Policy*, *19*(2), 622-637. https://doi.org/10.1007/s13178-021-00578-7  Charest, M., Kleinplatz, P. J., & Lund, J. I. (2016). Sexual health information disparities between heterosexual and LGBTQ+ young adults: Implications for sexual health. *Canadian Journal of Human Sexuality*, *25*(2), 74-85. https://doi.org/10.3138/cjhs.252-A9  Chen, E. M. Y., Hollowell, A., Truong, T., Bentley-Edwards, K., Myers, E., Erkanli, A., Holt, L., & Swartz, J. J. (2023). Contraceptive Access and Use Among Undergraduate and Graduate Students During COVID-19: Online Survey Study. *JMIR formative research*, *7*. https://doi.org/10.2196/38491  Chow, J. Y., Konda, K. A., Calvo, G. M., Klausner, J. D., & Cáceres, C. F. (2017). Demographics, Behaviors, and Sexual Health Characteristics of High Risk Men Who Have Sex With Men and Transgender Women Who Use Social Media to Meet Sex Partners in Lima, Peru. *Sexually transmitted diseases*, *44*(3), 143-148. https://doi.org/10.1097/OLQ.0000000000000566  Colson, P. W., Franks, J., Wu, Y., Winterhalter, F. S., Knox, J., Ortega, H., El-Sadr, W. M., & Hirsch-Moverman, Y. (2020). Adherence to pre-exposure prophylaxis in black men who have sex with men and transgender women in a community setting in Harlem, NY. *AIDS and Behavior*, *24*(12), 3436-3455. https://doi.org/10.1007/s10461-020-02901-6  Comulada, W. S., Step, M., Fletcher, J. B., Tanner, A. E., Dowshen, N. L., Arayasirikul, S., Baker, K. K., Zuniga, J., Swendeman, D., Medich, M., Kao, U. H., Northrup, A., Nieto, O., & Brooks, R. A. (2020). Predictors of internet health information–seeking behaviors among young adults living with HIV across the United States: Longitudinal observational study. *Journal of Medical Internet Research*, *22*(11). https://doi.org/10.2196/18309  Costantino, A., Cerpolini, S., Alvisi, S., Morselli, P. G., Venturoli, S., & Meriggiola, M. C. (2013). A prospective study on sexual function and mood in female-to-male transsexuals during testosterone administration and after sex reassignment surgery. *Journal of Sex & Marital Therapy*, *39*(4), 321-335. https://doi.org/10.1080/0092623X.2012.736920  Craig, A., Walsh, J., & Quinn, K. (2024). Intersectional microaggressions, sexual identity concealment, and mental health of young Black sexual minority men and transgender women. *Archives of Sexual Behavior*, *53*(4), 1245-1254. https://doi.org/10.1007/s10508-023-02777-2  Crissman, H. P., Haley, C., Stroumsa, D., Tilea, A., Moravek, M. B., Harris, L. H., & Dalton, V. K. (2022). Leveraging administrative claims to understand disparities in gender minority health: Contraceptive use patterns among transgender and nonbinary people. *LGBT Health*, *9*(3), 186-193. https://doi.org/10.1089/lgbt.2021.0303  Cuq, J., Jurek, L., Morel-Journel, N., Oriol, S., & Neuville, P. (2023). Gynecological primary care of trans men and transmasculine non-binary individuals, a French descriptive study. *International Journal of Transgender Health*. https://doi.org/10.1080/26895269.2023.2283529  Daans, C. G., Hoornenborg, E., de Haseth, K. B., Ozer, M., Bouman, M. B., Conemans, E., Kreukels, B. P. C., den Heijer, M., & van der Sluis, W. B. (2022). HIV Prevalence and High-Risk Subgroup Identification in Transgender Women Who Undergo Primary Vaginoplasty in the Netherlands. *Transgender Health*. https://doi.org/10.1089/trgh.2021.0059  Dadasovich, R., Auerswald, C., Minnis, A. M., Raymond, H. F., McFarland, W., & Wilson, E. C. (2017). Testosterone and sexual risk among transmen: A mixed methods exploratory study. *Culture, Health & Sexuality*, *19*(2), 256-266. https://doi.org/10.1080/13691058.2016.1216605  Dangerfield, D. T., II, Johnson, D., Hamlin-Palmer, S., Browne, D. C., Mayer, K. H., & Hickson, D. A. (2020). Prevalence and correlates of rectal douching and enema use among Black sexual minority men and Black transwomen in the Deep South. *Archives of Sexual Behavior*, *49*(6), 1915-1922. https://doi.org/10.1007/s10508-019-01605-w  Day, S., Smith, J., Perera, S., Jones, S., & Kinsella, R. (2021). Beyond the binary: sexual health outcomes of transgender and non-binary service users of an online sexual health service. *International journal of STD & AIDS*, *32*(10), 896-902. https://doi.org/10.1177/0956462420982830  de la Court, F., van Wees, D., van Benthem, B., Hoornenborg, E., Prins, M., & Boyd, A. (2023). Characterizing subgroups of sexual behaviors among men who have sex with men eligible for, but not using, PrEP in the Netherlands. *PloS one*, *18*(4), Article e0284056. https://doi.org/10.1371/journal.pone.0284056  de Menezes Gomes, R., de Araújo Lopes, F., & Castro, F. N. (2020). Influence of Sexual Genotype and Gender Self-Perception on Sociosexuality and Self-Esteem among Transgender People. *Human nature (Hawthorne, N.Y.)*, *31*(4), 483-496. https://doi.org/10.1007/s12110-020-09381-6  De Moissac, D., Prada, K., Gueye, N. R., Avanthay-Strus, J., & Hardy, S. (2024). Healthcare Service Utilization and Perceived Gaps: The Experience of French-Speaking 2S/LGBTQI+ People in Manitoba. *Healthcare policy = Politiques de sante*, *19*(3), 62-77. https://doi.org/10.12927/hcpol.2024.27239  de Rooij, F. P. W., van de Grift, T. C., Veerman, H., Al-Tamimi, M., van der Sluis, W. B., Ronkes, B. L., Özer, M., Mullender, M. G., Bouman, M.-B., & Pigot, G. L. S. (2021). Patient-reported outcomes after genital gender-affirming surgery with versus without urethral lengthening in transgender men. *Journal of Sexual Medicine*, *18*(5), 974-981. https://doi.org/10.1016/j.jsxm.2021.03.002  Defreyne, J., Elaut, E., Den Heijer, M., Kreukels, B., Fisher, A. D., & T'Sjoen, G. (2021). Sexual orientation in transgender individuals: results from the longitudinal ENIGI study. *International Journal of Impotence Research*, *33*(7), 694-702. https://doi.org/10.1038/s41443-020-00402-7  Defreyne, J., Elaut, E., Kreukels, B., Fisher, A. D., Castellini, G., Staphorsius, A., Den Heijer, M., Heylens, G., & T'Sjoen, G. (2020). Sexual Desire Changes in Transgender Individuals Upon Initiation of Hormone Treatment: Results From the Longitudinal European Network for the Investigation of Gender Incongruence. *The journal of sexual medicine*, *17*(4), 812-825. https://doi.org/10.1016/j.jsxm.2019.12.020  Delgado, J. R., Segura, E. R., Lake, J. E., Sanchez, J., Lama, J. R., & Clark, J. L. (2017). Event-level analysis of alcohol consumption and condom use in partnership contexts among men who have sex with men and transgender women in Lima, Peru. *Drug and Alcohol Dependence*, *170*, 17-24. https://doi.org/10.1016/j.drugalcdep.2016.10.033  Deutsch, M. B., Reisner, S. L., Peitzmeier, S., Potter, J., Pardee, D., & Hughto, J. M. W. (2020). Recent Penile Sexual Contact Is Associated With an Increased Odds of High-Risk Cervical Human Papillomavirus Infection in Transgender Men. *Sexually transmitted diseases*, *47*(1), 48-53. https://doi.org/10.1097/OLQ.0000000000001072  Dharma, C., Scheim, A. I., & Bauer, G. R. (2019). Exploratory factor analysis of two sexual health scales for transgender people: Trans-Specific Condom/Barrier Negotiation Self-Efficacy (T-Barrier) and Trans-Specific Sexual Body Image Worries (T-Worries). *Archives of Sexual Behavior*, *48*(5), 1563-1572. https://doi.org/10.1007/s10508-018-1383-4  Djordjevic, M. L., Bizic, M. R., Duisin, D., Bouman, M.-B., & Buncamper, M. (2016). Reversal surgery in regretful male-to-female transsexuals after sex reassignment surgery. *Journal of Sexual Medicine*, *13*(6), 1000-1007. https://doi.org/10.1016/j.jsxm.2016.02.173  Döring, N., Mohseni, M. R., Pietras, L., Dekker, A., & Briken, P. (2024). Research in brief: How prevalent is rough sex? Results from a national online sample of adults in Germany. *Perspectives on sexual and reproductive health*, *56*(2), 90-97. https://doi.org/10.1111/psrh.12267  Dorrell, K. D., Benjamin, I., Dyar, C., Davila, J., & Feinstein, B. A. (2024). Minority Stress and Relationship Satisfaction Among Bi plus Individuals: The Roles of Partner Gender and Sexual Orientation. *Psychology of Sexual Orientation and Gender Diversity*. https://doi.org/10.1037/sgd0000711  Dubé, S., Santaguida, M., Zhu, C. Y., Di Tomasso, S., Hu, R., Cormier, G., Johnson, A. P., & Vachon, D. (2022). Sex robots and personality: It is more about sex than robots. *COMPUTERS IN HUMAN BEHAVIOR*, *136*, Article 107403. https://doi.org/10.1016/j.chb.2022.107403  Dyar, C., Feinstein, B. A., Zimmerman, A. R., Newcomb, M. E., Mustanski, B., & Whitton, S. W. (2020). Dimensions of sexual orientation and rates of intimate partner violence among young sexual minority individuals assigned female at birth: The role of perceived partner jealousy. *Psychology of Violence*, *10*(4), 411-421. https://doi.org/10.1037/vio0000275  Dyar, C., Newcomb, M. E., Mustanski, B., & Whitton, S. W. (2020). A structural equation model of sexual satisfaction and relationship functioning among sexual and gender minority individuals assigned female at birth in diverse relationships. *Archives of Sexual Behavior*, *49*(2), 693-710. https://doi.org/10.1007/s10508-019-1403-z  Eaton, L. A., Kalichman, S. C., Price, D., Finneran, S., Allen, A., & Maksut, J. (2017). Stigma and conspiracy beliefs related to pre-exposure prophylaxis (PrEP) and interest in using PrEP among black and white men and transgender women who have sex with men. *AIDS and Behavior*, *21*(5), 1236-1246. https://doi.org/10.1007/s10461-017-1690-0  Edge, J., & Vonk, J. (2024). A comparison of mate preferences in asexual and allosexual adults. *Archives of Sexual Behavior*, *53*(1), 17-24. https://doi.org/10.1007/s10508-023-02723-2  Ejaz, M., Andersson, S., Batool, S., Ali, T., & Ekström, A. M. (2021). Anal human papillomavirus infection among men who have sex with men and transgender women living with and without HIV in Pakistan: findings from a cross-sectional study. *BMJ open*, *11*(11), e052176. https://doi.org/10.1136/bmjopen-2021-052176  Elfering, L., van de Grift, T. C., Al-Tamimi, M., Timmermans, F. W., de Haseth, K. B., Pigot, G. L. S., Lissenberg-Witte, B. I., Bouman, M.-B., & Mullender, M. G. (2021). How Sensitive Is the Neophallus? Postphalloplasty Experienced and Objective Sensitivity in Transmasculine Persons. *Sexual medicine*, *9*(5), 100413. https://doi.org/10.1016/j.esxm.2021.100413  Fein, L. A., Salgado, C. J., Sputova, K., Estes, C. M., & Medina, C. A. (2018). Sexual preferences and partnerships of transgender persons mid- or post-transition. *Journal of Homosexuality*, *65*(5), 659-671. https://doi.org/10.1080/00918369.2017.1333808  Feldman, J., Romine, R. S., & Bockting, W. O. (2014). HIV risk behaviors in the US transgender population: Prevalence and predictors in a large Internet sample. *Journal of Homosexuality*, *61*(11), 1558-1588. https://doi.org/10.1080/00918369.2014.944048  Ferlatte, O., Panwala, V., Rich, A. J., Scheim, A. I., Blackwell, E., Scott, K., Salway, T., & Knight, R. (2020). Identifying health differences between transgender and cisgender gay, bisexual and other men who have sex with men using a community-based approach. *Journal of Sex Research*, *57*(8), 1005-1013. https://doi.org/10.1080/00224499.2020.1740148  Fornander, M. J., Egan, A. M., Robertson, G. C., & Moser, C. N. (2024). Self-Reported Sexual Behavior of Transgender Youth. *JOURNAL OF PEDIATRIC AND ADOLESCENT GYNECOLOGY*, *37*(3), 336-341. https://doi.org/10.1016/j.jpag.2023.12.003  Gaither, T. W., Awad, M. A., Osterberg, E. C., Romero, A., Bowers, M. L., & Breyer, B. N. (2017). Impact of sexual orientation identity on medical morbidities in male-to-female transgender patients. *LGBT Health*, *4*(1), 11-16. https://doi.org/10.1089/lgbt.2016.0097  Galea, J. T., Kinsler, J. J., McLean, S., Calvo, G., Sánchez, H., Leon, S. R., & Brown, B. (2016). Rectal douching prevalence and practices among Peruvian men who have sex with men and transwomen: Implications for rectal microbicides. *AIDS and Behavior*, *20*(11), 2555-2564. https://doi.org/10.1007/s10461-015-1221-9  Galupo, M. P., Mitchell, R. C., & Davis, K. S. (2015). Sexual minority self-identification: Multiple identities and complexity. *Psychology of Sexual Orientation and Gender Diversity*, *2*(4), 355-364. https://doi.org/10.1037/sgd0000131  Gamarel, K. E., Reisner, S. L., Laurenceau, J.-P., Nemoto, T., & Operario, D. (2014). Gender minority stress, mental health, and relationship quality: A dyadic investigation of transgender women and their cisgender male partners. *Journal of Family Psychology*, *28*(4), 437-447. https://doi.org/10.1037/a0037171  Garcia, M. M., Christopher, N. A., De Luca, F., Spilotros, M., & Ralph, D. J. (2014). Overall satisfaction, sexual function, and the durability of neophallus dimensions following staged female to male genital gender confirming surgery: the Institute of Urology, London U.K. experience. *Translational andrology and urology*, *3*(2), 156-162. https://doi.org/10.3978/j.issn.2223-4683.2014.04.10  Gerassi, L. B., Cruys, C., Hendry, N., & Rosales, M. D. (2024). An Exploration of Sex Trading for Compensation and LGBTQ plus Inclusive Screening Practices: Perspectives of Young People who have Experienced Sex Trading and/or Homelessness. *CHILDREN AND YOUTH SERVICES REVIEW*, *156*, Article 107314. https://doi.org/10.1016/j.childyouth.2023.107314  Gerymski, R. (2021). Short Sexual Well-Being Scale - a cross-sectional validation among transgender and cisgender people. *Health Psychology Report*, *9*(3), 276-287. https://doi.org/10.5114/hpr.2021.102349  Gieles, N. C., van de Grift, T. C., Elaut, E., Heylens, G., Becker-Hebly, I., Nieder, T. O., Laan, E. T. M., & Kreukels, B. P. C. (2022). Pleasure please! Sexual pleasure and influencing factors in transgender persons: An ENIGI follow-up study. *International Journal of Transgender Health*. https://doi.org/10.1080/26895269.2022.2028693  Gil-Llario, M. D., Gil-Juliá, B., Giménez-García, C., Bergero-Miguel, T., & Ballester-Arnal, R. (2021). Sexual behavior and sexual health of transgender women and men before treatment: Similarities and differences. *International Journal of Transgender Health*, *22*(3), 304-315. https://doi.org/10.1080/26895269.2020.1838386  Godfrey, L. M., Whitton, S. W., Dyar, C., Newcomb, M. E., & Mustanski, B. (2021). Sexual agreements among young sexual and gender minorities assigned male at birth: Associations with relationship quality and break-up. *Archives of Sexual Behavior*, *50*(3), 1035-1045. https://doi.org/10.1007/s10508-020-01781-0  Goldey, K. L., Cital, M. N., Rodriguez, S. C., Espinosa, A., & Barton, E. A. (2022). Desire on Lockdown? Sexual Desire and COVID-19 Stress Among LGBTQ plus and Cisgender, Heterosexual College Students. *Psychology of Sexual Orientation and Gender Diversity*. https://doi.org/10.1037/sgd0000596  Golub, S. A., Fikslin, R. A., Starbuck, L., & Klein, A. (2019). High Rates of PrEP Eligibility but Low Rates of PrEP Access Among a National Sample of Transmasculine Individuals. *Journal of acquired immune deficiency syndromes (1999)*, *82*(1), e1-e7. https://doi.org/10.1097/QAI.0000000000002116  Gonzalez, C. A., Gallego, J. D., & Bockting, W. O. (2017). Demographic characteristics, components of sexuality and gender, and minority stress and their associations to excessive alcohol, cannabis, and illicit (noncannabis) drug use among a large sample of transgender people in the United States. *The Journal of Primary Prevention*, *38*(4), 419-445. https://doi.org/10.1007/s10935-017-0469-4  Griffin, M., Jaiswal, J., Martino, R. J., LoSchiavo, C., Comer-Carruthers, C., Krause, K. D., Stults, C. B., & Halkitis, P. N. (2022). Sex in the time of covid-19: Patterns of sexual behavior among lgbtq+ individuals in the uS. *Archives of Sexual Behavior*. https://doi.org/10.1007/s10508-022-02298-4  Grov, C., Zohra, F., Westmoreland, D. A., Mirzayi, C., D'Angelo, A., Stief, M., Kulkarni, S., Nash, D., & Carrico, A. W. (2022). Sex in the Era of COVID-19 in a U.S. National Cohort of Cisgender Men, Transgender Women, and Transgender Men Who Have Sex with Men: April-May 2020. *Archives of Sexual Behavior*, *51*(1), 343-354. https://doi.org/10.1007/s10508-021-02121-6  Gupta, R., & Gupta, R. (2022). Achieving Correct Axis and Good Depth in Gender Affirming Vaginoplasties by Penile-Perineoscrotal Flap Vaginoplasty. *INDIAN JOURNAL OF PLASTIC SURGERY*, *55*(02), 188-195. https://doi.org/10.1055/s-0041-1740530  Haas, S. M., & Lannutti, P. J. (2022). Relationship maintenance behaviors, resilience, and relational quality in romantic relationships of LGBTQ+ people. *Couple and Family Psychology: Research and Practice*, *11*(2), 117-131. https://doi.org/10.1037/cfp0000186 (Risk and Resilience in Sexual and Gender Minority Relationships: From Theory to Practice)  Herrera, M. C., Konda, K. A., Leon, S. R., Deiss, R., Brown, B., Calvo, G. M., Salvatierra, H. J., Caceres, C. F., & Klausner, J. D. (2016). Impact of alcohol use on sexual behavior among men who have sex with men and transgender women in Lima, Peru. *Drug and Alcohol Dependence*, *161*, 147-154. https://doi.org/10.1016/j.drugalcdep.2016.01.030  Hess, J., Henkel, A., Bohr, J., Rehme, C., Panic, A., Panic, L., Rossi Neto, R., Hadaschik, B., & Hess, Y. (2018). Sexuality after Male-to-Female Gender Affirmation Surgery. *BioMed research international*, *2018*, 9037979. https://doi.org/10.1155/2018/9037979  Hess, J., Hess-Busch, Y., Kronier, J., Rübben, H., & Rossi Neto, R. (2016). Modified Preparation of the Neurovascular Bundle in Male to Female Transgender Patients. *Urologia internationalis*, *96*(3), 354-359. https://doi.org/10.1159/000443281  Hickson, F., Appenroth, M., Koppe, U., Schmidt, A. J., Reid, D., & Weatherburn, P. (2020). Sexual and Mental Health Inequalities across Gender Identity and Sex-Assigned-at-Birth among Men-Who-Have-Sex-with-Men in Europe: Findings from EMIS-2017. *International journal of environmental research and public health*, *17*(20). https://doi.org/10.3390/ijerph17207379  Hiransuthikul, A., Janamnuaysook, R., Himma, L., Taya, C., Amatsombat, T., Chumnanwet, P., Samitpol, K., Chancham, A., Kongkapan, J., Rueannak, J., Getwongsa, P., Srimanus, P., Teeratakulpisarn, N., Thammajaruk, N., Avery, M., Wansom, T., Mills, S., Ramautarsing, R. A., & Phanuphak, N. (2021). Acceptability and satisfaction towards self-collection for chlamydia and gonorrhoea testing among transgender women in Tangerine Clinic, Thailand: shifting towards the new normal. *Journal of the International AIDS Society*, *24*(9), e25801. https://doi.org/10.1002/jia2.25801  Hiransuthikul, A., Trachunthong, D., Pattanachaiwit, S., Teeratakulpisarn, N., Chamnan, P., Pathipvanich, P., Thongpaen, S., Pengnonyang, S., Nonenoy, S., Lertpiriyasuwat, C., & Phanuphak, P. (2019). Changes in risk behaviors among Thai men who have sex with men and transgender women enrolled in the test and treat cohort. *AIDS Care*, *31*(9), 1178-1183. https://doi.org/10.1080/09540121.2019.1580346  Hoagland, B., De Boni, R. B., Moreira, R. I., Madruga, J. V., Kallas, E. G., Goulart, S. P., Cerqueira, N., Torres, T. S., Luz, P. M., Fernandes, N. M., Liu, A. Y., Grinsztejn, B., & Veloso, V. G. (2017). Awareness and willingness to use pre-exposure prophylaxis (PrEP) among men who have sex with men and transgender women in Brazil. *AIDS and Behavior*, *21*(5), 1278-1287. https://doi.org/10.1007/s10461-016-1516-5  Huynh, L., Gysler, M., Loutfy, M. R., Margolese, S., Yudin, M. H., Conway, T., Maxwell, J., Muchenje, M., O'Brien-Teengs, D., Shapiro, H., & Librach, C. (2012). Access to conception planning information and services for people living with HIV in Ontario, Canada: A community-based research study. *Vulnerable Children and Youth Studies*, *7*(1), 6-19. https://doi.org/10.1080/17450128.2011.635723  Ikeda, J. M., Racancoj, O., Welty, S., Page, K., Hearst, N., & McFarland, W. (2018). Risk behaviors and perceptions among self-identified men who have sex with men (MSM), bisexuals, transvestites, and transgender women in western Guatemala. *AIDS and Behavior*, *22*(Suppl 1), S45-S56. https://doi.org/10.1007/s10461-018-2190-6  Jabs, F., & Brotto, L. A. (2024). Examining the Treatment Relevance of the Approach-Avoidance Motivation Model for Sexual Interest/Arousal Disorder in Women and Non-Binary Individuals. *Journal of Sex Research*, *61*(4), 562-573. https://doi.org/10.1080/00224499.2022.2148240  Jacobsson, J., Andréasson, M., Kölby, L., Elander, A., & Selvaggi, G. (2017). Patients' Priorities Regarding Female-to-Male Gender Affirmation Surgery of the Genitalia-A Pilot Study of 47 Patients in Sweden. *The journal of sexual medicine*, *14*(6), 857-864. https://doi.org/10.1016/j.jsxm.2017.04.005  Janulis, P., Neray, B., Birkett, M., Phillips, G., II, & Mustanski, B. (2020). No evidence of bias in sexual partnership corroboration by race and ethnicity among a diverse cohort of young men who have sex with men and transgender women. *Archives of Sexual Behavior*, *49*(1), 267-274. https://doi.org/10.1007/s10508-019-1455-0  Jerome, R. R., Randhawa, M. K., Kowalczyk, J., Sinclair, A., & Monga, I. (2022). Sexual Satisfaction After Gender Affirmation Surgery in Transgender Individuals. *Cureus*, *14*(7), e27365. https://doi.org/10.7759/cureus.27365  Johnston, L. G., Vaillant, T. C., Dolores, Y., & Vales, H. M. (2013). HIV, hepatitis B/C and syphilis prevalence and risk behaviors among gay, transsexuals and men who have sex with men, Dominican Republic. *International journal of STD & AIDS*, *24*(4), 313-321. https://doi.org/10.1177/0956462412472460  Kaida, A., Carter, A., de Pokomandy, A., Patterson, S., Proulx-Boucher, K., Nohpal, A., Sereda, P., Colley, G., O'Brien, N., Thomas-Pavanel, J., Beaver, K., Nicholson, V. J., Tharao, W., Fernet, M., Otis, J., Hogg, R. S., & Loutfy, M. (2015). Sexual inactivity and sexual satisfaction among women living with HIV in Canada in the context of growing social, legal and public health surveillance. *Journal of the International AIDS Society*, *18*(Suppl 5), 20284. https://doi.org/10.7448/IAS.18.6.20284  Kanhai, R. C. J. (2016). Sensate Vagina Pedicled-Spot for Male-to-Female Transsexuals: The Experience in the First 50 Patients. *Aesthetic plastic surgery*, *40*(2), 284-287. https://doi.org/10.1007/s00266-016-0620-2  Kanlagna, A., Oillic, J., Verdier, J., Perrot, P., & Lancien, U. (2024). Prospective assessment of the quality of life and nipple sensation after gender-affirming chest surgery. *Journal of plastic, reconstructive & aesthetic surgery : JPRAS*, *94*, 46-49. https://doi.org/10.1016/j.bjps.2024.05.022  Kaplan, R. L., McGowan, J., & Wagner, G. J. (2016). HIV prevalence and demographic determinants of condomless receptive anal intercourse among trans feminine individuals in Beirut, Lebanon. *Journal of the International AIDS Society*, *19*(3 Suppl 2), 20787. https://doi.org/10.7448/IAS.19.3.20787  Katz-Wise, S. L., Mereish, E. H., & Woulfe, J. (2017). Associations of bisexual-specific minority stress and health among cisgender and transgender adults with bisexual orientation. *Journal of Sex Research*, *54*(7), 899-910. https://doi.org/10.1080/00224499.2016.1236181  Katz-Wise, S. L., Ranker, L. R., Kraus, A. D., Wang, Y. C., Xuan, Z. M., Green, J. G., & Holt, M. (2023). Fluidity in Gender Identity and Sexual Orientation Identity in Transgender and Nonbinary Youth. *Journal of Sex Research*. https://doi.org/10.1080/00224499.2023.2244926  Katz-Wise, S. L., Reisner, S. L., White Hughto, J. M., & Budge, S. L. (2017). Self-reported changes in attractions and social determinants of mental health in transgender adults. *Archives of Sexual Behavior*, *46*(5), 1425-1439. https://doi.org/10.1007/s10508-016-0812-5  Katz-Wise, S. L., Williams, D. N., Keo-Meier, C. L., Budge, S. L., Pardo, S., & Sharp, C. (2017). Longitudinal associations of sexual fluidity and health in transgender men and cisgender women and men. *Psychology of Sexual Orientation and Gender Diversity*, *4*(4), 460-471. https://doi.org/10.1037/sgd0000246  Kennis, M., Duecker, F., Elaut, E., T'Sjoen, G., Loeys, T., Sack, A. T., & Dewitte, M. (2023). Daily Sexual Behavior, Sexual Esteem, and Body Image in Transgender and Cisgender Individuals. *Journal of Sex Research*, *60*(6), 859-867. https://doi.org/10.1080/00224499.2022.2158172  Kennis, M., Duecker, F., T'Sjoen, G., Sack, A. T., & Dewitte, M. (2022). Sexual Self-Concept Discrepancies Mediate the Relation between Gender Dysphoria Sexual Esteem and Sexual Attitudes in Binary Transgender Individuals. *Journal of Sex Research*, *59*(4), 524-536. https://doi.org/10.1080/00224499.2021.1951643  Keshinro, B., Crowell, T. A., Nowak, R. G., Adebajo, S., Peel, S., Gaydos, C. A., Rodriguez-Hart, C., Baral, S. D., Walsh, M. J., Njoku, O. S., Odeyemi, S., Ngo-Ndomb, T., Blattner, W. A., Robb, M. L., Charurat, M. E., & Ake, J. (2016). High prevalence of HIV, chlamydia and gonorrhoea among men who have sex with men and transgender women attending trusted community centres in Abuja and Lagos, Nigeria. *Journal of the International AIDS Society*, *19*(1), 21270. https://doi.org/10.7448/IAS.19.1.21270  Khorrami, A., Kumar, S., Bertin, E., Wassersug, R., O'Dwyer, C., Mukherjee, S., Witherspoon, L., Mankowski, P., Genoway, K., & Kavanagh, A. G. (2022). The Sexual Goals of Metoidioplasty Patients and Their Attitudes Toward Using PDE5 Inhibitors and Intracavernosal Injections as Erectile Aids. *Sexual medicine*, *10*(3), 100505. https://doi.org/10.1016/j.esxm.2022.100505  Kim, G.-W., & Jeong, G.-W. (2014). Neural mechanisms underlying sexual arousal in connection with sexual hormone levels: A comparative study of the postoperative male-to-female transsexuals and premenopausal and menopausal women. *NeuroReport: For Rapid Communication of Neuroscience Research*, *25*(9), 693-700. https://doi.org/10.1097/WNR.0000000000000159  Kim, G.-W., Kim, S.-K., & Jeong, G.-W. (2016). Neural activation-based sexual orientation and its correlation with free testosterone level in postoperative female-to-male transsexuals: preliminary study with 3.0-T fMRI. *Surgical and radiologic anatomy : SRA*, *38*(2), 245-252. https://doi.org/10.1007/s00276-015-1547-z  Kim, T. H., Kim, G. W., Kim, S. K., & Jeong, G. W. (2016). Brain activation-based sexual orientation in female-to-male transsexuals. *International Journal of Impotence Research*, *28*(1), 31-38. https://doi.org/10.1038/ijir.2015.29  Kimani, M., van der Elst, E. M., Chiro, O., Oduor, C., Wahome, E., Kazungu, W., Shally, M., Rinke de Wit, T. F., Graham, S. M., Operario, D., & Sanders, E. J. (2019). PrEP interest and HIV-1 incidence among MSM and transgender women in coastal Kenya. *Journal of the International AIDS Society*, *22*(6), e25323. https://doi.org/10.1002/jia2.25323  Kline, K., & Randall, A. K. (2021). The Moderating Effect of Internalized Transphobia on the Association Between Gender Congruence and Sexual Satisfaction for Transgender Men. *Journal of Lgbtq Issues in Counseling*, *15*(1), 93-109. https://doi.org/10.1080/15538605.2021.1868378  Kokogho, A., Amusu, S., Baral, S. D., Charurat, M. E., Adebajo, S., Makanjuola, O., Tonwe, V., Storme, C., Michael, N. L., Robb, M. L., Ake, J. A., Nowak, R. G., & Crowell, T. A. (2021). Disclosure of same-sex sexual practices to family and healthcare providers by men who have sex with men and transgender women in Nigeria. *Archives of Sexual Behavior*, *50*(4), 1665-1676. https://doi.org/10.1007/s10508-020-01644-8  Küenzlen, L., Nasim, S., van Neerven, S., Kühn, S., Burger, A. E., Sohn, M., Rieger, U. M., & Bozkurt, A. (2020). Multimodal Evaluation of Functional Nerve Regeneration in Transgender Individuals After Phalloplasty With a Free Radial Forearm Flap. *The journal of sexual medicine*, *17*(5), 1012-1024. https://doi.org/10.1016/j.jsxm.2020.02.014  Kuper, L. E., Nussbaum, R., & Mustanski, B. (2012). Exploring the diversity of gender and sexual orientation identities in an online sample of transgender individuals. *Journal of Sex Research*, *49*(2-3), 244-254. https://doi.org/10.1080/00224499.2011.596954  Lafortune, D., Dussault, E., Philibert, M., & Godbout, N. (2022). Prevalence and Correlates of Sexual Aversion: A Canadian Community-Based Study. *Journal of Sexual Medicine*, *19*(8), 1269-1280. https://doi.org/10.1016/j.jsxm.2022.05.142  Lampis, J., De Simone, S., Lasio, D., & Serri, F. (2023). The Role of Family Support and Dyadic Adjustment on the Psychological Well-being of Transgender Individuals: An Exploratory Study. *Sexuality Research and Social Policy*, *20*(4), 1328-1344. https://doi.org/10.1007/s13178-023-00817-z  Lane, M., Kirsch, M. J., Sluiter, E. C., Svientek, S. R., Hamill, J. B., Morrison, S. D., Ives, G. C., Alman, E., Gilman, R. H., Kuzon, W. M., Cederna, P. S., & Wilkins, E. G. (2021). Gender Affirming Mastectomy Improves Quality of Life in Transmasculine Patients: A Single-Center Prospective Study. *Annals of surgery*. https://doi.org/10.1097/SLA.0000000000005158  Laube, J. S., Auer, M. K., Biedermann, S. V., Schröder, J., Hildebrandt, T., Nieder, T. O., Briken, P., & Fuss, J. (2020). Sexual behavior, desire, and psychosexual experience in gynephilic and androphilic trans women: A cross-sectional multicenter study. *Journal of Sexual Medicine*, *17*(6), 1182-1194. https://doi.org/10.1016/j.jsxm.2020.01.030  LeBreton, M., Courtois, F., Journel, N. M., Beaulieu-Prévost, D., Bélanger, M., Ruffion, A., & Terrier, J.-É. (2017). Genital sensory detection thresholds and patient satisfaction with vaginoplasty in male-to-female transgender women. *Journal of Sexual Medicine*, *14*(2), 274-281. https://doi.org/10.1016/j.jsxm.2016.12.005  Li, D. H., Remble, T. A., Macapagal, K., & Mustanski, B. (2019). Stigma on the streets, dissatisfaction in the sheets: Is minority stress associated with decreased sexual functioning among young men who have sex with men? *Journal of Sexual Medicine*, *16*(2), 267-277. https://doi.org/10.1016/j.jsxm.2018.12.010  Lindley, L., Anzani, A., & Galupo, M. P. (2022). Gender dysphoria and sexual well-being among trans masculine and nonbinary individuals. *Archives of Sexual Behavior*. https://doi.org/10.1007/s10508-021-02242-y  Lindroth, M., Zeluf, G., Mannheimer, L. N., & Deogan, C. (2017). Sexual health among transgender people in Sweden. *International Journal of Transgenderism*, *18*(3), 318-327. https://doi.org/10.1080/15532739.2017.1301278  Loch Batista, R., Inácio, M., Prado Arnhold, I. J., Gomes, N. L., Diniz Faria, J. A., Rodrigues de Moraes, D., Frade Costa, E. M., Domenice, S., & Bilharinho Mendonça, B. (2019). Psychosexual Aspects, Effects of Prenatal Androgen Exposure, and Gender Change in 46,XY Disorders of Sex Development. *The Journal of clinical endocrinology and metabolism*, *104*(4), 1160-1170. https://doi.org/10.1210/jc.2018-01866  Logie, C. H., Sokolovic, N., Kazemi, M., Smith, S., Islam, S., Lee, M., Gormley, R., Kaida, A., de Pokomandy, A., & Loutfy, M. (2022). Recent sex work and associations with psychosocial outcomes among women living with HIV: findings from a longitudinal Canadian cohort study. *Journal of the International AIDS Society*, *25*(3), e25874. https://doi.org/10.1002/jia2.25874  Loza, O., Mangadu, T., Ferreira-Pinto, J. B., & Guevara, P. (2021). Differences in Substance Use and Sexual Risk by Sexual Orientation and Gender Identity Among University and Community Young Adults in a U.S.-Mexico Border City. *Health promotion practice*, *22*(4), 559-573. https://doi.org/10.1177/1524839920933257  MacGilleEathain, R., Smith, T., & Steele, I. (2024). Sexual well-being among young people in remote rural island communities in Scotland: a mixed methods study. *BMJ sexual & reproductive health*, *50*(1), 7-12. https://doi.org/10.1136/bmjsrh-2023-201822  MacGregor, L., Speare, N., Nicholls, J., Harryman, L., Horwood, J., Kesten, J. M., Lorenc, A., Horner, P., Edelman, N. L., Muir, P., North, P., Gompels, M., & Turner, K. M. E. (2021). Evidence of changing sexual behaviours and clinical attendance patterns, alongside increasing diagnoses of STIs in MSM and TPSM. *Sexually transmitted infections*, *97*(7), 507-513. https://doi.org/10.1136/sextrans-2020-054588  Manrique, O. J., Sabbagh, M. D., Ciudad, P., Martinez-Jorge, J., Kiranantawat, K., Sitpahul, N., Nippoldt, T. B., Charafeddine, A., & Chen, H.-C. (2018). Gender-Confirmation Surgery Using the Pedicle Transverse Colon Flap for Vaginal Reconstruction: A Clinical Outcome and Sexual Function Evaluation Study. *Plastic and reconstructive surgery*, *141*(3), 767-771. https://doi.org/10.1097/PRS.0000000000004122  Mark, K. P., Toland, M. D., Rosenkrantz, D. E., Brown, H. M., & Hong, S.-h. (2018). Validation of the Sexual Desire Inventory for lesbian, gay, bisexual, trans, and queer adults. *Psychology of Sexual Orientation and Gender Diversity*, *5*(1), 122-128. https://doi.org/10.1037/sgd0000260  Mattelin, E., Strandell, A., & Bryman, I. (2022). Fertility preservation and fertility treatment in transgender adolescents and adults in a Swedish region, 2013-2018. *Human reproduction open*, *2022*(2), hoac008. https://doi.org/10.1093/hropen/hoac008  McClelland, S. I. (2014). 'What do you mean when you say that you are sexually satisfied?' A mixed methods study. *Feminism & Psychology*, *24*(1), 74-96. https://doi.org/10.1177/0959353513508392  McFarland, W., Wilson, E. C., & Raymond, H. F. (2017). HIV prevalence, sexual partners, sexual behavior and HIV acquisition risk among trans men, San Francisco, 2014. *AIDS and Behavior*, *21*(12), 3346-3352. https://doi.org/10.1007/s10461-017-1735-4  Meier, S. C., Pardo, S. T., Labuski, C., & Babcock, J. (2013). Measures of clinical health among female-to-male transgender persons as a function of sexual orientation. *Archives of Sexual Behavior*, *42*(3), 463-474. https://doi.org/10.1007/s10508-012-0052-2  Mijuskovic, B., Niggli, S., Bausch, K., Nunez, D. G., Schaefer, D. J., & Feicke, A. (2024). Feminizing genital gender affirmation surgery: Patient-reported outcomes of urethral flap and classical penile inversion techniques. *International Journal of Transgender Health*. https://doi.org/10.1080/26895269.2024.2305201  Minten, M. J., & Dykeman, C. (2021). The impact of a marriage checkup with transgender couples. *Sexologies: European Journal of Sexology and Sexual Health / Revue européenne de sexologie et de santé sexuelle*, *30*(2), e93-e99. https://doi.org/10.1016/j.sexol.2020.09.005  Mittleman, J. (2023). Sexual Fluidity: Implications for Population Research. *Demography*, *60*(4), 1257-1282. https://doi.org/10.1215/00703370-10898916  Mohr, S., Gygax, L. N., Imboden, S., Mueller, M. D., & Kuhn, A. (2021). Screening for HPV and dysplasia in transgender patients: Do we need it? *European journal of obstetrics, gynecology, and reproductive biology*, *260*, 177-182. https://doi.org/10.1016/j.ejogrb.2021.03.030  Monterde-i-Bort, H., Herrera, I., & Guardiola, C. (2023). Myths, Fallacies and "Machismo" Prejudices in the Practice of Sex: A Study on Attitudes and Knowledge for Sexual Life in a Large Spanish Sample. *Sexuality Research and Social Policy*. https://doi.org/10.1007/s13178-023-00878-0  Morgan, E., Dyar, C., Newcomb, M. E., D'Aquila, R. T., & Mustanski, B. (2020). PrEP use and sexually transmitted infections are not associated longitudinally in a cohort study of young men who have sex with men and transgender women in Chicago. *AIDS and Behavior*, *24*(5), 1334-1341. https://doi.org/10.1007/s10461-019-02664-9  Morgan, E., Moran, K., Ryan, D. T., Mustanski, B., & Newcomb, M. E. (2018). Threefold increase in PrEP uptake over time with high adherence among young men who have sex with men in Chicago. *AIDS and Behavior*, *22*(11), 3637-3644. https://doi.org/10.1007/s10461-018-2122-5  Moser, A., Ballard, S. M., Jensen, J., & Averett, P. (2023). The influence of cannabis on sexual functioning and satisfaction. *JOURNAL OF CANNABIS RESEARCH*, *5*(1), Article 2. https://doi.org/10.1186/s42238-022-00169-2  Mueller, S. C., Wierckx, K., & T'Sjoen, G. (2020). Neural and hormonal correlates of sexual arousal in transgender persons. *Journal of Sexual Medicine*, *17*(12), 2495-2507. https://doi.org/10.1016/j.jsxm.2020.08.021  Mustanski, B., Ryan, D. T., Remble, T. A., D'Aquila, R. T., Newcomb, M. E., & Morgan, E. (2018). Discordance of self-report and laboratory measures of HIV viral load among young men who have sex with men and transgender women in Chicago: Implications for epidemiology, care, and prevention. *AIDS and Behavior*, *22*(7), 2360-2367. https://doi.org/10.1007/s10461-018-2112-7  Narula, V., Ahuja, K., & Srivastava, P. (2019). Relationship satisfaction and body image: A comparative study between transgender and non-transgender population. *Journal of Psychosocial Research*, *14*(2), 285-294. https://doi.org/10.32381/JPR.2019.14.02.5  Nematollahi, A., Gharibzadeh, S., Damghanian, M., Gholamzadeh, S., & Farnam, F. (2022). Sexual behaviors and vulnerability to sexually transmitted infections among transgender women in Iran. *BMC women's health*, *22*(1), 170. https://doi.org/10.1186/s12905-022-01753-7  Nikkelen, S. W. C., & Kreukels, B. P. C. (2018). Sexual experiences in transgender people: The role of desire for gender-confirming interventions, psychological well-being, and body satisfaction. *Journal of Sex & Marital Therapy*, *44*(4), 370-381. https://doi.org/10.1080/0092623X.2017.1405303  Nuttbrock, L., Bockting, W., Rosenblum, A., Hwahng, S., Mason, M., Macri, M., & Becker, J. (2013). Gender abuse, depressive symptoms, and HIV and other sexually transmitted infections among male-to-female transgender persons: A three-year prospective study. *American Journal of Public Health*, *103*(2), 300-307. https://doi.org/10.2105/AJPH.2011.300568  Oh, S.-K., Kim, G.-W., Yang, J.-C., Kim, S.-K., Kang, H.-K., & Jeong, G.-W. (2012). Brain activation in response to visually evoked sexual arousal in male-to-female transsexuals: 3.0 tesla functional magnetic resonance imaging. *Korean journal of radiology*, *13*(3), 257-264. https://doi.org/10.3348/kjr.2012.13.3.257  Olakunde, B. O., Pharr, J. R., Adeyinka, D. A., & Conserve, D. F. (2022). Nonuptake of HIV Testing Among Transgender Populations in the United States: Results from the 2015 US Transgender Survey. *Transgender Health*. https://doi.org/10.1089/trgh.2020.0141  Olthuis, J. V., Connell, E. M., O'Sullivan, L. F., & Byers, E. S. (2023). Does anxiety sensitivity interfere with sexual well-being? Evidence from a community sample. *Sexual and Relationship Therapy*. https://doi.org/10.1080/14681994.2023.2260990  Olver, M. E., Kingston, D. A., Laverty, E. K., & Seto, M. C. (2022). Psychometric properties of common measures of hypersexuality in an online Canadian sample. *Journal of Sexual Medicine*, *19*(2), 331-346. https://doi.org/10.1016/j.jsxm.2021.12.002  Onwubiko, U., Holland, D., Ajoku, S., Taylor, J., Childs, A., Wilson, D., & Chamberlain, A. T. (2020). Using PrEP to #STOPHIVATL: Findings from a cross-sectional survey among gay men and transgender women participating in gay pride events in Atlanta, Georgia, 2018. *Archives of Sexual Behavior*, *49*(6), 2193-2204. https://doi.org/10.1007/s10508-020-01711-0  Papadopulos, N. A., Lellé, J.-D., Zavlin, D., Herschbach, P., Henrich, G., Kovacs, L., Ehrenberger, B., Machens, H.-G., & Schaff, J. (2020). Psychological Pathologies and Sexual Orientation in Transgender Women Undergoing Gender Confirming Treatment. *Annals of plastic surgery*, *84*(3), 312-316. https://doi.org/10.1097/SAP.0000000000002035  Parmenter, J. G., Galliher, R. V., Berke, R., & Barrett, T. S. (2023). Configurations of sexual and vocational identity processes among sexual minority college students. *Journal of Lgbtq Issues in Counseling*, *17*(4), 306-323. https://doi.org/10.1080/26924951.2023.2200987  Parsons, J. T., Antebi-Gruszka, N., Millar, B. M., Cain, D., & Gurung, S. (2018). Syndemic conditions, HIV transmission risk behavior, and transactional sex among transgender women. *AIDS and Behavior*, *22*(7), 2056-2067. https://doi.org/10.1007/s10461-018-2100-y  Passaro, R. C., Segura, E. R., Gonzales-Saavedra, W., Lake, J. E., Perez-Brumer, A., Shoptaw, S., Dilley, J., Cabello, R., & Clark, J. L. (2020). Sexual partnership-level correlates of intimate partner violence among men who have sex with men and transgender women in Lima, Peru. *Archives of Sexual Behavior*, *49*(7), 2703-2713. https://doi.org/10.1007/s10508-020-01682-2  Peitzmeier, S. M., Khullar, K., Reisner, S. L., & Potter, J. (2014). Pap test use is lower among female-to-male patients than non-transgender women. *American Journal of Preventive Medicine*, *47*(6), 808-812. https://doi.org/10.1016/j.amepre.2014.07.031  Peitzmeier, S. M., Todd, K. P., Correll-King, W., Church, D., Thornburgh, S., Adams, M. P., Koss, M. P., & Senn, C. Y. (2024). Toward a More Gender-Inclusive Sexual Experiences Survey: Development and Preliminary Validation With Transgender and Gender-Expansive Survivors of Campus Sexual Assault. *PSYCHOLOGY OF WOMEN QUARTERLY*. https://doi.org/10.1177/03616843241261666  Pella, R. S., & McClung, N. A. (2024). Does Yes mean yes? Differences in US college students’ understanding of sexual consent. *Sex Education*, *24*(3), 416-432. https://doi.org/10.1080/14681811.2023.2211514  Phillips, T. R., Fairley, C. K., Maddaford, K., McNulty, A., Donovan, B., Guy, R., McIver, R., Wigan, R., Varma, R., Ong, J. J., Callander, D., Skelsey, G., Pony, M., O'Hara, D., Bilardi, J. E., & Chow, E. P. (2024). Understanding Risk Factors for Oropharyngeal Gonorrhea Among Sex Workers Attending Sexual Health Clinics in 2 Australian Cities: Mixed Methods Study. *JMIR public health and surveillance*, *10*, e46845. https://doi.org/10.2196/46845  Pigot, G. L. S., Al-Tamimi, M., Nieuwenhuijzen, J. A., van der Sluis, W. B., Moorselaar, R. J. A. v., Mullender, M. G., van de Grift, T. C., & Bouman, M.-B. (2020). Genital Gender-Affirming Surgery Without Urethral Lengthening in Transgender Men-A Clinical Follow-Up Study on the Surgical and Urological Outcomes and Patient Satisfaction. *The journal of sexual medicine*, *17*(12), 2478-2487. https://doi.org/10.1016/j.jsxm.2020.08.004  Pines, H. A., Patrick, R., Smith, D. M., Harvey-Vera, A., Blumenthal, J. S., Rangel, G., Semple, S. J., & Patterson, T. L. (2020). HIV prevention method preferences within sexual partnerships reported by HIV-negative MSM and TW in Tijuana, Mexico. *AIDS and Behavior*, *24*(3), 839-846. https://doi.org/10.1007/s10461-019-02492-x  Pletta, D. R., White Hughto, J. M., Peitzmeier, S., Deutsch, M. B., Pardee, D., Potter, J., & Reisner, S. L. (2020). Individual- and partnership-level correlates of protective barrier use in a sample of transmasculine adults with diverse sexual partnerships. *AIDS Patient Care and STDs*, *34*(5), 237-246. https://doi.org/10.1089/apc.2019.0296  Pollitt, A. M., & Martin-Storey, A. (2024). Relationship satisfaction among plurisexual young adults: Understanding the unique role of identity abuse. *Psychology of Sexual Orientation and Gender Diversity*. https://doi.org/10.1037/sgd0000746  Poteat, T., Ackerman, B., Diouf, D., Ceesay, N., Mothopeng, T., Odette, K.-Z., Kouanda, S., Ouedraogo, H. G., Simplice, A., Kouame, A., Mnisi, Z., Trapence, G., van der Merwe, L. L. A., Jumbe, V., & Baral, S. (2017). HIV prevalence and behavioral and psychosocial factors among transgender women and cisgender men who have sex with men in 8 African countries: A cross-sectional analysis. *PLoS medicine*, *14*(11), e1002422. https://doi.org/10.1371/journal.pmed.1002422  Poteat, T. C., Celentano, D. D., Mayer, K. H., Beyrer, C., Mimiaga, M. J., Friedman, R. K., Srithanaviboonchai, K., & Safren, S. A. (2020). Depression, sexual behavior, and HIV treatment outcomes among transgender women, cisgender women and men who have sex with men living with HIV in Brazil and Thailand: A short report. *AIDS Care*, *32*(3), 310-315. https://doi.org/10.1080/09540121.2019.1668526  Potter, E., Sivagurunathan, M., Armstrong, K., Barker, L. C., Du Mont, J., Lorello, G. R., Millman, A., Urbach, D. R., & Krakowsky, Y. (2023). Patient reported symptoms and adverse outcomes seen in Canada's first vaginoplasty postoperative care clinic. *NEUROUROLOGY AND URODYNAMICS*, *42*(2), 523-529. https://doi.org/10.1002/nau.25132  Rahman, M., Li, D. H., & Moskowitz, D. A. (2019). Comparing the healthcare utilization and engagement in a sample of transgender and cisgender bisexual+ persons. *Archives of Sexual Behavior*, *48*(1), 255-260. https://doi.org/10.1007/s10508-018-1164-0  Raines, J., Holmes, L., Watts-Overall, T. M., Slettevold, E., Gruia, D. C., Orbell, S., & Rieger, G. (2021). Patterns of genital sexual arousal in transgender men. *Psychological Science*, *32*(4), 485-495. https://doi.org/10.1177/0956797620971654  Ranjit, Y. S., Krishnan, A., Earnshaw, V. A., Weikum, D., Ferro, E. G., Sanchez, J., & Altice, F. L. (2021). Psychometric evaluation and validation of the HIV Stigma Scale in Spanish among men who have sex with men and transgender women. *Stigma and Health*. https://doi.org/10.1037/sah0000302  Reisner, S. L., Moore, C. S., Asquith, A., Pardee, D. J., & Mayer, K. H. (2020). Gender non-affirmation from cisgender male partners: Development and validation of a brief stigma scale for HIV research with transgender men who have sex with men (trans MSM). *AIDS and Behavior*, *24*(1), 331-343. https://doi.org/10.1007/s10461-019-02749-5  Reisner, S. L., Vetters, R., White, J. M., Cohen, E. L., LeClerc, M., Zaslow, S., Wolfrum, S., & Mimiaga, M. J. (2015). Laboratory-confirmed HIV and sexually transmitted infection seropositivity and risk behavior among sexually active transgender patients at an adolescent and young adult urban community health center. *AIDS Care*, *27*(8), 1031-1036. https://doi.org/10.1080/09540121.2015.1020750  Reisner, S. L., White, J. M., Mayer, K. H., & Mimiaga, M. J. (2014). Sexual risk behaviors and psychosocial health concerns of female-to-male transgender men screening for STD'S at an urban community health center. *AIDS Care*, *26*(7), 857-864. https://doi.org/10.1080/09540121.2013.855701  Ribeiro dos Santos, P. M., Cardoso dos Santos, K., Rodrigues de Oliveira, B., Silva Magalhães, L., Amorim Caetano, K. A., da Silva Carvalho Vila, V., Rezende Pacheco, L., dos Santos Carneiro, M. A., Ferreira Dias, S., Sttaciarini, J.-M., & Araujo Teles, S. (2022). Risk perception and vulnerabilities for hiv/aids among transgender women in brazil: Mixed methods approach. *AIDS Care*. https://doi.org/10.1080/09540121.2022.2067316  Riggs, D. W., von Doussa, H., & Power, J. (2015). The family and romantic relationships of trans and gender diverse Australians: An exploratory survey. *Sexual and Relationship Therapy*, *30*(2), 243-255. https://doi.org/10.1080/14681994.2014.992409  Riquelme, J. B., Naser, N. F., Puertas, J. B., Kalil, J. A., & Arevalo, M. C. (2021). Gender affirmation surgeries in transgender women: Aesthetic, sexual, and urinary results of an initial series of vaginoplasties. *Actas Urologicas Espanolas*, *45*(3), 225-231. https://doi.org/10.1016/j.acuro.2020.08.007  Ristori, J., Cocchetti, C., Castellini, G., Pierdominici, M., Cipriani, A., Testi, D., Gavazzi, G., Mazzoli, F., Mosconi, M., Meriggiola, M. C., Cassioli, E., Vignozzi, L., Ricca, V., Maggi, M., & Fisher, A. D. (2020). Hormonal treatment effect on sexual distress in transgender persons: 2-year follow-up data. *Journal of Sexual Medicine*, *17*(1), 142-151. https://doi.org/10.1016/j.jsxm.2019.10.008  Rose, S. B., Garrett, S. M., McKinlay, E. M., & Morgan, S. J. (2021). Access to sexual healthcare during New Zealand's COVID-19 lockdown: cross-sectional online survey of 15-24-year-olds in a high deprivation region. *BMJ sexual & reproductive health*, *47*(4), 277-284. https://doi.org/10.1136/bmjsrh-2020-200986  Rubinsky, V. (2022). Sex Talk: The Effects of Sexual Self-Disclosure and Identity Gaps on Sexual and Relational Outcomes in Diverse Relationships. *SEXUALITY & CULTURE-AN INTERDISCIPLINARY JOURNAL*, *26*(4), 1452-1476. https://doi.org/10.1007/s12119-022-09953-x  Sahin, S., Korkmaz, O. P., Durcan, E., Ozkaya, H. M., Turan, S., & Kadioglu, P. (2021). Sexual Functions of Transgender Individuals Before Gender Transition. *Turkish Journal of Endocrinology and Metabolism*, *25*(2), 184-192. https://doi.org/10.25179/tjem.2021-81979  Salvatori, G., Amoroso, M., Fantacci, F., Giunti, D., Olmi, A., Borrello, L., & Antonelli, P. (2024). To do or not to do: a study about sexual fantasies in an Italian sample. *Sexual and Relationship Therapy*. https://doi.org/10.1080/14681994.2024.2308055  Sarno, E. L., Dyar, C., Newcomb, M. E., & Whitton, S. W. (2022). Relationship quality and mental health among sexual and gender minorities. *Journal of Family Psychology*, *36*(5), 770-779. https://doi.org/10.1037/fam0000944  Sarno, E. L., Swann, G., Newcomb, M. E., & Whitton, S. W. (2024). Relationship risk factors for intimate partner violence among sexual and gender minorities: A multilevel analysis. *FAMILY PROCESS*, *63*(2), 983-1000. https://doi.org/10.1111/famp.12941  Sarno, E. L., Swann, G., Xavier Hall, C. D., Newcomb, M. E., & Mustanski, B. (2022). Minority stress, identity conflict, and hiv-related outcomes among men who have sex with men, transgender women, and gender nonbinary people of color. *LGBT Health*. https://doi.org/10.1089/lgbt.2021.0401  Satcher, M. F., Segura, E. R., Silva-Santisteban, A., Reisner, S. L., Perez-Brumer, A., Lama, J. R., Operario, D., & Clark, J. L. (2022). Exploring contextual differences for sexual role strain among transgender women and men who have sex with men in lima, peru. *Archives of Sexual Behavior*. https://doi.org/10.1007/s10508-021-02181-8  Scheim, A. I., & Bauer, G. R. (2019). Sexual inactivity among transfeminine persons: A Canadian respondent-driven sampling survey. *Journal of Sex Research*, *56*(2), 264-271. https://doi.org/10.1080/00224499.2017.1399334  Scheim, A. I., Bauer, G. R., & Travers, R. (2017). HIV-related sexual risk among transgender men who are gay, bisexual, or have sex with men. *JAIDS Journal of Acquired Immune Deficiency Syndromes*, *74*(4), e89-e96. https://doi.org/10.1097/QAI.0000000000001222  Scott, S. B., Pulice-Farrow, L., Do, Q. A., Brunett, K. M., & Balsam, K. F. (2023). Intimate Partner Violence in Transgender and Nonbinary Relationships: Actor-Partner Associations with Relationship Satisfaction, Dyadic Coping, and Partner Support. *LGBT Health*, *10*, S20-S27. https://doi.org/10.1089/lgbt.2023.0112  Seyed-Forootan, K., Karimi, H., & Seyed-Forootan, N.-S. (2018). Autologous Fibroblast-Seeded Amnion for Reconstruction of Neo-vagina in Male-to-Female Reassignment Surgery. *Aesthetic plastic surgery*, *42*(2), 491-497. https://doi.org/10.1007/s00266-018-1088-z  Shan, D., Yu, M.-H., Yang, J., Zhuang, M.-H., Ning, Z., Liu, H., Liu, L., Han, M.-J., & Zhang, D.-P. (2018). Correlates of HIV infection among transgender women in two Chinese cities. *Infectious diseases of poverty*, *7*(1), 123. https://doi.org/10.1186/s40249-018-0508-2  Sharman, L. S., Fitzgerald, R., & Douglas, H. (2024). Prevalence of Sexual Strangulation/Choking Among Australian 18-35 Year-Olds. *Archives of Sexual Behavior*. https://doi.org/10.1007/s10508-024-02937-y  Shaw, S. Y., Lorway, R., Bhattacharjee, P., Reza-Paul, S., du Plessis, E., McKinnon, L., Thompson, L. H., Isac, S., Ramesh, B. M., Washington, R., Moses, S., & Blanchard, J. F. (2016). Descriptive epidemiology of factors associated with HIV infections among men and transgender women who have sex with men in South India. *LGBT Health*, *3*(4), 292-299. https://doi.org/10.1089/lgbt.2015.0023  Shover, C. L., DeVost, M. A., Beymer, M. R., Gorbach, P. M., Flynn, R. P., & Bolan, R. K. (2018). Using sexual orientation and gender identity to monitor disparities in HIV, sexually transmitted infections, and viral hepatitis. *American Journal of Public Health*, *108*(Suppl 4), S277-S283. https://doi.org/10.2105/AJPH.2018.304751  Shrader, C.-H., Duncan, D. T., Chen, Y.-T., Driver, R., Russell, J., Moody, R. L., Knox, J., Skaathun, B., Durrell, M., Hanson, H., Eavou, R., Goedel, W. C., & Schneider, J. A. (2023). Latent profile patterns of network-level norms and associations with individual-level sexual behaviors: The N2 cohort study in Chicago. *Archives of Sexual Behavior*, *52*(6), 2355-2372. https://doi.org/10.1007/s10508-023-02555-0  Sigurjónsson, H., Möllermark, C., Rinder, J., Farnebo, F., & Lundgren, T. K. (2017). Long-Term Sensitivity and Patient-Reported Functionality of the Neoclitoris After Gender Reassignment Surgery. *The journal of sexual medicine*, *14*(2), 269-273. https://doi.org/10.1016/j.jsxm.2016.12.003  Silva, J. F., Mota, M., Fernandes, E. P., & Esteves, M. F. (2022). Recognizing the diversity of the Portuguese transgender population: A cross-sectional study. *SEXOLOGIES*, *31*(4), 280-286, Article 380-386. https://doi.org/10.1016/j.sexol.2022.09.002  Silva-Santisteban, A., Raymond, H. F., Salazar, X., Villayzan, J., Leon, S., McFarland, W., & Caceres, C. F. (2012). Understanding the HIV/AIDS epidemic in transgender women of Lima, Peru: Results from a sero-epidemiologic study using respondent driven sampling. *AIDS and Behavior*, *16*(4), 872-881. https://doi.org/10.1007/s10461-011-0053-5  Simonsen, R., Hald, G. M., Giraldi, A., & Kristensen, E. (2015). Sociodemographic Study of Danish Individuals Diagnosed with Transsexualism. *Sexual medicine*, *3*(2), 109-117. https://doi.org/10.1002/sm2.48  Sizemore, K. M., Carter, J. A., Millar, B. M., Cain, D., Parsons, J. T., & Rendina, H. J. (2019). Attachment as a predictor of psychological and sexual wellbeing among transgender women in New York City. *Journal of Sex Research*, *56*(9), 1192-1202. https://doi.org/10.1080/00224499.2019.1644486  Spizzirri, G., Eufrásio, R. Á., Abdo, C. H. N., & Lima, M. C. P. (2022). Proportion of ALGBT adult Brazilians, sociodemographic characteristics, and self-reported violence. *Scientific reports*, *12*(1), 11176. https://doi.org/10.1038/s41598-022-15103-y  Stanton, M. C., Ali, S., & Chaudhuri, S. (2017). Individual, social and community-level predictors of wellbeing in a US sample of transgender and gender non-conforming individuals. *Culture, Health & Sexuality*, *19*(1), 32-49. https://doi.org/10.1080/13691058.2016.1189596  Staples, J. M., Bird, E. R., Gregg, J. J., & George, W. (2020). Improving the gender-affirmation process for transgender and gender-nonconforming individuals: Associations among time since transition began, body satisfaction, and sexual distress. *Journal of Sex Research*, *57*(3), 375-383. https://doi.org/10.1080/00224499.2019.1617829  Stelmar, J., Smith, S. M., Lee, G., Zaliznyak, M., & Garcia, M. M. (2023). Shallow-depth vaginoplasty: preoperative goals, postoperative satisfaction, and why shallow-depth vaginoplasty should be offered as a standard feminizing genital gender-affirming surgery option. *The journal of sexual medicine*, *20*(11), 1333-1343. https://doi.org/10.1093/jsxmed/qdad111  Sun, C. J., Sutfin, E., Bachmann, L. H., Stowers, J., & Rhodes, S. D. (2018). Comparing men who have sex with men and transgender women who use Grindr, other similar social and sexual networking apps, or no social and sexual networking apps: Implications for recruitment and health promotion. *Journal of AIDS & clinical research*, *9*(2). https://doi.org/10.4172/2155-6113.1000757  Taşkın, L., Şentürk Erenel, A., Yaman Sözbir, Ş., Gönenç, İ. M., Yücel, Ç., Alan Dikmen, H., & Çetinkaya, Ş. Ş. (2020). Sexual Health/Reproductive Health-Related Problems of Lesbian, Gay, Bisexual and Transgender People in Turkey and Their Health-Care Needs. *Florence Nightingale journal of nursing*, *28*(1), 97-109. https://doi.org/10.5152/FNJN.2020.19032  Thammapiwan, P., Suwan, A., Panyakhamlerd, K., Suwajo, P., Phanuphak, N., & Taechakraichana, N. (2022). The sexual function among transgender women who have undergone gender-affirming surgery using penile skin inversion vaginoplasty in Thailand. *European Journal of Plastic Surgery*, *45*(1), 101-107. https://doi.org/10.1007/s00238-021-01857-7  Toussaint, D. J., Schweitzer, R., & Mitchell, R. (2024). Discrimination, Internalized Sexual Prejudice and the Post-Sex Experience Among Members of Sexual Minorities. *Journal of Homosexuality*. https://doi.org/10.1080/00918369.2024.2364881  Trujillo, D., Arayasirikul, S., Xie, H., Sicro, S., Meza, J., Bella, M., Daza, E., Torres, F., McFarland, W., & Wilson, E. C. (2022). Disparities in Sexually Transmitted Infection Testing and the Need to Strengthen Comprehensive Sexual Health Services for Trans Women. *Transgender Health*, *7*(3), 230-236. https://doi.org/10.1089/trgh.2020.0133  Uaamnuichai, S., Panyakhamlerd, K., Suwan, A., Suwajo, P., Phanuphak, N., Ariyasriwatana, C., Janamnuaysook, R., Teeratakulpisarn, N., Vasuratna, A., & Taechakraichana, N. (2021). Neovaginal and Anal High-Risk Human Papillomavirus DNA Among Thai Transgender Women in Gender Health Clinics. *Sexually transmitted diseases*, *48*(8), 547-549. https://doi.org/10.1097/OLQ.0000000000001388  van de Grift, T. C., Pigot, G. L. S., Boudhan, S., Elfering, L., Kreukels, B. P. C., Gijs, L. A. C. L., Buncamper, M. E., Özer, M., van der Sluis, W., Meuleman, E. J. H., Bouman, M.-B., & Mullender, M. G. (2017). A longitudinal study of motivations before and psychosexual outcomes after genital gender-confirming surgery in transmen. *Journal of Sexual Medicine*, *14*(12), 1621-1628. https://doi.org/10.1016/j.jsxm.2017.10.064  van de Grift, T. C., Pigot, G. L. S., Kreukels, B. P. C., Bouman, M.-B., & Mullender, M. G. (2019). Transmen’s experienced sexuality and genital gender-affirming surgery: Findings from a clinical follow-up study. *Journal of Sex & Marital Therapy*, *45*(3), 201-205. https://doi.org/10.1080/0092623X.2018.1500405  van der Sluis, W. B., Bouman, M.-B., de Boer, N. K. H., Buncamper, M. E., van Bodegraven, A. A., Neefjes-Borst, E. A., Kreukels, B. P. C., Meijerink, W. J. H. J., & Mullender, M. G. (2016). Long-term follow-up of transgender women after secondary intestinal vaginoplasty. *Journal of Sexual Medicine*, *13*(4), 702-710. https://doi.org/10.1016/j.jsxm.2016.01.008  Vargas, S. K., Konda, K. A., Leon, S. R., Brown, B., Klausner, J. D., Lindan, C., & Caceres, C. F. (2018). The relationship between risk perception and frequency of HIV testing among men who have sex with men and transgender women, Lima, Peru. *AIDS and Behavior*, *22*(Suppl 1), S26-S34. https://doi.org/10.1007/s10461-017-2018-9  Veale, J., Watson, R. J., Adjei, J., & Saewyc, E. (2016). Prevalence of pregnancy involvement among Canadian transgender youth and its relation to mental health, sexual health, and gender identity. *International Journal of Transgenderism*, *17*(3-4), 107-113. https://doi.org/10.1080/15532739.2016.1216345  Vedovo, F., Di Blas, L., Aretusi, F., Falcone, M., Perin, C., Pavan, N., Rizzo, M., Morelli, G., Cocci, A., Polito, C., Gentile, G., Colombo, F., Timpano, M., Verze, P., Imbimbo, C., Bettocchi, C., Pascolo Fabrici, E., Palmieri, A., & Trombetta, C. (2021). Physical, mental and sexual health among transgender women: A comparative study among operated transgender and cisgender women in a National Tertiary Referral Network. *Journal of Sexual Medicine*, *18*(5), 982-989. https://doi.org/10.1016/j.jsxm.2021.02.006  Vedovo, F., Di Blas, L., Perin, C., Pavan, N., Zatta, M., Bucci, S., Morelli, G., Cocci, A., Delle Rose, A., Grisanti, S. C., Gentile, G., Colombo, F., Rolle, L., Timpano, M., Verze, P., Spirito, L., Schiralli, F., Bettocchi, C., Garaffa, G., Palmieri, A., Mirone, V., & Trombetta, C. (2020). OPERATED MALE TO FEMALE SEXUAL FUNCTION INDEX (OMTFSFI): A STUDY ON THE VALIDITY OF THE FIRST QUESTIONNAIRE DEVELOPED IN ORDER TO ASSESS THE SEXUAL FUNCTION AFTER MALE TO FEMALE GENDER REASSIGNMENT SURGERY. *Journal of Sexual Medicine*, *17*(6), S207-S207. <Go to ISI>://WOS:000539164700252  Veronese, V., Oo, Z. M., Thein, Z. W., Aung, P. P., Draper, B. L., Hughes, C., Ryan, C., Pedrana, A., & Stoové, M. (2018). Acceptability of peer-delivered HIV testing and counselling among men who have sex with men (MSM) and transgender women (TW) in Myanmar. *AIDS and Behavior*, *22*(8), 2426-2434. https://doi.org/10.1007/s10461-017-2022-0  Veronese, V., van Gemert, C., Bulu, S., Kwarteng, T., Bergeri, I., Badman, S., Vella, A., & Stoové, M. (2015). Sexually transmitted infections among transgender people and men who have sex with men in Port Vila, Vanuatu. *Western Pacific surveillance and response journal : WPSAR*, *6*(1), 55-59. https://doi.org/10.2471/WPSAR.2014.5.1.001  Vukadinovic, V., Stojanovic, B., Majstorovic, M., & Milosevic, A. (2014). The role of clitoral anatomy in female to male sex reassignment surgery. *TheScientificWorldJournal*, *2014*, 437378. https://doi.org/10.1155/2014/437378  Weigert, R., Frison, E., Sessiecq, Q., Al Mutairi, K., & Casoli, V. (2013). Patient satisfaction with breasts and psychosocial, sexual, and physical well-being after breast augmentation in male-to-female transsexuals. *Plastic and reconstructive surgery*, *132*(6), 1421-1429. https://doi.org/10.1097/01.prs.0000434415.70711.49  Westlake, B., & Mahan, I. (2023). An International Survey of BDSM Practitioner Demographics: The Evolution of Purpose for, Participation in, and Engagement with, Kink Activities. *Journal of Sex Research*. https://doi.org/10.1080/00224499.2023.2273266  Whitton, S. W., Bothwell, S. J., Crosby, S. T., & Newcomb, M. E. (2023). Sexual and Gender Minority Individuals' Perspectives on How the COVID-19 Pandemic Has Affected Their Couple Relationships. *COUPLE AND FAMILY PSYCHOLOGY-RESEARCH AND PRACTICE*. https://doi.org/10.1037/cfp0000246  Wierckx, K., Elaut, E., Van Hoorde, B., Heylens, G., De Cuypere, G., Monstrey, S., Weyers, S., Hoebeke, P., & T'Sjoen, G. (2014). Sexual desire in trans persons: associations with sex reassignment treatment. *The journal of sexual medicine*, *11*(1), 107-118. https://doi.org/10.1111/jsm.12365  Wilbourn, B., Howard-Howell, T., Castel, A., D'Angelo, L., Trexler, C., Carr, R., & Greenberg, D. (2022). Barriers and Facilitators to HIV Testing Among Adolescents and Young Adults in Washington, District of Columbia: Formative Research to Inform the Development of an mHealth Intervention. *JMIR formative research*, *6*(3), e29196. https://doi.org/10.2196/29196  Willie, T. C., Chakrapani, V., White Hughto, J. M., & Kershaw, T. S. (2017). Victimization and human immunodeficiency virus-related risk among transgender women in India: A latent profile analysis. *Violence and Gender*, *4*(4), 121-129. https://doi.org/10.1089/vio.2017.0030  Wilson, E. C., Chen, Y.-H., Arayasirikul, S., Fisher, M., Pomart, W. A., Le, V., Raymond, H. F., & McFarland, W. (2015). Differential HIV risk for racial/ethnic minority trans*female youths and socioeconomic disparities in housing, residential stability, and education. *American Journal of Public Health*, *105 Suppl 3*, e41-e47. https://doi.org/10.2105/AJPH.2014.302443  Yang, X., Zhao, L., Wang, L., Hao, C., Gu, Y., Song, W., Zhao, Q., & Wang, X. (2016). Quality of life of transgender women from china and associated factors: A cross-sectional study. *Journal of Sexual Medicine*, *13*(6), 977-987. https://doi.org/10.1016/j.jsxm.2016.03.369  Ye, Z., Mohammadi, B., Kopyciok, R., Heldmann, M., Samii, A., & Münte, T. F. (2018). Androgens modulate brain responses to sexual stimuli in female-to-male transsexuals: An fMRI study. *Zeitschrift für Neuropsychologie*, *29*(2), 89-98. https://doi.org/10.1024/1016-264X/a000219  Zavlin, D., Schaff, J., Lellé, J.-D., Jubbal, K. T., Herschbach, P., Henrich, G., Ehrenberger, B., Kovacs, L., Machens, H.-G., & Papadopulos, N. A. (2018). Male-to-Female Sex Reassignment Surgery using the Combined Vaginoplasty Technique: Satisfaction of Transgender Patients with Aesthetic, Functional, and Sexual Outcomes. *Aesthetic plastic surgery*, *42*(1), 178-187. https://doi.org/10.1007/s00266-017-1003-z  Zavlin, D., Wassersug, R. J., Chegireddy, V., Schaff, J., & Papadopulos, N. A. (2019). Age-Related Differences for Male-to-Female Transgender Patients Undergoing Gender-Affirming Surgery. *Sexual medicine*, *7*(1), 86-93. https://doi.org/10.1016/j.esxm.2018.11.005  Zeigler-Hill, V., & LaCross, D. (2023). The desire for power and romantic commitment in LGBTQ relationships. *Interpersona: An International Journal on Personal Relationships*, *17*(2), 197-212. https://doi.org/10.5964/ijpr.7989  Zhang, Y., Best, J., Tang, W., Tso, L. S., Liu, F., Huang, S., Zheng, H., Yang, B., Wei, C., & Tucker, J. D. (2016). Transgender sexual health in China: a cross-sectional online survey in China. *Sexually transmitted infections*, *92*(7), 515-519. https://doi.org/10.1136/sextrans-2015-052350  Zucker, K. J., Bradley, S. J., Owen-Anderson, A., Kibblewhite, S. J., Wood, H., Singh, D., & Choi, K. (2012). Demographics, behavior problems, and psychosexual characteristics of adolescents with gender identity disorder or transvestic fetishism. *Journal of Sex & Marital Therapy*, *38*(2), 151-189. https://doi.org/10.1080/0092623X.2011.611219  Zwickl, S., Burchill, L., Wong, A. F. Q., Leemaqz, S. Y., Cook, T., Angus, L. M., Eshin, K., Elder, C. V., Grover, S. R., Zajac, J. D., & Cheung, A. S. (2023). Pelvic Pain in Transgender People Using Testosterone Therapy. *LGBT Health*, *10*(3), 179-190. https://doi.org/10.1089/lgbt.2022.0187 |
| **No focus on sexual variables of interest** | Andrzejewski, J., Dunville, R., Johns, M. M., Michaels, S., & Reisner, S. L. (2021). Medical gender affirmation and HIV and sexually transmitted disease prevention in transgender youth: Results from the survey of today's adolescent relationships and transitions, 2018. *LGBT Health*, *8*(3), 181-189. https://doi.org/10.1089/lgbt.2020.0367  Ang, D., Liu, Y., & Eisingerich, A. B. (2019). Difference in new product adoption among at-risk members of society: A critical analysis of males, females, and transgender individuals. *Personality and Individual Differences*, *151*. https://doi.org/10.1016/j.paid.2019.07.013  Antos, N., Flores, R., Harawa, N., Vecchio, N. D., Issema, R., Fujimoto, K., Khanna, A. S., Paola, A. D., Schneider, J. A., & Hotton, A. L. (2022). Factors associated with hiv testing and treatment among young black msm and trans women in three jail systems. *AIDS Care*. https://doi.org/10.1080/09540121.2022.2094312  Arseneau, J. R., Grzanka, P. R., Miles, J. R., & Fassinger, R. E. (2013). Development and initial validation of the Sexual Orientation Beliefs Scale (SOBS). *Journal of Counseling Psychology*, *60*(3), 407-420. https://doi.org/10.1037/a0032799  Berner, A. M., Connolly, D. J., Pinnell, I., Wolton, A., MacNaughton, A., Challen, C., Nambiar, K., Bayliss, J., Barrett, J., & Richards, C. (2021). Attitudes of transgender men and non-binary people to cervical screening: a cross-sectional mixed-methods study in the UK. *The British journal of general practice : the journal of the Royal College of General Practitioners*, *71*(709), e614-e625. https://doi.org/10.3399/BJGP.2020.0905  Callander, D., Cook, T., Read, P., Hellard, M. E., Fairley, C. K., Kaldor, J. M., Vlahakis, E., Pollack, A., Bourne, C., Russell, D. B., Guy, R. J., & Donovan, B. (2019). Sexually transmissible infections among transgender men and women attending Australian sexual health clinics. *The Medical journal of Australia*, *211*(9), 406-411. https://doi.org/10.5694/mja2.50322  Carballo-Diéguez, A., Giguere, R., Balán, I. C., Brown, W., III, Dolezal, C., Leu, C.-S., Lopez Rios, J., Sheinfil, A. Z., Frasca, T., Rael, C. T., Lentz, C., Crespo, R., Iribarren, S., Cruz Torres, C., & Febo, I. (2020). Use of rapid HIV self-test to screen potential sexual partners: Results of the ISUM study. *AIDS and Behavior*, *24*(6), 1929-1941. https://doi.org/10.1007/s10461-019-02763-7  Coffin, P. O., Santos, G.-M., Hern, J., Vittinghoff, E., Walker, J. E., Matheson, T., Santos, D., Colfax, G., & Batki, S. L. (2020). Effects of mirtazapine for methamphetamine use disorder among cisgender men and transgender women who have sex with men: A placebo-controlled randomized clinical trial. *JAMA Psychiatry*, *77*(3), 246-255. https://doi.org/10.1001/jamapsychiatry.2019.3655  Cox, D. W., Fleckenstein, J. R., & Sims-Cox, L. R. (2021). Comparing the self-reported health, happiness, and marital happiness of a multinational sample of consensually non-monogamous adults with those of the uS General population: Additional comparisons by gender, number of sexual partners, frequency of sex, and marital status. *Archives of Sexual Behavior*. https://doi.org/10.1007/s10508-021-01973-2  Defechereux, P. A., Mehrotra, M., Liu, A. Y., McMahan, V. M., Glidden, D. V., Mayer, K. H., Vargas, L., Amico, K. R., Chodacki, P., Fernandez, T., Avelino-Silva, V. I., Burns, D., & Grant, R. M. (2016). Depression and oral FTC/TDF pre-exposure prophylaxis (PrEP) among men and transgender women who have sex with men (MSM/TGW). *AIDS and Behavior*, *20*(7), 1478-1488. https://doi.org/10.1007/s10461-015-1082-2  del pozo de Bolger, A., Jones, T., Dunstan, D., & Lykins, A. (2014). Australian trans men: Development, sexuality, and mental health. *Australian Psychologist*, *49*(6), 395-402. https://doi.org/10.1111/ap.12094  Eisenberg, M. E., Lawrence, S. E., Gower, A. L., Rider, G. N., Brown, C., Crutcher, V., Schuster, A., & Watson, R. J. (2024). Are HIV Prevention Services Reaching all LGBTQ+ Youth? An Intersectional Analysis in a National Sample. *AIDS and Behavior*, *28*(4), 1435-1446. https://doi.org/10.1007/s10461-023-04230-w  Gamarel, K. E., Sevelius, J. M., Reisner, S. L., Coats, C. S., Nemoto, T., & Operario, D. (2019). Commitment, interpersonal stigma, and mental health in romantic relationships between transgender women and cisgender male partners. *Journal of Social and Personal Relationships*, *36*(7), 2180-2201. https://doi.org/10.1177/0265407518785768  Godfrey, L. M., James-Kangal, N., Newcomb, M. E., & Whitton, S. W. (2022). Relationship, marriage, and parenthood aspirations among sexual and gender minority youth assigned female at birth. *Journal of Family Psychology*. https://doi.org/10.1037/fam0000990  Grant, R., Amos, N., Cook, T., Lin, A., Hill, A., Carman, M., & Bourne, A. (2024). From euphoria to wellbeing: Correlates of gender euphoria and its association with mental wellbeing among transgender adults. *International Journal of Transgender Health*. https://doi.org/10.1080/26895269.2024.2324100  Gümüşsoy, S., Hortu, İ., Alp Dal, N., Dönmez, S., & Ergenoğlu, A. M. (2022). Quality of Life and Perceived Social Support Before and After Sex Reassignment Surgery. *Clinical nursing research*, *31*(3), 481-488. https://doi.org/10.1177/10547738211040636  Ho, T. F., Zenger, B., Mark, B., Hiatt, L., Sullivan, E., Steinberg, B. A., Lyons, A., Spivak, A. M., Agarwal, C., Adelman, M., Hotaling, J., Kiraly, B., & Talboys, S. (2024). Characteristics of a transgender and gender-diverse patient population in Utah: Use of electronic health records to advance clinical and health equity research. *PloS one*, *19*(5), Article e0302895. https://doi.org/10.1371/journal.pone.0302895  Kahalon, R., Haessler, T., & Eisner, L. (2024). Self-Objectification Endorsement Among Heterosexual and Sexual Minority People and Its Association With Negative Affect and Substance Use. *Psychology of Sexual Orientation and Gender Diversity*. https://doi.org/10.1037/sgd0000701  Katz-Wise, S. L., Ranker, L. R., Korkodilos, R., Conti, J., Nelson, K. M., Xuan, Z., & Gordon, A. R. (2024). Will all youth answer sexual orientation and gender-related survey questions? An analysis of missingness in a large US survey of adolescents and young adults. *Psychological Methods*. https://doi.org/10.1037/met0000652  Kelly, N. K., Rosso, M. T., Rainer, C., Claude, K., Muessig, K. E., & Hightow-Weidman, L. (2024). Discordance between HIV risk perception, sexual behavior, and pre-exposure prophylaxis adherence among young sexual and gender minorities in the United States. *Journal of Adolescent Health*, *74*(6), 1112-1117. https://doi.org/10.1016/j.jadohealth.2024.02.028  Lyons, C., Stahlman, S., Holland, C., Ketende, S., Van Lith, L., Kochelani, D., Mavimbela, M., Sithole, B., Maloney, L., Maziya, S., & Baral, S. (2019). Stigma and outness about sexual behaviors among cisgender men who have sex with men and transgender women in Eswatini: a latent class analysis. *BMC infectious diseases*, *19*(1), 211. https://doi.org/10.1186/s12879-019-3711-2  Macapagal, K., Bhatia, R., & Greene, G. J. (2016). Differences in healthcare access, use, and experiences within a community sample of racially diverse lesbian, gay, bisexual, transgender, and questioning emerging adults. *LGBT Health*, *3*(6), 434-442. https://doi.org/10.1089/lgbt.2015.0124  Marshall, J., Zhang, X. Y., & Green, B. B. (2023). Adults' willingness to report sexual orientation and gender identity when registering for a digital health application: A cross-sectional quantitative study. *PloS one*, *18*(11), Article e0292739. https://doi.org/10.1371/journal.pone.0292739  McCurdy, A. L., Renley, B. M., Lavner, J. A., Meslay, G., Watson, R. J., & Russell, S. T. (2023). Sexual minority youth in romantic relationships: Associations with youth well-being. *JOURNAL OF RESEARCH ON ADOLESCENCE*, *33*(4), 1368-1376. https://doi.org/10.1111/jora.12883  McGorray, E. L., Finkel, E. J., & Feinstein, B. A. (2023). Bi+ identity visibility and well-being in the context of romantic relationships. *Psychology of Sexual Orientation and Gender Diversity*. https://doi.org/10.1037/sgd0000628  McRee, A.-L., Gower, A. L., & Reiter, P. L. (2018). Preventive healthcare services use among transgender young adults. *International Journal of Transgenderism*, *19*(4), 417-423. https://doi.org/10.1080/15532739.2018.1470593  Meier, S. C., Sharp, C., Michonski, J., Babcock, J. C., & Fitzgerald, K. (2013). Romantic relationships of female-to-male trans men: A descriptive study. *International Journal of Transgenderism*, *14*(2), 75-85. https://doi.org/10.1080/15532739.2013.791651  Messinger, A. M., Guadalupe-Diaz, X. L., & Kurdyla, V. (2022). Transgender Polyvictimization in the U.S. Transgender Survey. *Journal of Interpersonal Violence*, *37*(19-20), NP18810-NP18836. https://doi.org/10.1177/08862605211039250  Moseson, H., Fix, L., Ragosta, S., Forsberg, H., Hastings, J., Stoeffler, A., Lunn, M. R., Flentje, A., Capriotti, M. R., Lubensky, M. E., & Obedin-Maliver, J. (2021). Abortion experiences and preferences of transgender, nonbinary, and gender-expansive people in the United States. *American journal of obstetrics and gynecology*, *224*(4), 376.e371-376.e311. https://doi.org/10.1016/j.ajog.2020.09.035  Mustanski, B., Ryan, D. T., Newcomb, M. E., D'Aquila, R. T., & Matson, M. (2020). Very high HIV incidence and associated risk factors in a longitudinal cohort study of diverse adolescent and young adult men who have sex with men and transgender women. *AIDS and Behavior*, *24*(6), 1966-1975. https://doi.org/10.1007/s10461-019-02766-4  Nanthaprut, P., Manojai, N., Chanlearn, P., Mattawanon, N., Chiawkhun, P., Homkham, N., & Traisathit, P. (2021). Comparison of HIV-positive incidence among transgender women and men who have sex with men at stand-alone and mobile voluntary counseling and testing facilities in Chiang Mai Province, Thailand. *AIDS Patient Care and STDs*, *35*(4), 116-125. https://doi.org/10.1089/apc.2020.0258  Phillips, G., II, Raman, A., Felt, D., Han, Y., & Mustanski, B. (2019). Factors associated with PrEP support and disclosure among YMSM and transgender individuals assigned male at birth in Chicago. *AIDS and Behavior*, *23*(10), 2749-2760. https://doi.org/10.1007/s10461-019-02561-1  Resnick, D., Morales, K., Gross, R., Petsis, D., Fiore, D., Davis-Vogel, A., Metzger, D., Frank, I., & Wood, S. (2021). Prior sexually transmitted infection and human immunodeficiency virus risk perception in a diverse at-risk population of men who have sex with men and transgender individuals. *AIDS Patient Care and STDs*, *35*(1), 15-22. https://doi.org/10.1089/apc.2020.0179  Seay, J., Ranck, A., Weiss, R., Salgado, C., Fein, L., & Kobetz, E. (2017). Understanding transgender men's experiences with and preferences for cervical cancer screening: A rapid assessment survey. *LGBT Health*, *4*(4), 304-309. https://doi.org/10.1089/lgbt.2016.0143  Simpson, P. L., Callander, D., Haire, B., Pony, M., Rosenberg, S., Duck-Chong, L., Holt, M., & Cook, T. (2024). Factors Associated with Transgender and Gender Diverse People's Experience of Sexual Coercion, and Help-Seeking and Wellbeing Among Victims/Survivors: Results of the First Australian Trans and Gender Diverse Sexual Health Survey. *LGBT Health*, *11*(5), 370-381. https://doi.org/10.1089/lgbt.2023.0146  Skaletz-Rorowski, A., Potthoff, A., Nambiar, S., Basilowski, M., Wach, J., Kayser, A., Kasper, A., & Brockmeyer, N. H. (2022). Online HIV/STI Risk Test (ORT): A prospective cross-sectional study among sexually active individuals in Germany. *Journal der Deutschen Dermatologischen Gesellschaft = Journal of the German Society of Dermatology : JDDG*, *20*(3), 306-314. https://doi.org/10.1111/ddg.14674  Skorska, M. N., Coome, L. A., Peragine, D. E., Aitken, M., & VanderLaan, D. P. (2021). An anthropometric study of sexual orientation and gender identity in Thailand. *Scientific reports*, *11*(1), 18432. https://doi.org/10.1038/s41598-021-97845-9  Sun, C. J., Reboussin, B., Mann, L., Garcia, M., & Rhodes, S. D. (2016). The HIV risk profiles of latino sexual minorities and transgender persons who use websites or apps designed for social and sexual networking. *Health Education & Behavior*, *43*(1), 86-93. https://doi.org/10.1177/1090198115596735  Tanner, A. E., Song, E. Y., Mann-Jackson, L., Alonzo, J., Schafer, K., Ware, S., Garcia, J. M., Hall, E. A., Bell, J. C., Van Dam, C. N., & Rhodes, S. D. (2018). Preliminary impact of the weCare social media intervention to support health for young men who have sex with men and transgender women with HIV. *AIDS Patient Care and STDs*, *32*(11), 450-458. https://doi.org/10.1089/apc.2018.0060  Thompson, H. M., Rusie, L. K., Schneider, J. A., & Mehta, S. D. (2024). Bacterial vaginosis testing gaps for transmasculine patients may exacerbate health disparities. *FRONTIERS IN REPRODUCTIVE HEALTH*, *6*, Article 1344111. https://doi.org/10.3389/frph.2024.1344111  Tillewein, H., Brashear, B., & Harvey, P. (2022). The link between centrality, gender identity, and sexual pleasure. *SEXOLOGIES*, *31*(4), 374-379. https://doi.org/10.1016/j.sexol.2022.09.005  Tomada, I., Tomada, N., Almeida, H., & Neves, D. (2013). Androgen depletion in humans leads to cavernous tissue reorganization and upregulation of Sirt1-eNOS axis. *Age (Dordrecht, Netherlands)*, *35*(1), 35-47. https://doi.org/10.1007/s11357-011-9328-z  Tordoff, D. M., Dombrowski, J. C., Ramchandani, M. S., & Barbee, L. A. (2022). Trans-inclusive Sexual Health Questionnaire to Improve HIV/STI Care for Transgender Patients: Anatomic-site Specific STI Prevalence & Screening Rates. *Clinical infectious diseases : an official publication of the Infectious Diseases Society of America*. https://doi.org/10.1093/cid/ciac370  Tordoff, D. M., Lunn, M. R., Chen, B. R., Flentje, A., Dastur, Z., Lubensky, M. E., Capriotti, M., & Obedin-Maliver, J. (2023). Testosterone use and sexual function among transgender men and gender diverse people assigned female at birth. *American journal of obstetrics and gynecology*, *229*(6), e1-e17. https://doi.org/10.1016/j.ajog.2023.08.035  van der Sluis, W. B., Steensma, T. D., Timmermans, F. W., Smit, J. M., de Haseth, K., Özer, M., & Bouman, M.-B. (2020). Gender-confirming vulvoplasty in transgender women in the Netherlands: Incidence, motivation analysis, and surgical outcomes. *Journal of Sexual Medicine*, *17*(8), 1566-1573. https://doi.org/10.1016/j.jsxm.2020.04.007  Van Schuylenbergh, J., Motmans, J., Defreyne, J., Somers, A., & T'Sjoen, G. (2019). Sexual health, transition-related risk behavior and need for health care among transgender sex workers. *International Journal of Transgenderism*, *20*(4), 388-402. https://doi.org/10.1080/15532739.2019.1617217  Watson, D. L., Listerud, L., Drab, R. A., Lin, W. Y., Momplaisir, F. M., & Bauermeister, J. A. (2024). HIV pre-exposure prophylaxis programme preferences among sexually active HIV-negative transgender and gender diverse adults in the United States: a conjoint analysis. *Journal of the International AIDS Society*, *27*(2), Article e26211. https://doi.org/10.1002/jia2.26211  Whitton, S. W., Dyar, C., Newcomb, M. E., & Mustanski, B. (2018). Effects of romantic involvement on substance use among young sexual and gender minorities. *Drug and Alcohol Dependence*, *191*, 215-222. https://doi.org/10.1016/j.drugalcdep.2018.06.037  Whitton, S. W., Godfrey, L. M., Crosby, S., & Newcomb, M. E. (2020). Romantic involvement and mental health in sexual and gender minority emerging adults assigned female at birth. *Journal of Social and Personal Relationships*, *37*(4), 1340-1361. https://doi.org/10.1177/0265407519898000  Zhang, Z., Smith-Johnson, M., & Tumin, D. (2024). Contextual Influences on Nonresponse to Health Survey Questions About Sexual Orientation and Gender Identity. *LGBT Health*, *11*(1), 66-73. https://doi.org/10.1089/lgbt.2022.0320 |
| **Mean age sample < 16** | Bőthe, B., Vaillancourt-Morel, M.-P., Girouard, A., Štulhofer, A., Dion, J., & Bergeron, S. (2020). A Large-Scale Comparison of Canadian Sexual/Gender Minority and Heterosexual, Cisgender Adolescents' Pornography Use Characteristics. *The journal of sexual medicine*, *17*(6), 1156-1167. https://doi.org/10.1016/j.jsxm.2020.02.009  Bungener, S. L., Steensma, T. D., Cohen-Kettenis, P. T., & de Vries, A. L. C. (2017). Sexual and romantic experiences of transgender youth before gender-affirmative treatment. *Pediatrics*, *139*(3), 1-9. https://search.ebscohost.com/login.aspx?direct=true&db=psyh&AN=2017-22997-004&site=ehost-live  s.bungener@vumc.nl  Girouard, A., Dion, J., Bőthe, B., O'Sullivan, L., & Bergeron, S. (2021). Bullying victimization and sexual wellbeing in sexually active heterosexual, cisgender and sexual/gender minority adolescents: The mediating role of emotion regulation. *Journal of Youth and Adolescence*, *50*(11), 2136-2150. https://doi.org/10.1007/s10964-021-01471-7  Logie, C. H., Lys, C. L., Mackay, K., MacNeill, N., Pauchulo, A., & Yasseen, A. S., III. (2019). Syndemic factors associated with safer sex efficacy among northern and indigenous adolescents in Arctic Canada. *International Journal of Behavioral Medicine*, *26*(4), 449-453. https://doi.org/10.1007/s12529-019-09797-0  McKenna, J. L., Vu, A., McGregor, K., Williams, C. R., Rana, V., & Boskey, E. R. (2024). Sexual Orientation Labels Used by Transgender and Gender Diverse Adolescents and Young Adults Seeking Gender Affirming Hormones. *SEXUALITY & CULTURE-AN INTERDISCIPLINARY JOURNAL*. https://doi.org/10.1007/s12119-024-10216-0  Paquette, M.-M., Dion, J., Bőthe, B., Girouard, A., & Bergeron, S. (2022). Heterosexual, cisgender and gender and sexually diverse adolescents’ sexting behaviors: The role of body appreciation. *Journal of Youth and Adolescence*, *51*(2), 278-290. https://doi.org/10.1007/s10964-021-01568-z  Ristori, J., Rossi, E., Cocchetti, C., Mazzoli, F., Castellini, G., Vignozzi, L., Ricca, V., Maggi, M., & Fisher, A. D. (2021). Sexual habits among Italian transgender adolescents: a cross-sectional study. *International Journal of Impotence Research*, *33*(7), 687-693. https://doi.org/10.1038/s41443-021-00427-6  Szoko, N., Sequeira, G. M., Coulter, R. W. S., Kobey, J., Ridenour, E., Burnett, O., & Kidd, K. M. (2023). Sexual Orientation Among Gender Diverse Youth. *Journal of Adolescent Health*, *72*(1), 153-155. https://doi.org/10.1016/j.jadohealth.2022.08.016 |
| **Published before 2012** | Barr, R. F., Raphael, B., & Hennessey, N. (1974). Apparent heterosexuality in two male patients requesting change-of-sex operation. *ARCHIVES OF SEXUAL BEHAVIOR*, *3*(4), 325-330. https://doi.org/10.1007/BF01636438  Barrett, J. (1998). Psychological and social function before and after phalloplasty. *INTERNATIONAL JOURNAL OF TRANSGENDERISM*, *2*(1). https://search.ebscohost.com/login.aspx?direct=true&db=psyh&AN=2001-03117-003&site=ehost-live  Bedard, C., Zhang, H. L., & Zucker, K. J. (2010). Gender identity and sexual orientation in people with developmental disabilities. *SEXUALITY AND DISABILITY*, *28*(3), 165-175. https://doi.org/10.1007/s11195-010-9155-7  Berg, R., & Berg, G. (1983). Penile malformation, gender identity and sexual orientation. *Acta Psychiatrica Scandinavica*, *68*(3), 154-166. https://doi.org/10.1111/j.1600-0447.1983.tb06995.x  Blanchard, R. (1985). Typology of male-to-female transsexualism. *ARCHIVES OF SEXUAL BEHAVIOR*, *14*(3), 247-261. https://doi.org/10.1007/BF01542107  Blanchard, R. (1989). The concept of autogynephilia and the typology of male gender dysphoria. *Journal of Nervous and Mental Disease*, *177*(10), 616-623. https://doi.org/10.1097/00005053-198910000-00004  Blanchard, R. (1993). Partial versus complete autogynephilia and gender dysphoria. *JOURNAL OF SEX & MARITAL THERAPY*, *19*(4), 301-307. https://doi.org/10.1080/00926239308404373  Blanchard, R. (1993). Varieties of autogynephilia and their relationship to gender dysphoria. *ARCHIVES OF SEXUAL BEHAVIOR*, *22*(3), 241-251. https://doi.org/10.1007/BF01541769  Blanchard, R., & Clemmensen, L. H. (1988). A test of the DSM-III—R's implicit assumption that fetishistic arousal and gender dysphoria are mutually exclusive. *JOURNAL OF SEX RESEARCH*, *25*(3), 426-432. https://doi.org/10.1080/00224498809551472  Blanchard, R., Legault, S., & Lindsay, W. R. (1987). Vaginoplasty outcome in male-to-female transsexuals. *JOURNAL OF SEX & MARITAL THERAPY*, *13*(4), 265-275. https://doi.org/10.1080/00926238708403899  Blanchard, R., Racansky, I. G., & Steiner, B. W. (1986). Phallometric detection of fetishistic arousal in heterosexual male cross-dressers. *JOURNAL OF SEX RESEARCH*, *22*(4), 452-462. https://doi.org/10.1080/00224498609551326  Bockting, W., Huang, C.-Y., Ding, H., Robinson, B., & Rosser, B. R. S. (2005). Are transgender persons at higher risk for HIV than other sexual minorities? A comparison of HIV prevalence and risks. *INTERNATIONAL JOURNAL OF TRANSGENDERISM*, *8*(2-3), 123-131. https://doi.org/10.1300/J485v08n02_11  Bockting, W. O., Robinson, B. E., Forberg, J., & Scheltema, K. (2005). Evaluation of a sexual health approach to reducing HIV/STD risk in the transgender community. *AIDS Care*, *17*(3), 289-303. https://doi.org/10.1080/09540120412331299825  Bowers, J. R., Branson, C. M., Fletcher, J., & Reback, C. J. (2011). Differences in substance use and sexual partnering between men who have sex with men, men who have sex with men and women and transgender women. *Culture, Health & Sexuality*, *13*(6), 629-642. https://doi.org/10.1080/13691058.2011.564301  Brotto, L. A., Gehring, D., Klein, C., Gorzalka, B. B., Thomson, S., & Knudson, G. (2005). Psychophysiological and subjective sexual arousal to visual sexual stimuli in new women. *Journal of Psychosomatic Obstetrics & Gynecology*, *26*(4), 237-244. https://doi.org/10.1080/01443610400023171  Brown, G. R., Wise, T. N., Costa, P. T., Herbst, J. H., Fagan, P. J., & Schmidt, C. W. (1996). Personality characteristics and sexual functioning of 188 cross-dressing men. *Journal of Nervous and Mental Disease*, *184*(5), 265-273. https://doi.org/10.1097/00005053-199605000-00001  Buhrich, N., & Beaumont, T. (1981). Comparison of transvestism in Australia and America. *ARCHIVES OF SEXUAL BEHAVIOR*, *10*(3), 269-279. https://doi.org/10.1007/BF01543079  Buhrich, N., & McConaghy, N. (1977). The discrete syndromes of transvestism and transsexualism. *ARCHIVES OF SEXUAL BEHAVIOR*, *6*(6), 483-495. https://doi.org/10.1007/BF01541153  Buhrich, N., & McConaghy, N. (1978). Two clinically discrete syndromes of transsexualism. *The British Journal of Psychiatry*, *133*, 73-76. https://doi.org/10.1192/bjp.133.1.73  Bullough, B., & Bullough, V. (1997). Are transvestites necessarily heterosexual? *ARCHIVES OF SEXUAL BEHAVIOR*, *26*(1), 1-12. https://doi.org/10.1023/A:1024589618410  Bullough, V., Bullough, B., & Smith, R. (1983). A comparative study of male transvestites, male to female transsexuals, and male homosexuals. *JOURNAL OF SEX RESEARCH*, *19*(3), 238-257. https://doi.org/10.1080/00224498309551185  Chariyalertsak, S., Kosachunhanan, N., Saokhieo, P., Songsupa, R., Wongthanee, A., Chariyalertsak, C., Visarutratana, S., & Beyrer, C. (2011). HIV incidence, risk factors, and motivation for biomedical intervention among gay, bisexual men, and transgender persons in Northern Thailand. *PLOS ONE*, *6*(9), e24295. https://doi.org/10.1371/journal.pone.0024295  Chemnasiri, T., Netwong, T., Visarutratana, S., Varangrat, A., Li, A., Phanuphak, P., Jommaroeng, R., Akarasewi, P., & van Griensven, F. (2010). Inconsistent condom use among young men who have sex with men, male sex workers, and transgenders in Thailand. *AIDS EDUCATION AND PREVENTION*, *22*(2), 100-109. https://doi.org/10.1521/aeap.2010.22.2.100  Chivers, M. L., & Bailey, J. M. (2000). Sexual orientation of female-to-male transsexuals: A comparison of homosexual and nonhomosexual types. *ARCHIVES OF SEXUAL BEHAVIOR*, *29*(3), 259-278. https://doi.org/10.1023/A:1001915530479  Chivers, M. L., Rieger, G., Latty, E., & Bailey, J. M. (2004). A sex difference in the specificity of sexual arousal. *Psychological Science*, *15*(11), 736-744. https://doi.org/10.1111/j.0956-7976.2004.00750.x  Clare, D., & Tully, B. (1989). Transhomosexuality, or the dissociation of sexual orientation and sex object choice. *ARCHIVES OF SEXUAL BEHAVIOR*, *18*(6), 531-536. https://doi.org/10.1007/BF01541679  Clerkin, E. M., Newcomb, M. E., & Mustanski, B. (2011). Unpacking the racial disparity in HIV rates: The effect of race on risky sexual behavior among Black young men who have sex with men (YMSM). *Journal of Behavioral Medicine*, *34*(4), 237-243. https://doi.org/10.1007/s10865-010-9306-4  Cohen-Ketteinis, P. T., & van Goozen, S. H. M. (1997). Sex reassignment of adolescent transsexuals: A follow-up study. *Journal of the American Academy of Child & Adolescent Psychiatry*, *36*(2), 263-271. https://doi.org/10.1097/00004583-199702000-00017  Coleman, E., Bockting, W. O., & Gooren, L. (1993). Homosexual and bisexual identity in sex-reassigned female-to-male transsexuals. *ARCHIVES OF SEXUAL BEHAVIOR*, *22*(1), 37-50. https://doi.org/10.1007/BF01552911  Cook-Daniels, L., & Munson, M. (2010). Sexual violence, elder abuse, and sexuality of transgender adults, age 50+: Results of three surveys. *JOURNAL OF GLBT FAMILY STUDIES*, *6*(2), 142-177. https://doi.org/10.1080/15504281003705238  Daskalos, C. T. (1998). Changes in the sexual orientation of six heterosexual male-to-female transsexuals. *ARCHIVES OF SEXUAL BEHAVIOR*, *27*(6), 605-614. https://doi.org/10.1023/A:1018725201811  De Cuypere, G., T'Sjoen, G., Beerten, R., Selvaggi, G., De Sutter, P., Hoebeke, P., Monstrey, S., Vansteenwegen, A., & Rubens, R. (2005). Sexual and Physical Health After Sex Reassignment Surgery. *ARCHIVES OF SEXUAL BEHAVIOR*, *34*(6), 679-690. https://doi.org/10.1007/s10508-005-7926-5  Derogatis, L. R., Meyer, J. K., & Boland, P. (1981). A psychological profile of the transsexual. II. The female. *The Journal of nervous and mental disease*, *169*(3), 157-168. https://doi.org/10.1097/00005053-198103000-00002  Derogatis, L. R., Meyer, J. K., & Vazquez, N. (1978). A psychological profile of the transsexual: I The male. *Journal of Nervous and Mental Disease*, *166*(4), 234-254. https://doi.org/10.1097/00005053-197804000-00002  Djordjevic, M. L., Stanojevic, D. S., & Bizic, M. R. (2011). Rectosigmoid vaginoplasty: Clinical experience and outcomes in 86 cases. *JOURNAL OF SEXUAL MEDICINE*, *8*(12), 3487-3494. https://doi.org/10.1111/j.1743-6109.2011.02494.x  Docter, R. F., & Prince, V. (1997). Transvestism: A survey of 1032 cross-dressers. *ARCHIVES OF SEXUAL BEHAVIOR*, *26*(6), 589-605. https://doi.org/10.1023/A:1024572209266  Doorn, C. D., Poortinga, J., & Verschoor, A. M. (1994). Cross-gender identity in transvestites and male transsexuals. *ARCHIVES OF SEXUAL BEHAVIOR*, *23*(2), 185-201. https://doi.org/10.1007/BF01542098  Doornaert, M., Hoebeke, P., Ceulemans, P., T'Sjoen, G., Heylens, G., & Monstrey, S. (2011). Penile reconstruction with the radial forearm flap: an update. *Handchirurgie, Mikrochirurgie, plastische Chirurgie : Organ der Deutschsprachigen Arbeitsgemeinschaft fur Handchirurgie : Organ der Deutschsprachigen Arbeitsgemeinschaft fur Mikrochirurgie der Peripheren Nerven und Gefasse : Organ der V.. 43*(4), 208-214. https://doi.org/10.1055/s-0030-1267215  Elaut, E., De Cuypere, G., De Sutter, P., Gijs, L., Van Trotsenburg, M., Heylens, G., Kaufman, J.-M., Rubens, R., & T'Sjoen, G. (2008). Hypoactive sexual desire in transsexual women: prevalence and association with testosterone levels. *EUROPEAN JOURNAL OF ENDOCRINOLOGY*, *158*(3), 393-399. https://doi.org/10.1530/EJE-07-0511  Fagan, P. J., Wise, T. N., Derogatis, L. R., & Schmidt, C. W. (1988). Distressed transvestites: Psychometric characteristics. *Journal of Nervous and Mental Disease*, *176*(10), 626-632. https://doi.org/10.1097/00005053-198810000-00008  Fleming, M., MacGowan, B., & Costos, D. (1985). The dyadic adjustment of female-to-male transsexuals. *ARCHIVES OF SEXUAL BEHAVIOR*, *14*(1), 47-55. https://doi.org/10.1007/BF01541352  Fleming, M. Z., MacGowan, B. R., & Salt, P. (1984). Female-to-male transsexualism and sex roles: self and spouse ratings on the PAQ. *ARCHIVES OF SEXUAL BEHAVIOR*, *13*(1), 51-57. https://doi.org/10.1007/BF01542977  Forrest, B. (1995). THE SEXUAL HEALTH OF INDIGENOUS GAY MEN AND TRANSGENDER PEOPLE. *VENEREOLOGY-THE INTERDISCIPLINARY INTERNATIONAL JOURNAL OF SEXUAL HEALTH*, *8*(1), 13-14. <Go to ISI>://WOS:A1995QQ34600004  Freund, K., Seto, M. C., & Kuban, M. (1996). Two types of fetishism. *Behaviour Research and Therapy*, *34*(9), 687-694. https://doi.org/10.1016/0005-7967(96)00047-2  Fulcheri, M., Bertone, E., & Barzega, G. (1995). [Comments relating to the psychosexual characteristics of the male transsexual. A clinical study]. *Minerva Psichiatrica*, *36*(1), 11-18. https://search.ebscohost.com/login.aspx?direct=true&db=cmedm&AN=7643729&site=ehost-live  Gizewski, E. R., Krause, E., Schlamann, M., Happich, F., Ladd, M. E., Forsting, M., & Senf, W. (2009). Specific cerebral activation due to visual erotic stimuli in male-to-female transsexuals compared with male and female controls: An fMRI study. *JOURNAL OF SEXUAL MEDICINE*, *6*(2), 440-448. https://doi.org/10.1111/j.1743-6109.2008.00981.x  Godano, A., Massara, D., Crovella, U., Brigatti, L., Cocimano, V., & Marten Perolino, R. (1990). [Follow-up of surgically treated transsexuals: what has changed?]. *Archivio italiano di urologia, nefrologia, andrologia : organo ufficiale dell'Associazione per la ricerca in urologia = Urological, nephrological, and andrological sciences*, *62*(1), 113-116. https://search.ebscohost.com/login.aspx?direct=true&db=cmedm&AN=2141703&site=ehost-live  Green, R. (1979). Childhood cross-gender behavior and subsequent sexual preference. *The American Journal of Psychiatry*, *136*(1), 106-108. https://doi.org/10.1176/ajp.136.1.106  Greenblatt, R. B., Jungck, E. C., & Blum, H. (1972). Endocrinology of sexual behavior. *Medical Aspects of Human Sexuality*, *6*(1), 110-131. https://search.ebscohost.com/login.aspx?direct=true&db=psyh&AN=1973-09614-001&site=ehost-live  Grimm, D. E. (1987). Toward a theory of gender: Transsexualism, gender, sexuality, and relationships. *AMERICAN BEHAVIORAL SCIENTIST*, *31*(1), 66-85. https://doi.org/10.1177/000276487031001005  Guadamuz, T. E., Wimonsate, W., Varangrat, A., Phanuphak, P., Jommaroeng, R., McNicholl, J. M., Mock, P. A., Tappero, J. W., & van Griensven, F. (2011). HIV prevalence, risk behavior, hormone use and surgical history among transgender persons in Thailand. *AIDS AND BEHAVIOR*, *15*(3), 650-658. https://doi.org/10.1007/s10461-010-9850-5  Hastings, D. W. (1974). Postsurgical adjustment of male transsexual patients. *CLINICS IN PLASTIC SURGERY*, *1*(2), 335-344. https://search.ebscohost.com/login.aspx?direct=true&db=cmedm&AN=4473316&site=ehost-live  Heintz, A. J., & Melendez, R. M. (2006). Intimate Partner Violence and HIV/STD Risk Among Lesbian, Gay, Bisexual, and Transgender Individuals. *JOURNAL OF INTERPERSONAL VIOLENCE*, *21*(2), 193-208. https://doi.org/10.1177/0886260505282104  Hill, S. C., Daniel, J., Benzie, A., Ayres, J., King, G., & Smith, A. (2011). Sexual health of transgender sex workers attending an inner-city genitourinary medicine clinic. *INTERNATIONAL JOURNAL OF STD & AIDS*, *22*(11), 686-687. https://doi.org/10.1258/ijsa.2009.009491  Hines, M. (2004). Psychosexual development in individuals who have female pseudohermaphroditism. *CHILD AND ADOLESCENT PSYCHIATRIC CLINICS OF NORTH AMERICA*, *13*(3), 641-656. https://doi.org/10.1016/j.chc.2004.02.013  Hines, M., Ahmed, S. F., & Hughes, I. A. (2003). Psychological outcomes and gender-related development in complete androgen insensitivity syndrome. *ARCHIVES OF SEXUAL BEHAVIOR*, *32*(2), 93-101. https://doi.org/10.1023/A:1022492106974  Hounsfield, V. L., Freedman, E., McNulty, A., & Bourne, C. (2007). Transgender people attending a Sydney sexual health service over a 16-year period. *SEXUAL HEALTH*, *4*(3), 189-193. https://doi.org/10.1071/sh07020  Hunt, D. D., & Hampson, J. L. (1980). Follow-up of 17 biologic male transsexuals after sex-reassignment surgery. *The American Journal of Psychiatry*, *137*(4), 432-438. https://doi.org/10.1176/ajp.137.4.432  Imbimbo, C., Verze, P., Palmieri, A., Longo, N., Fusco, F., Arcaniolo, D., & Mirone, V. (2009). A report from a single institute's 14-year experience in treatment of male-to-female transsexuals. *JOURNAL OF SEXUAL MEDICINE*, *6*(10), 2736-2745. https://doi.org/10.1111/j.1743-6109.2009.01379.x  Jarolím, L. (2000). Surgical conversion of genitalia in transsexual patients. *BJU international*, *85*(7), 851-856. https://doi.org/10.1046/j.1464-410x.2000.00624.x  Jarolím, L., Šedý, J., Schmidt, M., Ondřej, N., Foltán, R., & Kawaciuk, I. (2009). Gender reassignment surgery in male-to-female transsexualism: A retrospective 3-month follow-up study with anatomical remarks. *JOURNAL OF SEXUAL MEDICINE*, *6*(6), 1635-1644. https://doi.org/10.1111/j.1743-6109.2009.01245.x  Khan, A. A., Rehan, N., Qayyum, K., & Khan, A. (2008). Correlates and prevalence of HIV and sexually transmitted infections among Hijras (male transgenders) in Pakistan. *INTERNATIONAL JOURNAL OF STD & AIDS*, *19*(12), 817-820. https://doi.org/10.1258/ijsa.2008.008135  Kins, E., Hoebeke, P., Heylens, G., Rubens, R., & de Cuypere, G. (2008). The female-to-male transsexual and his female partner versus the traditional couple: A comparison. *JOURNAL OF SEX & MARITAL THERAPY*, *34*(5), 429-438. https://doi.org/10.1080/00926230802156236  Krege, S., Bex, A., Lümmen, G., & Rübben, H. (2001). Male-to-female transsexualism: a technique, results and long-term follow-up in 66 patients. *BJU international*, *88*(4), 396-402. https://doi.org/10.1046/j.1464-410x.2001.02323.x  Kronawitter, D., Gooren, L. J., Zollver, H., Oppelt, P. G., Beckmann, M. W., Dittrich, R., & Mueller, A. (2009). Effects of transdermal testosterone or oral dydrogesterone on hypoactive sexual desire disorder in transsexual women: results of a pilot study. *EUROPEAN JOURNAL OF ENDOCRINOLOGY*, *161*(2), 363-368. https://doi.org/10.1530/EJE-09-0265  Kuhn, A., Santi, A., & Birkhäuser, M. (2011). Vaginal prolapse, pelvic floor function, and related symptoms 16 years after sex reassignment surgery in transsexuals. *FERTILITY AND STERILITY*, *95*(7), 2379-2382. https://doi.org/10.1016/j.fertnstert.2011.03.029  Kwan, M., VanMaasdam, J., & Davidson, J. M. (1985). Effects of estrogen treatment on sexual behavior in male-to-female transsexuals: Experimental and clinical observations. *ARCHIVES OF SEXUAL BEHAVIOR*, *14*(1), 29-40. https://doi.org/10.1007/BF01541350  Large, M. C., Gottlieb, L. J., Wille, M. A., DeWolfe, M., & Bales, G. T. (2009). Novel technique for proximal anchoring of penile prostheses in female-to-male transsexual. *UROLOGY*, *74*(2), 419-421. https://doi.org/10.1016/j.urology.2009.01.044  Lawrence, A. A. (2003). Factors associated with satisfaction or regret following male-to-female sex reassignment surgery. *ARCHIVES OF SEXUAL BEHAVIOR*, *32*(4), 299-315. https://doi.org/10.1023/A:1024086814364  Lawrence, A. A. (2005). Sexuality Before and After Male-to-Female Sex Reassignment Surgery. *ARCHIVES OF SEXUAL BEHAVIOR*, *34*(2), 147-166. https://doi.org/10.1007/s10508-005-1793-y  Lawrence, A. A. (2006). Patient-reported complications and functional outcomes of male-to-female sex reassignment surgery. *ARCHIVES OF SEXUAL BEHAVIOR*, *35*(6), 717-727. https://doi.org/10.1007/s10508-006-9104-9  Lawrence, A. A. (2010). Societal individualism predicts prevalence of nonhomosexual orientation in male-to-female transsexualism. *ARCHIVES OF SEXUAL BEHAVIOR*, *39*(2), 573-583. https://doi.org/10.1007/s10508-008-9420-3  Lawrence, A. A., Latty, E. M., Chivers, M. L., & Bailey, J. M. (2005). Measurement of Sexual Arousal in Postoperative Male-to-Female Transsexuals Using Vaginal Photoplethysmography. *ARCHIVES OF SEXUAL BEHAVIOR*, *34*(2), 135-145. https://doi.org/10.1007/s10508-005-1792-z  Lief, H. I., & Hubschman, L. (1993). Orgasm in the postoperative transsexual. *ARCHIVES OF SEXUAL BEHAVIOR*, *22*(2), 145-155. https://doi.org/10.1007/BF01542363  Lindemalm, G., Körlin, D., & Uddenberg, N. (1986). Long-term follow-up of 'sex change' in 13 male-to-female transsexuals. *ARCHIVES OF SEXUAL BEHAVIOR*, *15*(3), 187-210. https://doi.org/10.1007/BF01542412  Lobato, M. I. I., Koff, W. J., Manenti, C., da Fonseca Seger, D., Salvador, J., da Graça Borges Fortes, M., Petry, A. R., Silveira, E., & Henriques, A. A. (2006). Follow-up of sex reassignment surgery in transsexuals: A Brazilian cohort. *ARCHIVES OF SEXUAL BEHAVIOR*, *35*(6), 711-715. https://doi.org/10.1007/s10508-006-9074-y  Löwenberg, H., Lax, H., Neto, R. R., & Krege, S. (2010). Komplikationen, subjektive zufriedenheit und sexuelles erieben nach geschlechtsangleichender operation bei mann-zu-frau-transsexualität = Complications, subjective satisfaction and experience of sexuality following sex reassignment surgery in the case of male-to-female transsexuality. *ZEITSCHRIFT FUR SEXUALFORSCHUNG*, *23*(4), 328-347. https://doi.org/10.1055/s-0030-1262718  Lumen, N., Monstrey, S., Selvaggi, G., Ceulemans, P., De Cuypere, G., Van Laecke, E., & Hoebeke, P. (2008). Phalloplasty: a valuable treatment for males with penile insufficiency. *UROLOGY*, *71*(2), 272-276. https://doi.org/10.1016/j.urology.2007.08.066  McCauley, E. A., & Ehrhardt, A. A. (1980). Sexual behavior in female transsexuals and lesbians. *JOURNAL OF SEX RESEARCH*, *16*(3), 202-211. https://doi.org/10.1080/00224498009551077  Modan, B., Goldschmidt, R., Rubinstein, E., Vonsover, A., Zinn, M., Golan, R., Chetrit, A., & Gottlieb-Stematzky, T. (1992). Prevalence of HIV antibodies in transsexual and female prostitutes. *AMERICAN JOURNAL OF PUBLIC HEALTH*, *82*(4), 590-592. https://doi.org/10.2105/ajph.82.4.590  Nemoto, T., Luke, D., Mamo, L., Ching, A., & Patria, J. (1999). HIV risk behaviours among male-to-female transgenders in comparison with homosexual or bisexual males and heterosexual females. *AIDS Care*, *11*(3), 297-312. https://doi.org/10.1080/09540129947938  Rakic, Z., Starcevic, V., Maric, J., & Kelin, K. (1996). The outcome of sex reassignment surgery in Belgrade: 32 patients of both sexes. *ARCHIVES OF SEXUAL BEHAVIOR*, *25*(5), 515-525. https://doi.org/10.1007/BF02437545  Randell, J. (1975). Transvestism and trans-sexualism. *The British journal of psychiatry : the journal of mental science*, *Spec No 9*, 201-205. https://search.ebscohost.com/login.aspx?direct=true&db=cmedm&AN=1102015&site=ehost-live  Rehman, J., Lazer, S., Benet, A. E., Schaefer, L. C., & Melman, A. (1999). The reported sex and surgery satisfactions of 28 postoperative male-to-female transsexual patients. *ARCHIVES OF SEXUAL BEHAVIOR*, *28*(1), 71-89. https://doi.org/10.1023/A:1018745706354  Rehman, J., & Melman, A. (1999). Formation of neoclitoris from glans penis by reduction glansplasty with preservation of neurovascular bundle in male-to-female gender surgery: functional and cosmetic outcome. *The Journal of urology*, *161*(1), 200-206. https://search.ebscohost.com/login.aspx?direct=true&db=cmedm&AN=10037398&site=ehost-live  Reisner, S. L., Perkovich, B., & Mimiaga, M. J. (2010). Mixed methods study of the sexual health needs of New England transmen who have sex with nontransgender men. *AIDS PATIENT CARE AND STDS*, *24*(8), 501-513. https://doi.org/10.1089/apc.2010.0059  Rubin, S. O. (1993). Sex-reassignment surgery male-to-female. Review, own results and report of a new technique using the glans penis as a pseudoclitoris. *Scandinavian journal of urology and nephrology. Supplementum*, *154*, 1-28. https://search.ebscohost.com/login.aspx?direct=true&db=cmedm&AN=8140401&site=ehost-live  Schroder, M., & Carroll, R. A. (1999). New women: Sexological outcomes of male-to-female gender reassignment surgery. *Journal of Sex Education & Therapy*, *24*(3), 137-146. https://doi.org/10.1080/01614576.1999.11074293  Selvaggi, G., Monstrey, S., Ceulemans, P., T'Sjoen, G., De Cuypere, G., & Hoebeke, P. (2007). Genital sensitivity after sex reassignment surgery in transsexual patients. *ANNALS OF PLASTIC SURGERY*, *58*(4), 427-433. https://doi.org/10.1097/01.sap.0000238428.91834.be  Sevelius, J. M., Carrico, A., & Johnson, M. O. (2010). Antiretroviral therapy adherence among transgender women living with HIV. *JANAC: Journal of the Association of Nurses in AIDS Care*, *21*(3), 256-264. https://doi.org/10.1016/j.jana.2010.01.005  Simon, P. A., Reback, C. J., & Bemis, C. C. (2000). HIV prevalence and incidence among male-to-female transsexuals receiving HIV prevention services in Los Angeles County. *AIDS*, *14*(18), 2953-2955. https://doi.org/10.1097/00002030-200012220-00024  Slabbekoorn, D., Van Goozen, S. H. M., Gooren, L. J. G., & Cohen-Kettenis, P. T. (2001). Effects of cross-sex hormone treatment on emotionality in transsexuals. *INTERNATIONAL JOURNAL OF TRANSGENDERISM*, *5*(3). https://search.ebscohost.com/login.aspx?direct=true&db=psyh&AN=2001-03445-002&site=ehost-live  Smith, Y. L. S., van Goozen, S. H. M., Kuiper, A. J., & Cohen-Kettenis, P. T. (2005). Transsexual subtypes: Clinical and theoretical significance. *PSYCHIATRY RESEARCH*, *137*(3), 151-160. https://doi.org/10.1016/j.psychres.2005.01.008  Soli, M., Brunocilla, E., Bertaccini, A., Palmieri, F., Barbieri, B., & Martorana, G. (2008). Male to female gender reassignment: Modified surgical technique for creating the neoclitoris and mons veneris. *JOURNAL OF SEXUAL MEDICINE*, *5*(1), 210-216. https://doi.org/10.1111/j.1743-6109.2007.00632.x  Sørensen, T. (1981). A follow-up study of operated transsexual females. *Acta Psychiatrica Scandinavica*, *64*(1), 50-64. https://doi.org/10.1111/j.1600-0447.1981.tb00760.x  Steiner, B. W., & Bernstein, S. M. (1981). Female-to-male transsexuals and their partners. *The Canadian Journal of Psychiatry / La Revue canadienne de psychiatrie*, *26*(3), 178-182. https://search.ebscohost.com/login.aspx?direct=true&db=psyh&AN=1982-21063-001&site=ehost-live  Stephens, T., Cozza, S., & Braithwaite, R. L. (1999). Transsexual orientation in HIV risk behaviours in an adult male prison. *INTERNATIONAL JOURNAL OF STD & AIDS*, *10*(1), 28-31. https://doi.org/10.1258/0956462991913042  Szalay, L. V. (1992). CONSTRUCTION OF A NEOCLITORIS IN THE SURGERY OF MALE-TRANSSEXUALS. *EUROPEAN JOURNAL OF PLASTIC SURGERY*, *15*(4), 192-193. https://doi.org/10.1007/bf00660510  Vankemenade, J., Cohenkettenis, P. T., Cohen, L., & Gooren, L. J. G. (1989). EFFECTS OF THE PURE ANTIANDROGEN RU-23.903 (ANANDRON) ON SEXUALITY, AGGRESSION, AND MOOD IN MALE-TO-FEMALE TRANSSEXUALS. *ARCHIVES OF SEXUAL BEHAVIOR*, *18*(3), 217-228. https://doi.org/10.1007/bf01543196  Veale, J. F., Clarke, D. E., & Lomax, T. C. (2008). Sexuality of male-to-female transsexuals. *ARCHIVES OF SEXUAL BEHAVIOR*, *37*(4), 586-597. https://doi.org/10.1007/s10508-007-9306-9  Veale, J. F., Clarke, D. E., & Lomax, T. C. (2010). Biological and psychosocial correlates of adult gender-variant identities: New findings. *PERSONALITY AND INDIVIDUAL DIFFERENCES*, *49*(3), 252-257. https://doi.org/10.1016/j.paid.2010.03.045  von Szalay, L. (1990). [Construction of a neo-clitoris in male-to-female transsexuals]. *Handchirurgie, Mikrochirurgie, plastische Chirurgie : Organ der Deutschsprachigen Arbeitsgemeinschaft fur Handchirurgie : Organ der Deutschsprachigen Arbeitsgemeinschaft fur Mikrochirurgie der Peripheren Nerven und Gefasse : Organ der V.. 22*(5), 277-278. https://search.ebscohost.com/login.aspx?direct=true&db=cmedm&AN=2227646&site=ehost-live  Wagner, S., Greco, F., Hoda, M. R., Inferrera, A., Lupo, A., Hamza, A., & Fornara, P. (2010). Male-to-female transsexualism: technique, results and 3-year follow-up in 50 patients. *UROLOGIA INTERNATIONALIS*, *84*(3), 330-333. https://doi.org/10.1159/000288238  Warne, G., Grover, S., Hutson, J., Sinclair, A., Metcalfe, S., Northam, E., & Freeman, J. (2005). A long-term outcome study of intersex conditions. *Journal of pediatric endocrinology & metabolism : JPEM*, *18*(6), 555-567. https://doi.org/10.1515/jpem.2005.18.6.555  Weyers, S., De Sutter, P., Hoebeke, S., Monstrey, G., T Sjoen, G., Verstraelen, H., & Gerris, J. (2010). Gynaecological aspects of the treatment and follow-up of transsexual men and women. *Facts, views & vision in ObGyn*, *2*(1), 35-54. https://search.ebscohost.com/login.aspx?direct=true&db=cmedm&AN=25206965&site=ehost-live  Weyers, S., Elaut, E., De Sutter, P., Gerris, J., T'Sjoen, G., Heylens, G., De Cuypere, G., & Verstraelen, H. (2009). Long-term assessment of the physical, mental, and sexual health among transsexual women. *JOURNAL OF SEXUAL MEDICINE*, *6*(3), 752-760. https://doi.org/10.1111/j.1743-6109.2008.01082.x  Weyers, S., Lambein, K., Sturtewagen, Y., Verstraelen, H., Gerris, J., & Praet, M. (2010). Cytology of the 'penile' neovagina in transsexual women. *Cytopathology : official journal of the British Society for Clinical Cytology*, *21*(2), 111-115. https://doi.org/10.1111/j.1365-2303.2009.00663.x  Weyers, S., Verstraelen, H., Gerris, J., Monstrey, S., Santiago, G. d. S. L., Saerens, B., De Backer, E., Claeys, G., Vaneechoutte, M., & Verhelst, R. (2009). Microflora of the penile skin-lined neovagina of transsexual women. *BMC MICROBIOLOGY*, *9*, 102. https://doi.org/10.1186/1471-2180-9-102  Wierckx, K., Van Caenegem, E., Elaut, E., Dedecker, D., Van de Peer, F., Toye, K., Weyers, S., Hoebeke, P., Monstrey, S., De Cuypere, G., & T'Sjoen, G. (2011). Quality of life and sexual health after sex reassignment surgery in transsexual men. *JOURNAL OF SEXUAL MEDICINE*, *8*(12), 3379-3388. https://doi.org/10.1111/j.1743-6109.2011.02348.x  Wilchesky, M., Côté, H., Betito, L., & Assalian, P. (1994). Plasticity or stability of sexual orientation in male transsexuals. *CANADIAN JOURNAL OF HUMAN SEXUALITY*, *3*(4), 327-332. https://search.ebscohost.com/login.aspx?direct=true&db=psyh&AN=1996-02597-004&site=ehost-live  Winter, S., Rogando-Sasot, S., & King, M. (2008). Transgendered women of the Philippines. *INTERNATIONAL JOURNAL OF TRANSGENDERISM*, *10*(2), 79-90. https://doi.org/10.1080/15532730802182185  Wu, J.-x., Li, B., Liu, T., Li, W.-z., Jiang, Y.-g., Liang, J.-x., Wei, C.-s., Hu, H.-o., & Zhong, C.-x. (2009). Eighty-six cases of laparoscopic vaginoplasty using an ileal segment. *Chinese medical journal*, *122*(16), 1862-1866. https://search.ebscohost.com/login.aspx?direct=true&db=cmedm&AN=19781361&site=ehost-live |
